# Comparative safety and effectiveness of oral anticoagulants in key subgroups of patients with non-valvular atrial fibrillation and at high risk of gastrointestinal bleeding: A cohort study based on the French National Health Data System (SNDS)

**DOI:** 10.1371/journal.pone.0317895

**Published:** 2025-01-22

**Authors:** Gregory Y. H. Lip, Robert Benamouzig, Anne-Céline Martin, Giancarlo Pesce, Gaelle Gusto, Nadia Quignot, Artak Khachatryan, Feng Dai, Fouad Sedjelmaci, Jose Chaves, Rupesh Subash, Ruth Mokgokong

**Affiliations:** 1 Liverpool Centre for Cardiovascular Science at University of Liverpool, Liverpool John Moores University and Liverpool Heart & Chest Hospital, Liverpool, United Kingdom; 2 Danish Center for Health Services Research, Department of Clinical Medicine, Aalborg University, Aalborg, Denmark; 3 Hôpital Avicenne, Bobigny, France; 4 European Hospital Georges Pompidou, Paris, France; 5 INSERM UMRS_1140, University of Paris, Paris, France; 6 Certara Italy, Milan, Italy; 7 Certara France, Paris, France; 8 Certara UK, London, United Kingdom; 9 Pfizer Inc., Groton, New York, United States of America; 10 Pfizer SAS, Paris, France; 11 Pfizer SLU., Madrid, Spain; 12 Pfizer Ltd., Tadworth, United Kingdom; East Tennessee State University, UNITED STATES OF AMERICA

## Abstract

**Background:**

Risk factors and comorbidities can complicate management of non-valvular atrial fibrillation. We describe and compare real-world safety and effectiveness of direct oral anticoagulants (DOACs; apixaban, rivaroxaban, dabigatran) and vitamin K antagonists (VKAs) in subgroups of patients with non-valvular atrial fibrillation at high risk for gastrointestinal (GI) bleeding, utilizing data from a national quasi-exhaustive French database.

**Methods:**

Anticoagulant-naïve adults with non-valvular atrial fibrillation with ≥1 gastrointestinal bleeding risk factor, initiating anticoagulant treatment January 2016–December 2019, and covered by the French national health data system were eligible. The following subgroups were evaluated: patients age ≥75 years, receiving concomitant medications, HAS-BLED score ≥3, and chronic kidney disease stage 3–4. Outcomes included major bleeding and stroke/systemic embolism. Patient characteristics were balanced using propensity score matching.

**Results:**

A total of 314,184 patients were identified; characteristics were similar for propensity score-matched subgroups in VKA/DOAC and DOAC/DOAC comparisons. DOACs showed lower risk of major bleeding versus VKAs in all subgroups evaluated (*p*<0.0001 for all). Apixaban showed lower risk of major bleeding and gastrointestinal bleeding versus rivaroxaban in all subgroups (*p*≤0.05 for all) and versus dabigatran in elderly patients, patients with HAS-BLED score ≥3, and those receiving concomitant medications (*p*<0.05 for all). Stroke/systemic embolism risk was lower with apixaban versus rivaroxaban in elderly patients, those with HAS-BLED ≥3, and those receiving concomitant medications; risks were similar for other comparisons.

**Conclusions:**

DOACs were associated with improved safety and effectiveness when compared to VKAs among subgroups of non-valvular atrial fibrillation patients at high risk of gastrointestinal bleeding. Apixaban was associated with lower risks of major bleeding, gastrointestinal bleeding, and stroke/systemic embolism versus rivaroxaban as well as lower risk of major bleeding, gastrointestinal bleeding bleed and similar risk of stroke/systemic embolism versus dabigatran among several of these patient subgroups.

## Introduction

Oral anticoagulants are the mainstay of stroke prevention treatment for patients with non-valvular atrial fibrillation (NVAF). These include vitamin K antagonists (VKAs), such as warfarin, and direct oral anticoagulants (DOACs), such as apixaban, rivaroxaban, and dabigatran. Current guidelines recommend DOACs as a first treatment option; [1, 2] and in real-world clinical practice, these are increasingly used for the prevention of stroke and/or systemic embolism (SE) in patients with NVAF versus VKAs [3, 4]. However, bleeding risks due to anticoagulation present a therapeutic challenge [5]. Recent population-based studies have compared the effectiveness and safety of DOACs with VKAs in patients with NVAF to better understand their effects in actual conditions of use [6].

Additional risk factors and comorbidities can complicate NVAF management. NVAF often occurs in patients who have other comorbidities, such as frailty, decreased renal function or recent bleeding, leading to clinical complexity [7]. For example, chronic kidney disease (CKD) is associated with increased incidence/prevalence of NVAF, and NVAF is associated with an increased risk of CKD [8]. Patients with CKD are often underrepresented in randomized clinical trials of anticoagulants and evidence in this population is scarce. Similarly, elderly patients with NVAF may be excluded from clinical trials; [9] these patients are also at high risk for bleeding. Patients who receive concomitant medication, such as antiplatelet drugs or non-steroidal anti-inflammatory drugs (NSAIDs), may also have an increased risk of bleeding events [10]. These factors can all play a role in treatment decision-making.

Our understanding of safety and effectiveness in high-risk populations of patients with NVAF is limited. Real-world data can provide insights to managing clinically complex patients, as there have not been any head-to-head randomized clinical trials of DOACs in some of these high-risk subgroups, and current studies of anticoagulants may exclude patients with additional risk factors. Understanding the differences in outcomes between specific patient subgroups can better inform the practicing clinician’s ability to offer the best anticoagulation options to individual patients. The objective of this study was to describe and compare the safety and effectiveness of oral anticoagulants (apixaban, rivaroxaban, dabigatran, or VKAs) in key subgroups of patients with NVAF at high risk for GIB, including those age ≥75 years, those receiving concomitant antiplatelets, NSAIDs, or corticosteroids, those with HAS-BLED score ≥3, and those with CKD stage 3–4, utilizing recent data from a French national quasi-exhaustive healthcare database.

## Methods

### Study design and data source

This was a historical, population-based cohort study (NCT05038228) using data extracted from the French national health data system (*Système National des Données de Santé*; SNDS), which covers 99% of the French population [11]. Data are linked via a unique social security number to primary care, hospital, pharmacy, and death registration databases, allowing for tracking of patient treatment history, treatment patterns, and hospitalizations based on International Classification of Diseases, Tenth Revision (ICD-10) codes. Data spanning over a 6-year period from 1st January 2014 to 31st December 2019 (study period) were used. The identification period was January 1, 2016 to December 31, 2019; index date was defined as the first anticoagulant reimbursement during the identification period. Follow-up was evaluated from the day after index date to censoring due to treatment switch, treatment discontinuation, treatment interruption, death, pregnancy, dialysis, chronic kidney disease stage V, or end of study. Baseline characteristics, comorbidities, and prior anticoagulant use (to ensure population is AC-treatment naïve) were evaluated during the baseline period (during 24 months prior to and on the index date) using ICD-10 codes (principal or associated) and/or procedure and/or ATC codes, as relevant. Concomitant treatments were identified using ATC codes and evaluated 3 months prior to and including the index date. Treatment discontinuation was defined as >30 days after coverage by the last AC dispensation without refilling. Treatment interruption was defined as a patient having a gap with no new treatment within 30 days of the estimated end of supply, but subsequently restarts the index treatment after this period. Patients who had a reimbursement for an anticoagulant other than the index drug reimbursement during the follow-up period will be considered switchers. Data were accessed for research purposes on 9 June 2022. Authors did not have access to information that could identify individual participants during or after data collection. Institutional review board approval was not needed as all data were de-identified and analyzed in aggregate. The authors were authorized by the French national data protection authority (Commission Nationale de l’Informatique et des Libertés, CNIL) to access the data underlying this study (decision DR-2021-130).

### Study population

The study population included all anticoagulant-treatment naive adult patients (≥18 years old) diagnosed with NVAF, with high risk of gastrointestinal bleeding (GIB), and having a newly initiated anticoagulant treatment (index anticoagulant—apixaban, dabigatran, rivaroxaban or VKAs) during the identification period, i.e., from January 1, 2016 to December 31, 2019. Patients presenting ≥1 of the following indicators were considered as treated with anticoagulant for an indication of NVAF: (1) diagnoses identified through “Long Term Diseases” (LTD) registry in the 24 months before and the 30 days after index date using the ICD-10 code I48 (atrial fibrillation and flutter); (2) main or associated diagnoses of hospitalizations in the 24 months before index date using the ICD-10 code I48; and (3) prescription of any anti-arrhythmic drugs (Anatomical Therapeutic Chemical code [ATC]: C01B*) dispensed concomitantly with VKAs or DOACs, i.e., dispensed between 6 weeks before and 15 days after index date.

Comparative safety and effectiveness of following subgroups of NVAF patients at high risk of GIB were evaluated: patients age ≥75 years at index date, patients using concomitant antiplatelets, NSAIDs, or corticosteroids 6 weeks prior to and including index date, patients with HAS-BLED score ≥3 at index date, and patients with CKD stage 3 or 4 at index date. The study population included patients who met selection criteria and received treatment with apixaban, dabigatran, rivaroxaban, or VKAs after their initial index encounter. VKAs included warfarin, acenocoumarol, or fluindione. Of note, edoxaban was not included as it is not available in France.

**Fig 1:**
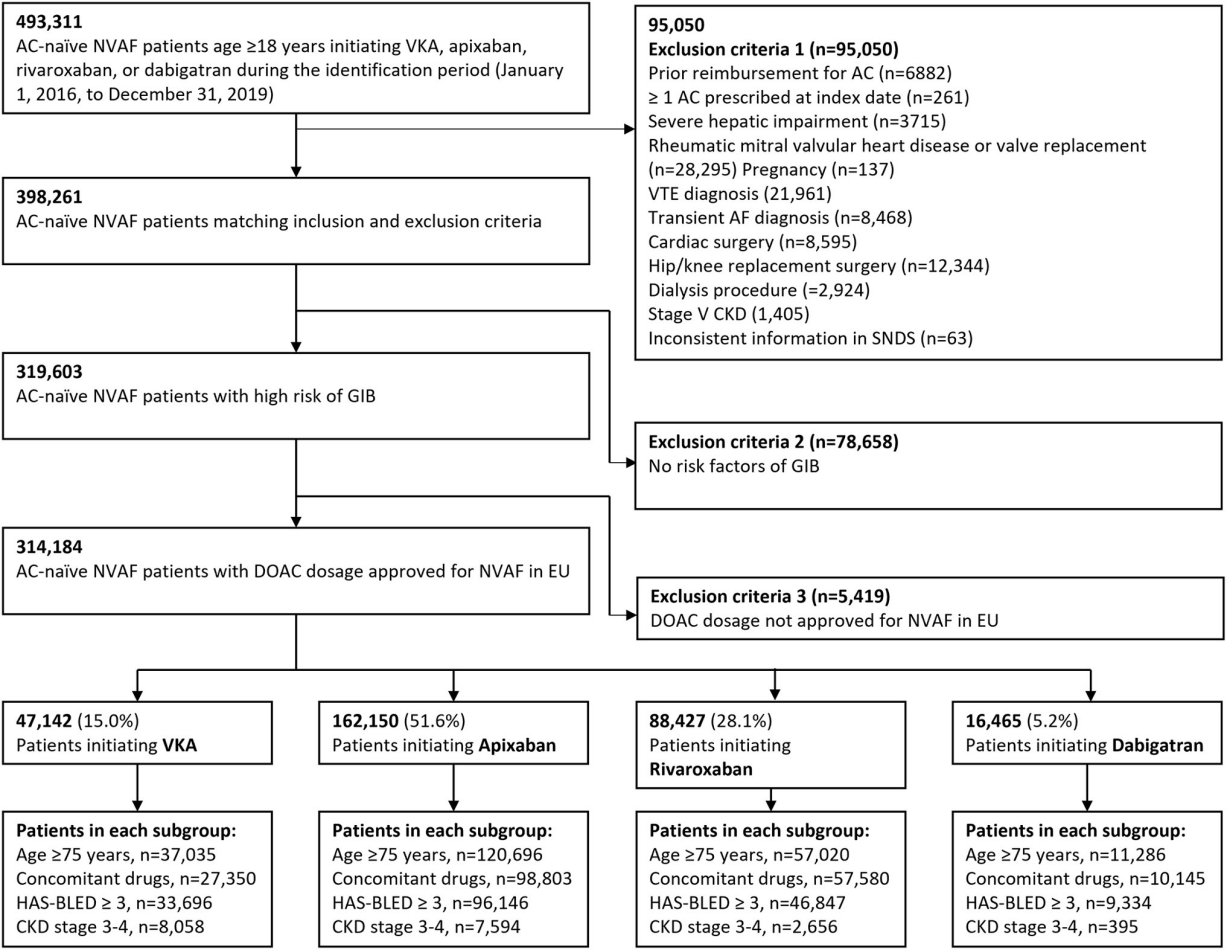
Patient selection criteria.

Patients were excluded if they had: (1) ≥1 reimbursement for an anticoagulant treatment within 24 months prior to the index date; (2) patient prescribed with more than one type of anticoagulant treatments at the index date (e.g. both VKA and apixaban); (3) mixed DOAC dosage at index date (e.g., apixaban 5 mg + 2.5 mg) or DOAC dosage not approved for NVAF in the European Union (e.g., rivaroxaban 10 mg or dabigatran 75 mg); (4) severe hepatic impairment within 24 months prior to the index date; (5) rheumatic mitral valvular heart disease or a valve replacement procedure within 24 months prior to the index date; or (6) evidence of pregnancy in the 9 months prior to the index date. Full exclusion criteria are presented in Fig 1.

### Outcome measures

The primary safety outcomes included major bleeding leading to hospitalization, overall and by site (GIB, ICH, and other). The primary effectiveness outcomes included stroke (ischemic or hemorrhagic) or SE, overall and separately (S1 Table). Both effectiveness and safety outcomes were identified with primary diagnosis and evaluated from the day after the index date until end of the follow-up period.

### Statistical analysis

A 1:1 propensity score (PS) matching technique utilizing binary logistic regression was used as the primary method to balance patient characteristics between the cohorts. PS was defined as the probability of a patient receiving a certain treatment conditional on their observed baseline covariates. Covariates were evaluated during the baseline period (during 24 months prior to and on the index date) using ICD-10 codes (principal or associated) and/or procedure and/or ATC codes, as relevant. Covariates included sociodemographic characteristics (age, gender), clinical charateristics, and concomitant medication use.

PS matching was performed for six different groups: apixaban versus VKAs, apixaban versus dabigatran, apixaban versus rivaroxaban, rivaroxaban versus dabigatran, rivaroxaban versus VKAs, and dabigatran versus VKAs. Two patients from two cohorts were matched using a sequential pairwise nearest neighbor approach, if the difference in the logit of PS between them was equal to 0.2 times the standard deviation of the logit of the PS. Several checks were performed to ensure a good balance of PS and of covariates between treatment groups: first, the treatment group PS distribution was analyzed graphically using a love plot; second, the balance of covariates across treatment and comparison groups was checked using the absolute standardized difference (with a threshold of <10%).

Baseline demographic and clinical characteristics were presented using descriptive statistics for both the pre- and post-PS-matched between-group comparisons, overall and by index anticoagulant treatment. Kaplan-Meier methodology was used to estimate time-to-event of first occurrence of each outcome of interest and cumulative incidence curves after PS matching. To compare risk, the cumulative incidence rates for clinical outcomes, censored at treatment non-persistence, chronic kidney disease stage V, dialysis, pregnancy, death, or end of the study were calculated as the number of patients who experienced the event divided by the observed time at risk expressed per 100 person-years (including 95% confidence interval [CI] within each cohort). Using Cox proportional hazard models, hazard ratios (HR) and associated 95% CI were calculated for each studied event (major bleeding [overall, GI, ICH, other] and stroke/SE [overall, hemorrhagic, ischemic, SE]). Proportional hazard assumption was verified visually and using Schoenfeld residuals plots. Statistical significance was set at *p*<0.05, and all tests were 2-tailed. All analyses were conducted using SAS Statistical Package (SAS guide 8) or R (version 4.1.3).

For each subgroup comparison, the PS was performed and evaluated to ensure a good balance of covariates between the groups compared. The same PS was used but the subgroup populations were not restricted to those matched in the overall population [12], allowing for the maximum subgroup population size. All subgroups were re-matched, with the exception of patients age ≥75 years as this subgroup was already balanced after the original PS matching [12]. Comparative time-to-event analyses for each studied effectiveness and safety outcomes were performed adjusted on PS using a Cox proportional hazard model. Comparisons with dabigatran were not performed within the subgroup of patients with CKD stage 3–4 due to low sample size.

### Sensitivity analyses

Sensitivity analyses of comparative safety and effectiveness were also conducted using accelerated failure time (AFT) models to compare results with the Cox model due to proportional hazards violations [13]. Gamma, Weibull, exponential, log normal, and log logistic distributions were considered to model the data. The distribution with the lowest Akaike information criterion was retained.

## Results

### Patient selection and characteristics

Between January 1, 2016 and December 31, 2019, a total of 493,311 anticoagulant-naïve patients initiating treatment were identified in the SNDS. A total of 314,184 patients were included in the final study population of patients with NVAF at high risk of GIB after applying the selection criteria (Fig 1). A total of 226,037 (71.9%) patients were age ≥75 years, 186,023 (59.2%) had a HAS-BLED score ≥3, 193,868 (61.7%) were receiving medications associated with GI bleeding risk (antiplatelets, NSAIDs, or corticosteroids), and 18,703 (6.0%) had renal impairment (CKD stage 3 or 4).

### Risk of bleeding and stroke/SE

#### PS matched cohorts

**Fig 2:**
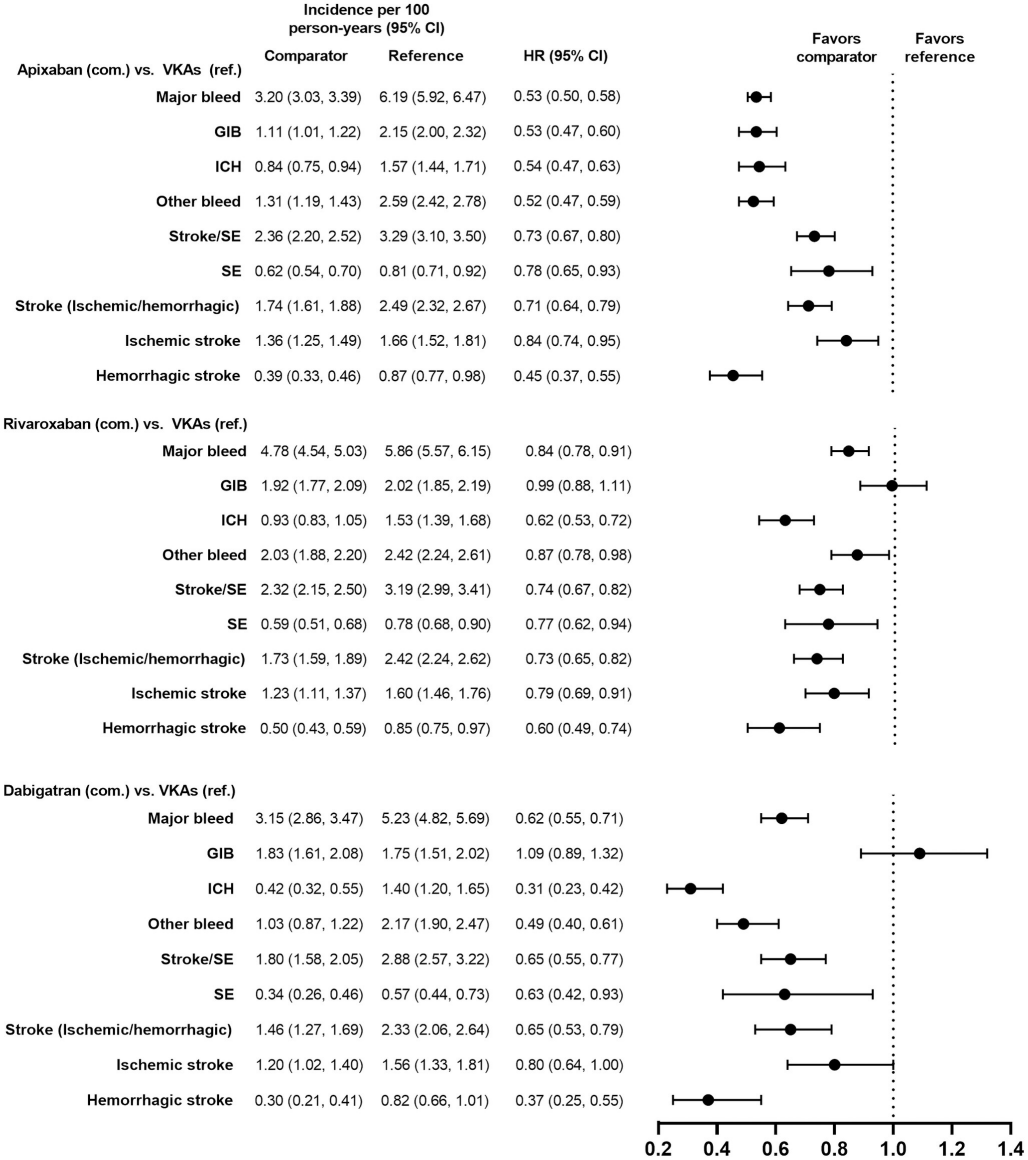
PS-matched hazard ratios among patients age ≥75 years for DOACs versus VKAs.

**Fig 3:**
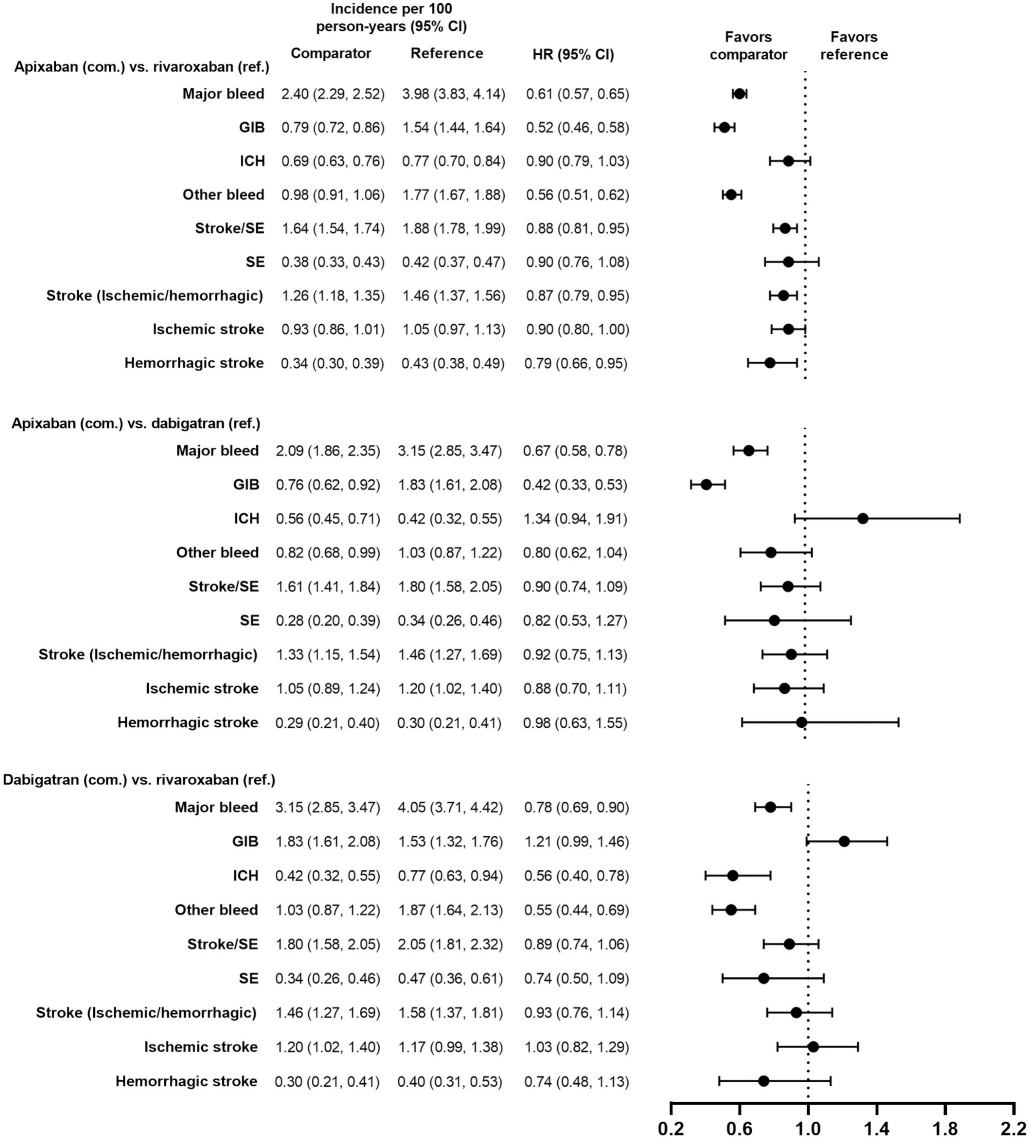
PS-matched hazard ratios among patients age ≥75 years for DOACs versus DOACs.

**Fig 4:**
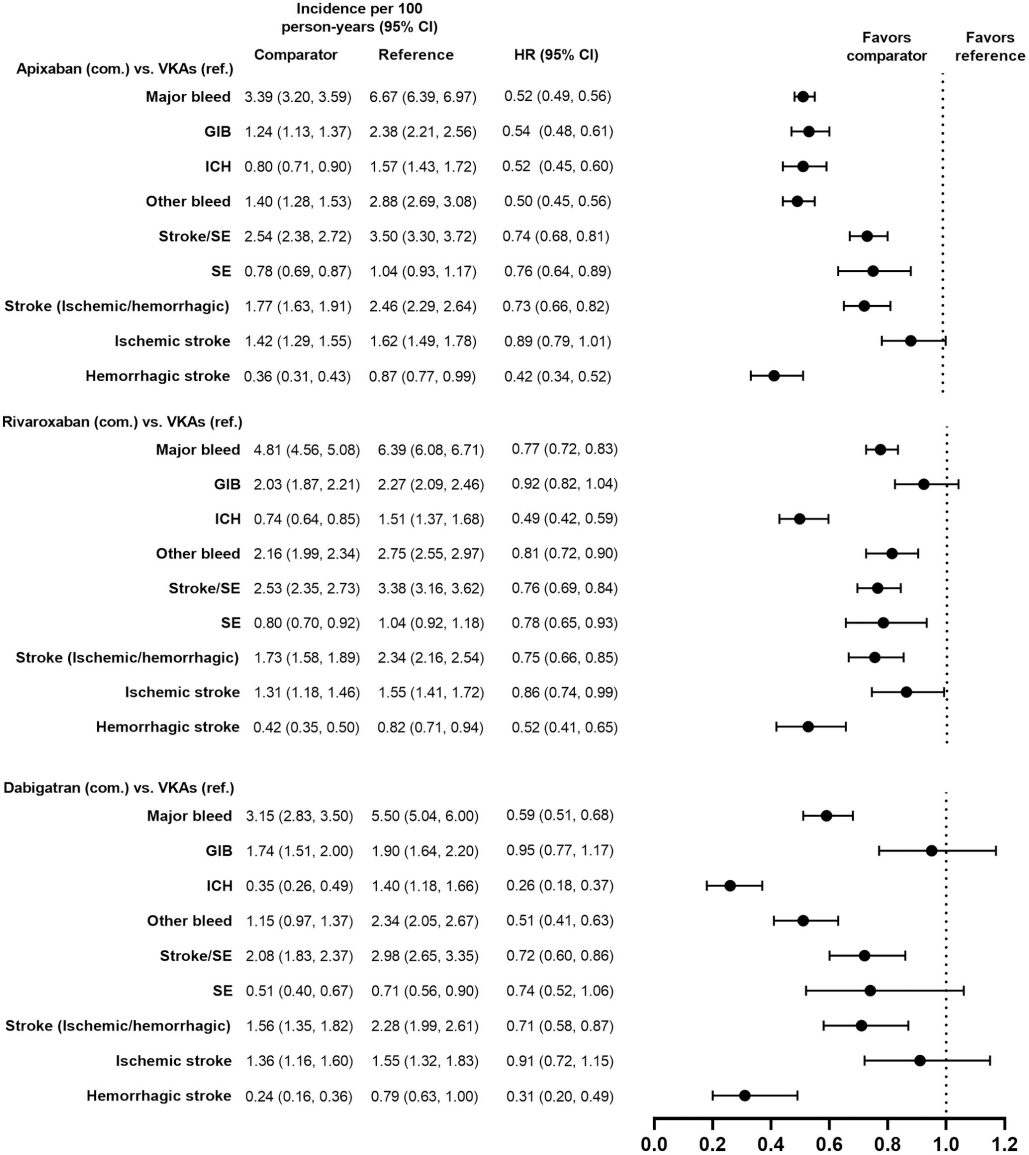
PS-matched hazard ratios among patients with HAS-BLED score ≥3 for DOACs versus VKAs.

**Fig 5:**
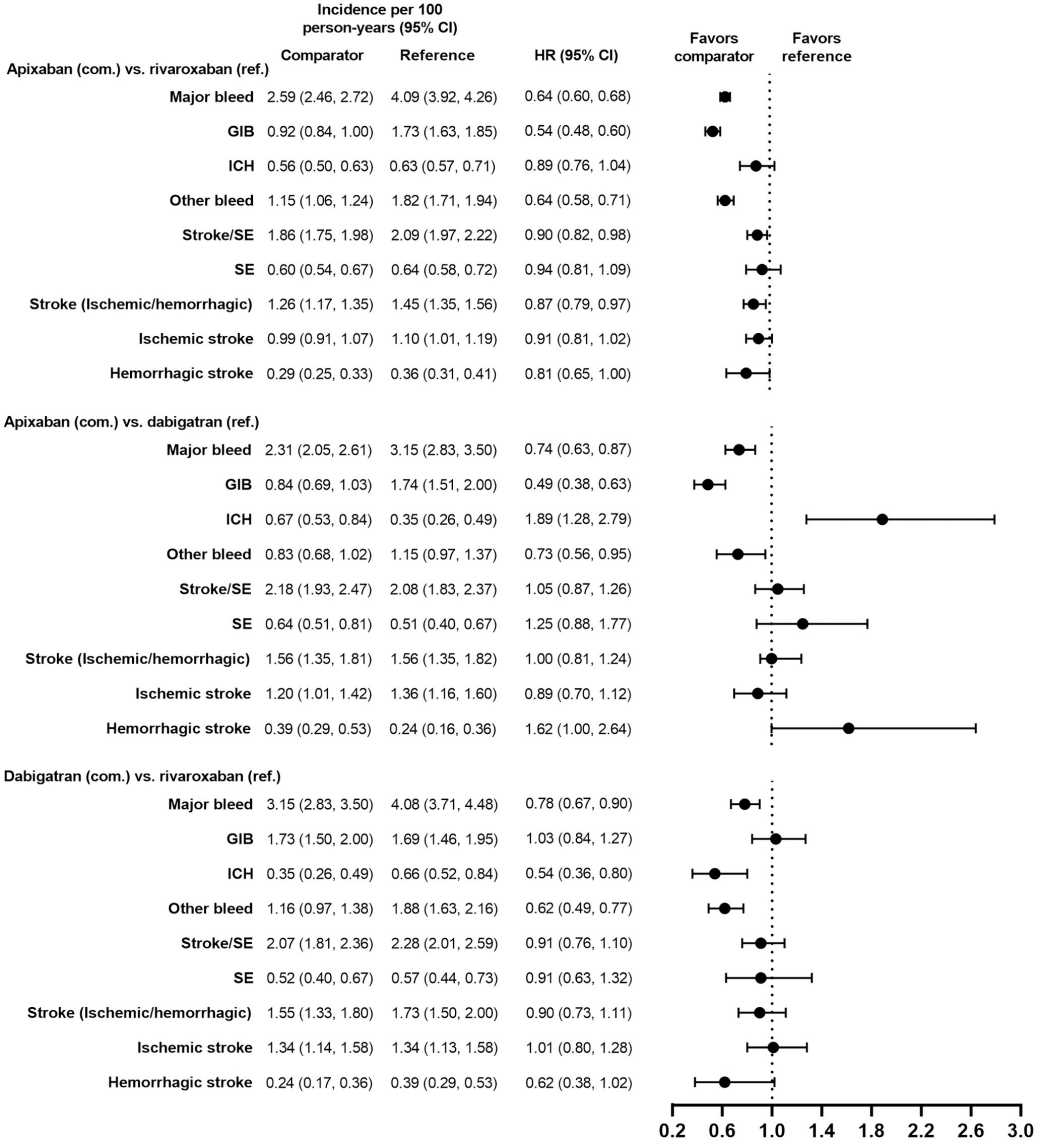
PS-matched hazard ratios among patients with HAS-BLED score ≥3 for DOACs versus DOACs.

**Fig 6:**
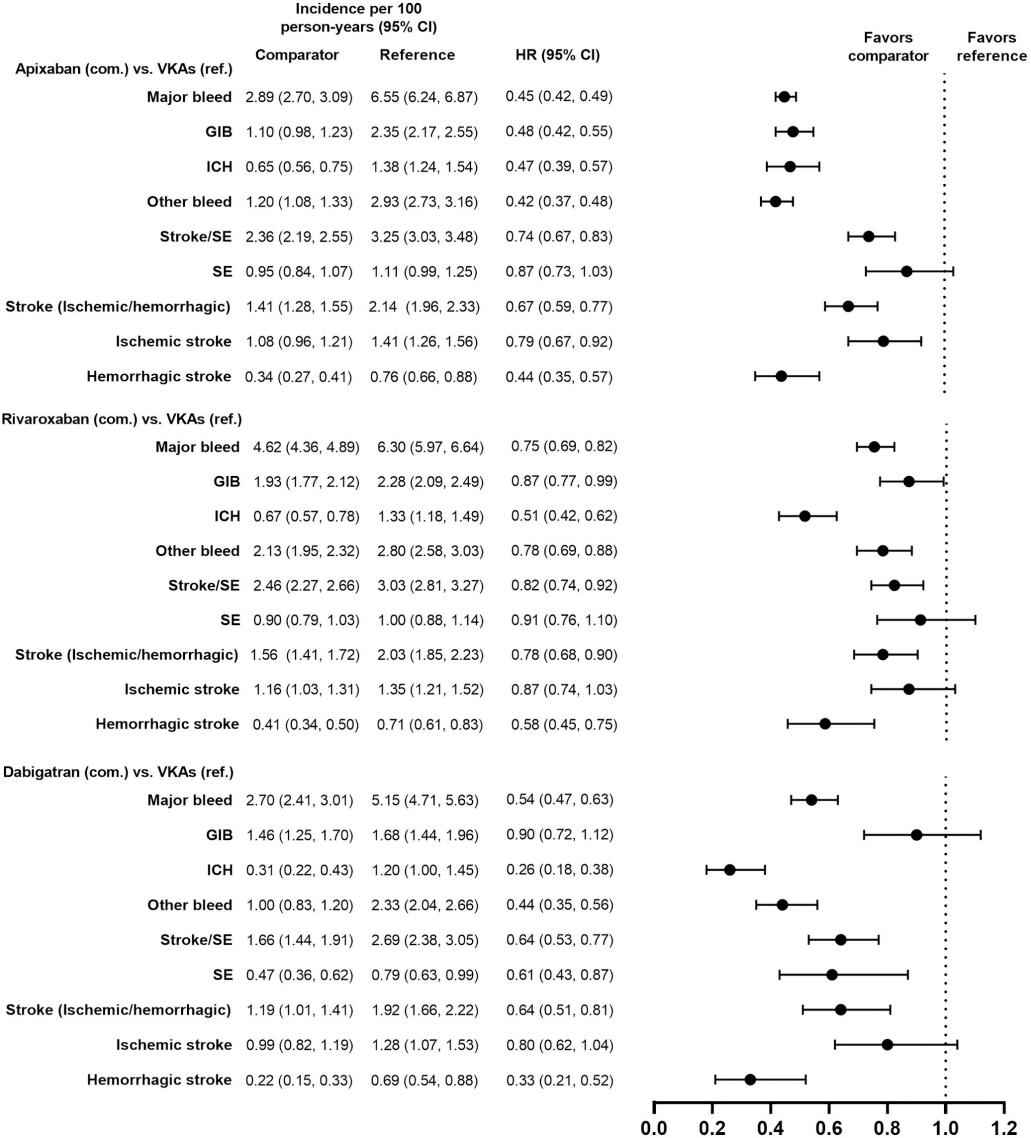
PS-matched hazard ratios among patients receiving concomitant antiplatelets, NSAIDs, or corticosteroids associated with GIB risk for DOACs versus VKAs.

**Fig 7:**
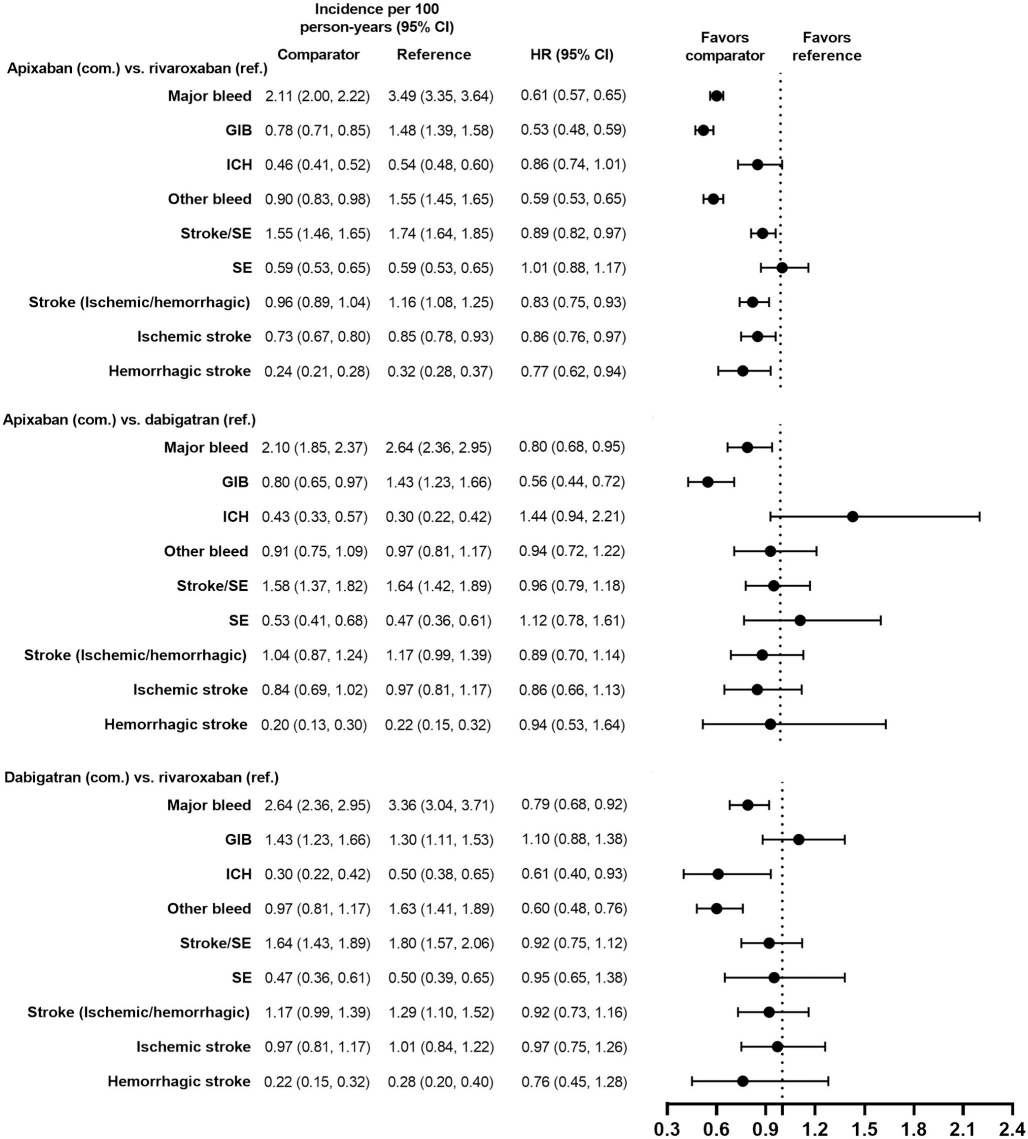
PS-matched hazard ratios among patients receiving concomitant antiplatelets, NSAIDs, or corticosteroids associated with GIB risk for DOACs versus DOACs.

**Fig 8:**
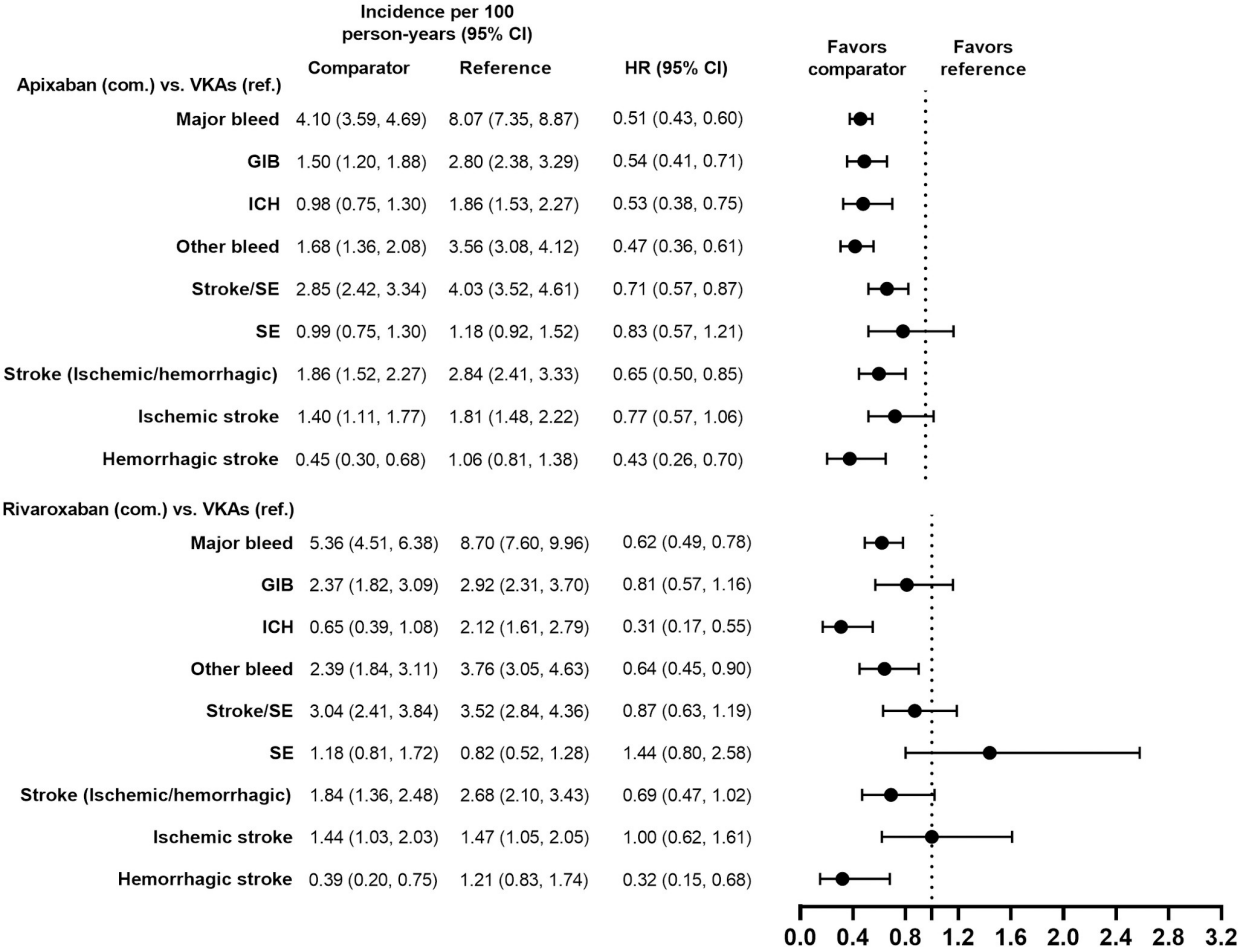
PS-matched hazard ratios among patients with CKD stage 3–4 for apixaban versus VKAs and rivaroxaban versus VKAs.

**Fig 9:**
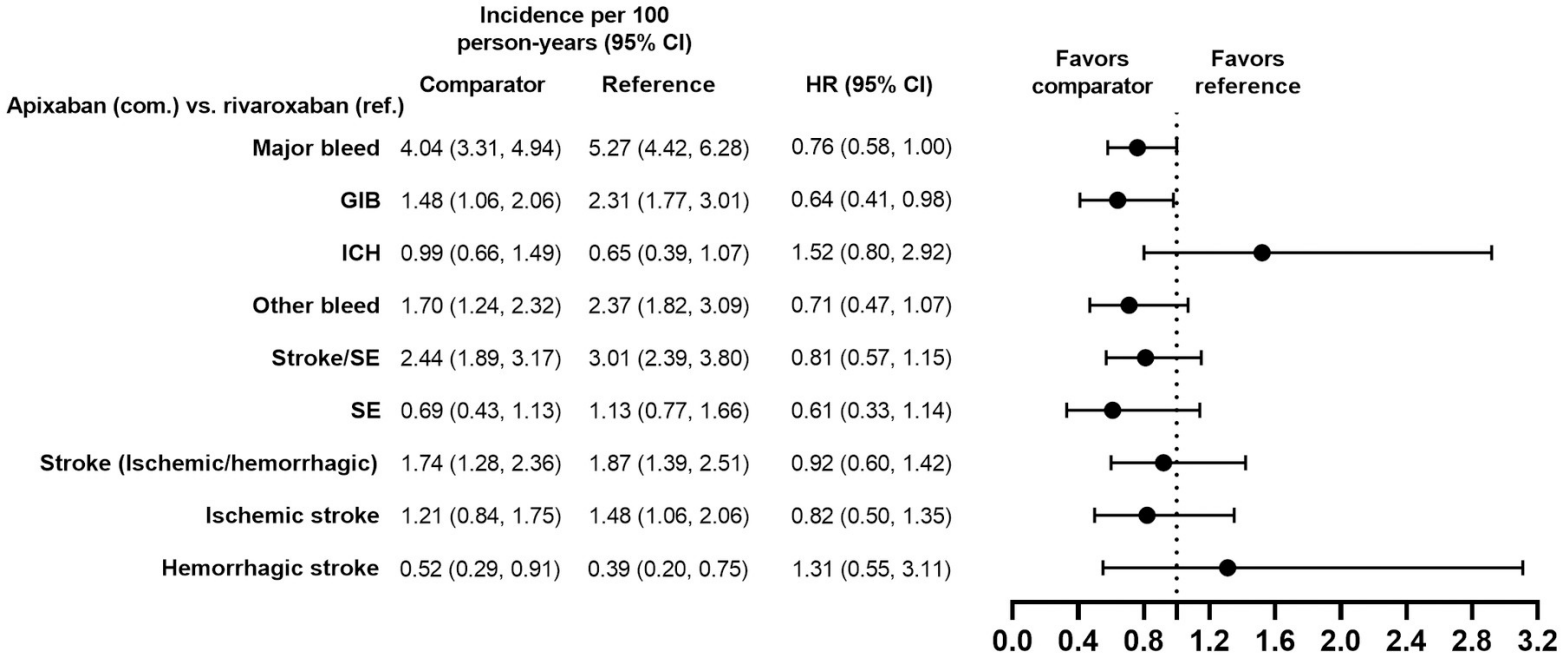
PS-matched hazard ratios among patients with CKD stage 3–4 for apixaban versus rivaroxaban.

Demographic and clinical characteristics were similar (i.e., absolute standardized difference <10%) for the PS-matched subgroups in the VKAs versus DOACs comparison and in the DOACs versus DOACs comparison (Tables 1–4). Demographic and clinical characteristics prior to PS matching are presented for each subgroup in S2–S5 Tables. Incidence rates of major bleeding in the PS-matched subgroups are presented in Figs 2–9.

**Table 1:** Baseline characteristics after PS matching for patients age ≥75 years.

		Pairwise comparison		Pairwise comparison		Pairwise comparison		Pairwise comparison		Pairwise comparison		Pairwise comparison	
Characteristic		Apixaban (n = 34,007)	VKAs (n = 35,329)	Rivaroxaban (n = 28,318)	VKAs (n = 29,549)	Dabigatran (n = 11,278)	VKAs (n = 11,634)	Apixaban (n = 57,612)	Rivaroxaban (n = 57,019)	Dabigatran (n = 11,204)	Rivaroxaban (n = 11,284)	Apixaban (n = 11,249)	Dabigatran (n = 11,286)
**Index dosage**	Standard dose	-	-	-	-	-	-	30071 (52.2%)	30358 (53.24%)	2657 (23.71%)	2250 (19.94%)	2409 (21.42%)	2250 (19.94%)
	Reduced dose	-	-	-	-	-	-	27541 (47.8%)	26661 (46.76%)	8547 (76.29%)	9034 (80.06%)	8840 (78.58%)	9036 (80.06%)
**Atrial fibrillation identification setting**	Inpatient claim with I48 code	26976 (79.32%)	27555 (78%)	21509 (75.96%)	22069 (74.69%)	6467 (57.34%)	6511 (55.97%)	32639 (56.65%)	32958 (57.8%)	6193 (55.27%)	6466 (57.3%)	6203 (55.14%)	6468 (57.31%)
	LTR registration with I48 code*	1445 (4.25%)	1635 (4.63%)	1573 (5.55%)	1617 (5.47%)	1306 (11.58%)	1292 (11.11%)	6551 (11.37%)	6275 (11.01%)	1346 (12.01%)	1309 (11.6%)	1361 (12.1%)	1309 (11.6%)
	Use of anti-arrhythmic drugs*	5586 (16.43%)	6139 (17.38%)	5236 (18.49%)	5863 (19.84%)	3505 (31.08%)	3831 (32.93%)	18422 (31.98%)	17786 (31.19%)	3665 (32.71%)	3509 (31.1%)	3685 (32.8%)	3509 (31.1)
**Follow up time (months), censored at switch, discontinuation, interruption, death, pregnancy, dialysis, CKD stage V, or end of follow up, mean (SD)**		12.8 [12]	10.5 [11.2]	12.5 [12.2]	10.7 [11.4]	13.3 [12.6]	10.8 [11.7]	13.8 [12.7]	12.9 [12.6]	13.3 [12.6]	13.0 [12.7]	14.0 [12.9]	13.3 [12.6]
**Age at index date (years), mean (SD)**		85.2 (5.8)	85.6 (5.8)	84.8 (5.7)	85.2 (5.7)	83.3 (5.3)	83.4 (5.3)	83.1 (5.4)	83 (5.4)	83.3 (5.3)	83.3 (5.3)	83.5 (5.3)	83.3 (5.3)
	75–79 years*	6557 (19.28%)	6087 (17.23%)	5822 (20.56%)	5672 (19.20%)	3175 (28.15%)	3175 (27.29%)	17661 (30.66%)	17456 (30.61%)	3172 (28.31%)	3180 (28.18%)	3147 (27.98%)	3182 (28.19%)
	≥80 years*	27450 (80.72%)	29242 (82.77%)	22496 (79.44%)	23877 (80.80%)	8103 (71.85%)	8459 (72.71%)	39951 (69.34%)	39563 (69.39%)	8032 (71.69%)	8104 (71.82%)	8102 (72.02%)	8104 (71.81%)
**Sex**	Male	14536 (42.74%)	14705 (41.62%)	12373 (43.69%)	12347 (41.78%)	4993 (44.27%)	5184 (44.56%)	25832 (44.84%)	25565 (44.84%)	4885 (43.60%)	4998 (44.29%)	4815 (42.80%)	4999 (44.29%)
	Female*	19471 (57.26%)	20624 (58.38%)	15945 (56.31%)	17202 (58.22%)	6285 (55.73%)	6450 (55.44%)	31780 (55.16%)	31454 (55.16%)	6319 (56.4%)	6286 (55.71%)	6434 (57.2%)	6287 (55.71%)
**GIB risk factors**	Age ≥75 years	34007 (100%)	35329 (100%)	28318 (100%)	29549 (100%)	11278 (100%)	11634 (100%)	57612 (100%)	57019 (100%)	11204 (100%)	11284 (100%)	11249 (100%)	11286 (100%)
	HAS-BLED score, mean (SD)	3.1 (1)	3.1 (1.1)	2.9 (1)	2.9 (1)	2.7 (0.9)	2.6 (0.9)	2.6 (0.9)	2.6 (0.9)	2.6 (0.9)	2.7 (0.9)	2.6 (0.9)	2.7 (0.9)
	0	0	0	0	0	0	0	0	0	0	0	0	0
	1	1433 (4.21%)	1864 (5.28%)	1617 (5.71%)	1864 (6.31%)	1158 (10.27%)	1187 (10.2%)	6333 (10.99%)	6263 (10.98%)	1152 (10.28%)	1158 (10.26%)	1196 (10.63%)	1158 (10.26%)
	2	7513 (22.09%)	8770 (24.82%)	7843 (27.7%)	8631 (29.21%)	3748 (33.23%)	3961 (34.05%)	20442 (35.48%)	19835 (34.79%)	3797 (33.89%)	3750 (33.23%)	3876 (34.46%)	3751 (33.24%)
	≥3*	25061 (73.69%)	24695 (69.9%)	18858 (66.59%)	19054 (64.48%)	6372 (56.5%)	6486 (55.75%)	30837 (53.53%)	30921 (54.23%)	6255 (55.83%)	6376 (56.5%)	6177 (54.91%)	6377 (56.5%)
	Prior medications (antiplatelets, NSAIDs, or corticosteroids)	19426 (57.12%)	18509 (52.39%)	15813 (55.84%)	15431 (52.22%)	5793 (51.37%)	5882 (50.56%)	29944 (51.98%)	29996 (52.61%)	5713 (50.99%)	5799 (51.39%)	5719 (50.84%)	5800 (51.39%)
	Renal impairment (CKD stage 3–4)	4886 (14.37%)	5233 (14.81%)	2085 (7.36%)	2424 (8.2%)	319 (2.83%)	343 (2.95%)	2099 (3.64%)	2110 (3.7%)	274 (2.45%)	319 (2.83%)	285 (2.53%)	319 (2.83%)
	Prior GI condition*	3200 (9.41%)	2853 (8.08%)	2268 (8.01%)	2227 (7.54%)	793 (7.03%)	749 (6.44%)	3465 (6.01%)	3519 (6.17%)	677 (6.04%)	792 (7.02%)	696 (6.19%)	793 (7.03%)
**Number of GIB risk factors**	1	6308 (18.55%)	8597 (24.33%)	7112 (25.11%)	8492 (28.74%)	3875 (34.36%)	4091 (35.16%)	20997 (36.45%)	20296 (35.6%)	3977 (35.5%)	3876 (34.35%)	4006 (35.61%)	3876 (34.34%)
	2	7347 (21.6%)	6678 (18.9%)	5750 (20.31%)	5512 (18.65%)	2105 (18.66%)	2170 (18.65%)	9763 (16.95%)	9789 (17.17%)	2007 (17.91%)	2106 (18.66%)	2098 (18.65%)	2107 (18.67%)
	3	16117 (47.39%)	15821 (44.78%)	13223 (46.69%)	13127 (44.42%)	4747 (42.09%)	4841 (41.61%)	24093 (41.82%)	24174 (42.4%)	4763 (42.51%)	4751 (42.1%)	4670 (41.51%)	4752 (42.11%)
	4	3948 (11.61%)	3962 (11.21%)	2104 (7.43%)	2302 (7.79%)	526 (4.66%)	520 (4.47%)	2640 (4.58%)	2631 (4.61%)	442 (3.95%)	526 (4.66%)	461 (4.1%)	526 (4.66%)
	5	287 (0.84%)	271 (0.77%)	129 (0.46%)	116 (0.39%)	25 (0.22%)	12 (0.1%)	119 (0.21%)	129 (0.23%)	15 (0.13%)	25 (0.22%)	14 (0.12%)	25 (0.22%)
**Charlson Comorbidity Index score**	Mean (SD)	2.7 [2.2]	2.5 [2.2]	2.2 [2.0]	2.1 [2.0]	1.5 [1.9]	1.5 [1.8]	1.4 [1.8]	1.5 [1.8]	1.5 [1.9]	1.5 [1.8]	1.4 [1.8]	1.5 [1.9]
	0	4612 (13.56%)	6233 (17.64%)	4978 (17.58%)	6200 (20.98%)	4007 (35.53%)	4214 (36.22%)	21318 (37%)	20558 (36.05%)	4179 (37.3%)	4013 (35.56%)	4295 (38.18%)	4013 (35.56%)
	1 or 2	14298 (42.04%)	14520 (41.1%)	13824 (48.82%)	13682 (46.3%)	4775 (42.34%)	4948 (42.53%)	25274 (43.87%)	25065 (43.96%)	4661 (41.6%)	4776 (42.33%)	4664 (41.46%)	4777 (42.33%)
	3 or 4	9385 (27.60%)	9201 (26.04%)	6407 (22.63%)	6666 (22.56%)	1756 (15.57%)	1781 (15.31%)	7659 (13.29%)	7934 (13.91%)	1671 (14.91%)	1755 (15.55%)	1638 (14.56%)	1756 (15.56%)
	≥5	5712 (16.80%)	5375 (15.21%)	3109 (10.98%)	3001 (10.16%)	740 (6.56%)	691 (5.94%)	3361 (5.83%)	3462 (6.07%)	693 (6.19%)	740 (6.56%)	652 (5.8%)	740 (6.56%)
**Comorbidities**	Myocardial infarction*	3475 (10.22%)	3358 (9.5%)	2543 (8.98%)	2467 (8.35%)	514 (4.56%)	470 (4.04%)	3284 (5.7%)	3281 (5.75%)	470 (4.19%)	514 (4.56%)	440 (3.91%)	514 (4.55%)
	Congestive heart failure*	15410 (45.31%)	16228 (45.93%)	11606 (40.98%)	11954 (40.45%)	2610 (23.14%)	2455 (21.1%)	14721 (25.55%)	15066 (26.42%)	2544 (22.71%)	2610 (23.13%)	2402 (21.35%)	2610 (23.13%)
	Peripheral vascular disease*	4442 (13.06%)	4136 (11.71%)	2930 (10.35%)	2900 (9.81%)	717 (6.36%)	632 (5.43%)	3544 (6.15%)	3697 (6.48%)	637 (5.69%)	716 (6.35%)	615 (5.47%)	717 (6.35%)
	Cerebrovascular disease*	7437 (21.87%)	6406 (18.13%)	5215 (18.42%)	4917 (16.64%)	2134 (18.92%)	2116 (18.19%)	6707 (11.64%)	6827 (11.97%)	2153 (19.22%)	2134 (18.91%)	2037 (18.11%)	2135 (18.92%)
	Dementia*	5035 (14.81%)	4849 (13.73%)	3716 (13.12%)	3801 (12.86%)	806 (7.15%)	815 (7.01%)	4718 (8.19%)	4873 (8.55%)	756 (6.75%)	805 (7.13%)	780 (6.93%)	806 (7.14%)
	Chronic pulmonary disease*	8451 (24.85%)	7708 (21.82%)	6672 (23.56%)	6283 (21.26%)	2096 (18.58%)	2101 (18.06%)	11033 (19.15%)	11107 (19.48%)	1944 (17.35%)	2097 (18.58%)	1971 (17.52%)	2097 (18.58%)
	Connective tissue disease*	787 (2.31%)	641 (1.81%)	542 (1.91%)	502 (1.7%)	142 (1.26%)	145 (1.25%)	752 (1.31%)	757 (1.33%)	143 (1.28%)	142 (1.26%)	147 (1.31%)	142 (1.26%)
	Ulcer disease*	510 (1.5%)	508 (1.44%)	299 (1.06%)	348 (1.18%)	101 (0.9%)	104 (0.89%)	372 (0.65%)	360 (0.63%)	87 (0.78%)	101 (0.9%)	94 (0.84%)	101 (0.89%)
	Mild liver disease*	590 (1.73%)	490 (1.39%)	375 (1.32%)	366 (1.24%)	76 (0.67%)	90 (0.77%)	433 (0.75%)	440 (0.77%)	73 (0.65%)	76 (0.67%)	65 (0.58%)	76 (0.67%)
	Diabetes*	7952 (23.38%)	7807 (22.1%)	6061 (21.4%)	6111 (20.68%)	1867 (16.55%)	2002 (17.21%)	10229 (17.75%)	10300 (18.06%)	1862 (16.62%)	1867 (16.55%)	1821 (16.19%)	1867 (16.54%)
	Diabetes with end-organ damage*	1222 (3.59%)	1265 (3.58%)	635 (2.24%)	658 (2.23%)	120 (1.06%)	125 (1.07%)	709 (1.23%)	672 (1.18%)	97 (0.87%)	120 (1.06%)	1821 (16.19%)	1867 (16.54%)
	Hemiplegia*	3073 (9.04%)	2597 (7.35%)	2030 (7.17%)	1942 (6.57%)	954 (8.46%)	980 (8.42%)	2496 (4.33%)	2397 (4.2%)	994 (8.87%)	953 (8.45%)	864 (7.68%)	954 (8.45%)
	Moderate or severe renal disease	7684 (22.6%)	8483 (24.01%)	3448 (12.18%)	4067 (13.76%)	550 (4.88%)	634 (5.45%)	3505 (6.08%)	3542 (6.21%)	489 (4.36%)	550 (4.87%)	508 (4.52%)	550 (4.87%)
	Any tumor (except for malignant neoplasm of skin)	3800 (11.17%)	3323 (9.41%)	2987 (10.55%)	2615 (8.85%)	912 (8.09%)	853 (7.33%)	4090 (7.1%)	4321 (7.58%)	831 (7.42%)	912 (8.08%)	801 (7.12%)	912 (8.08%)
	Metastatic solid tumor*	667 (1.96%)	592 (1.68%)	582 (2.06%)	507 (1.72%)	178 (1.58%)	160 (1.38%)	718 (1.25%)	784 (1.37%)	150 (1.34%)	178 (1.58%)	146 (1.3%)	178 (1.58%)
	HIV/ AIDS*	23 (0.07%)	20 (0.06%)	13 (0.05%)	13 (0.04%)	4 (0.04%)	0 (0.00%)	16 (0.03%)	13 (0.02%)	3 (0.03%)	4 (0.04%)	3 (0.03%)	4 (0.04%)
	Moderate or severe liver disease*	128 (0.38%)	115 (0.33%)	60 (0.21%)	78 (0.26%)	18 (0.16%)	19 (0.16%)	81 (0.14%)	63 (0.11%)	15 (0.13%)	18 (0.16%)	15 (0.13%)	18 (0.16%)
	Hypertension	30191 (88.78%)	31553 (89.31%)	24785 (87.52%)	25936 (87.77%)	9280 (82.28%)	9589 (82.42%)	46983 (81.55%)	46609 (81.74%)	9284 (82.28%)	9284 (82.86%)	9194 (81.73%)	9286 (82.28%)
	Diabetes mellitus	8705 (25.6%)	8626 (24.42%)	6649 (23.48%)	6741 (22.81%)	2034 (18.04%)	2176 (18.7%)	11008 (19.11%)	11141 (19.54%)	2034 (18.03%)	2017 (18%)	1988 (17.67%)	2034 (18.02%)
	History of stroke, TIA, or VTE	5668 (16.67%)	4748 (13.44%)	3780 (13.35%)	3724 (12.6%)	1716 (15.22%)	1663 (14.29%)	5188 (9.01%)	4925 (8.64%)	1715 (15.2%)	1611 (14.38%)	1599 (14.21%)	1716 (15.2%)
	Stroke or TIA	5648 (16.61%)	4729 (13.39%)	3759 (13.27%)	3705 (12.54%)	1712 (15.18%)	1657 (14.24%)	5165 (8.97%)	4898 (8.59%)	1711 (15.16%)	1609 (14.36%)	1596 (14.19%)	1712 (15.17%)
	VTE	24 (0.07%)	23 (0.07%)	24 (0.08%)	22 (0.07%)	4 (0.04%)	6 (0.05%)	24 (0.04%)	30 (0.05%)	4 (0.04%)	3 (0.03%)	4 (0.04%)	4 (0.04%)
	Vascular disease + peripheral vascular stenting	9819 (28.87%)	9129 (25.84%)	6894 (24.34%)	6696 (22.66%)	1607 (14.25%)	1438 (12.36%)	8584 (14.9%)	8783 (15.4%)	1606 (14.23%)	1507 (13.45%)	1407 (12.51%)	1607 (14.24%)
	Peripheral vascular stenting	240 (0.71%)	201 (0.57%)	180 (0.64%)	157 (0.53%)	44 (0.39%)	39 (0.34%)	247 (0.43%)	284 (0.5%)	44 (0.39%)	39 (0.35%)	32 (0.28%)	44 (0.39%)
	Anemia and coagulation defects	7013 (20.62%)	6828 (19.33%)	4445 (15.7%)	4570 (15.47%)	1039 (9.21%)	1012 (8.7%)	5089 (8.83%)	5261 (9.23%)	1039 (9.21%)	978 (8.73%)	1007 (8.95%)	1039 (9.21%)
	History of bleeding	9007 (26.49%)	8707 (24.65%)	5930 (20.94%)	6011 (20.34%)	1524 (13.51%)	1478 (12.7%)	6873 (11.93%)	7146 (12.53%)	1523 (13.5%)	1393 (12.43%)	1361 (12.1%)	1524 (13.5%)
	Thrombocytopenia	581 (1.71%)	515 (1.46%)	368 (1.3%)	365 (1.24%)	103 (0.91%)	100 (0.86%)	460 (0.8%)	452 (0.79%)	103 (0.91%)	85 (0.76%)	88 (0.78%)	103 (0.91%)
	Atherosclerotic disease	3097 (9.11%)	2916 (8.25%)	2031 (7.17%)	1979 (6.7%)	455 (4.03%)	411 (3.53%)	2402 (4.17%)	2520 (4.42%)	455 (4.03%)	431 (3.85%)	404 (3.59%)	455 (4.03%)
	Vascular disease	9819 (28.87%)	9129 (25.84%)	6891 (24.33%)	6696 (22.66%)	1607 (14.25%)	1438 (12.36%)	8584 (14.9%)	8780 (15.4%)	1606 (14.23%)	1507 (13.45%)	1407 (12.51%)	1607 (14.24%)
	Heart failure	13523 (39.77%)	14618 (41.38%)	10153 (35.85%)	10677 (36.13%)	2252 (19.97%)	2169 (18.64%)	12878 (22.35%)	13166 (23.09%)	2252 (19.96%)	2237 (19.97%)	2112 (18.78%)	2252 (19.95%)
	Dyspepsia or stomach discomfort	1183 (3.48%)	989 (2.8%)	871 (3.08%)	784 (2.65%)	258 (2.29%)	242 (2.08%)	1368 (2.37%)	1395 (2.45%)	258 (2.29%)	220 (1.96%)	229 (2.04%)	258 (2.29%)
	Coronary artery disease	8700 (25.58%)	8273 (23.42%)	6367 (22.48%)	6130 (20.75%)	1461 (12.95%)	1329 (11.42%)	8374 (14.54%)	8507 (14.92%)	1461 (12.95%)	1346 (12.01%)	1275 (11.33%)	1461 (12.95%)
	Obesity (ICD-10 claims)	4366 (12.84%)	3890 (11.01%)	3398 (12%)	3039 (10.28%)	871 (7.72%)	829 (7.13%)	4515 (7.84%)	4785 (8.39%)	871 (7.72%)	830 (7.41%)	793 (7.05%)	871 (7.72%)
	Liver disease	637 (1.87%)	522 (1.48%)	396 (1.4%)	392 (1.33%)	81 (0.72%)	97 (0.83%)	467 (0.81%)	463 (0.81%)	81 (0.72%)	80 (0.71%)	70 (0.62%)	81 (0.72%)
	Chronic kidney disease	7364 (21.65%)	8030 (22.73%)	3248 (11.47%)	3720 (12.59%)	515 (4.57%)	563 (4.84%)	3276 (5.69%)	3319 (5.82%)	515 (4.56%)	453 (4.04%)	460 (4.09%)	515 (4.56%)
	Maximum stage 1	148 (0.44%)	149 (0.42%)	90 (0.32%)	103 (0.35%)	14 (0.12%)	14 (0.12%)	80 (0.14%)	93 (0.16%)	14 (0.12%)	12 (0.11%)	15 (0.13%)	14 (0.12%)
	Maximum stage 2	565 (1.66%)	495 (1.4%)	391 (1.38%)	381 (1.29%)	78 (0.69%)	81 (0.7%)	420 (0.73%)	433 (0.76%)	78 (0.69%)	79 (0.71%)	71 (0.63%)	78 (0.69%)
	Maximum stage 3	3759 (11.05%)	3862 (10.93%)	1802 (6.36%)	2043 (6.91%)	281 (2.49%)	292 (2.51%)	1787 (3.1%)	1828 (3.21%)	281 (2.49%)	241 (2.15%)	251 (2.23%)	281 (2.49%)
	Maximum stage 4	1127 (3.31%)	1371 (3.88%)	283 (1%)	381 (1.29%)	38 (0.34%)	51 (0.44%)	312 (0.54%)	282 (0.49%)	38 (0.34%)	33 (0.29%)	34 (0.3%)	38 (0.34%)
	Other/unknown	1765 (5.19%)	2153 (6.09%)	682 (2.41%)	812 (2.75%)	104 (0.92%)	125 (1.07%)	677 (1.18%)	683 (1.2%)	104 (0.92%)	88 (0.79%)	89 (0.79%)	104 (0.92%)
	Chronic obstructive pulmonary disease	207 (0.61%)	207 (0.59%)	156 (0.55%)	163 (0.55%)	42 (0.37%)	38 (0.33%)	189 (0.33%)	192 (0.34%)	42 (0.37%)	32 (0.29%)	27 (0.24%)	42 (0.37%)
	Hospitalization with alcohol discharge code	858 (2.52%)	722 (2.04%)	649 (2.29%)	592 (2.00%)	181 (1.6%)	178 (1.53%)	874 (1.52%)	915 (1.6%)	181 (1.6%)	163 (1.45%)	164 (1.46%)	181 (1.6%)
**CHA**_**2**_ **DS**_**2**_ **-VASc score**	Mean (SD)	4.8 (1.3)	4.7 (1.3)	4.6 (1.2)	4.6 (1.2)	4.2 (1.3)	4.2 (1.2)	4.1 (1.2)	4.2 (1.2)	4.2 (1.3)	4.2 (1.3)	4.2 (1.2)	4.2 (1.3)
	2	871 (2.56%)	838 (2.37%)	897 (3.17%)	823 (2.79%)	617 (5.47%)	605 (5.2%)	3583 (6.22%)	3607 (6.33%)	610 (5.44%)	619 (5.49%)	644 (5.72%)	619 (5.48%)
	3	4324 (12.72%)	4952 (14.02%)	4252 (15.02%)	4696 (15.89%)	2649 (23.49%)	2952 (25.37%)	14041 (24.37%)	13553 (23.77%)	2693 (24.04%)	2653 (23.51%)	2741 (24.37%)	2653 (23.51%)
	≥4	28812 (84.72%)	29539 (83.61%)	23169 (81.82%)	24030 (81.32%)	8012 (71.04%)	8077 (69.43%)	39988 (69.41%)	39859 (69.9%)	7901 (70.52%)	8012 (71%)	7864 (69.91%)	8014 (71.01%)
**Concomitant treatment**	Antiplatelets*	16351 (48.08%)	15955 (45.16%)	13248 (46.78%)	13222 (44.75%)	4738 (42.01%)	4837 (41.58%)	24655 (42.79%)	24459 (42.9%)	4756 (42.45%)	4742 (42.02%)	4680 (41.6%)	4742 (42.02%)
	Aromatase inhibitors*	328 (0.96%)	278 (0.79%)	270 (0.95%)	233 (0.79%)	76 (0.67%)	80 (0.69%)	451 (0.78%)	450 (0.79%)	86 (0.77%)	76 (0.67%)	75 (0.67%)	76 (0.67%)
	NSAIDs*	2049 (6.03%)	1559 (4.41%)	1770 (6.25%)	1391 (4.71%)	807 (7.16%)	765 (6.58%)	4331 (7.52%)	4337 (7.61%)	769 (6.86%)	812 (7.2%)	822 (7.31%)	812 (7.19%)
	Corticosteroids*	4435 (13.04%)	3662 (10.37%)	3559 (12.57%)	3076 (10.41%)	1312 (11.63%)	1251 (10.75%)	6411 (11.13%)	6686 (11.73%)	1312 (11.63%)	1107 (9.88%)	1167 (10.37%)	1313 (11.63%)
	H2-receptor antagonists*	205 (0.6%)	157 (0.44%)	146 (0.52%)	133 (0.45%)	47 (0.42%)	48 (0.41%)	219 (0.38%)	226 (0.4%)	36 (0.32%)	47 (0.42%)	42 (0.37%)	47 (0.42%)
	Prostaglandins	571 (1.68%)	380 (1.08%)	593 (2.09%)	282 (0.95%)	203 (1.8%)	110 (0.95%)	1253 (2.17%)	1171 (2.05%)	172 (1.54%)	204 (1.81%)	220 (1.96%)	204 (1.81%)
	Proton pump inhibitors*	18628 (54.78%)	18232 (51.61%)	14734 (52.03%)	14570 (49.31%)	4955 (43.94%)	5000 (42.98%)	24098 (41.83%)	23966 (42.03%)	4865 (43.42%)	4956 (43.92%)	4850 (43.11%)	4956 (43.91%)
	Anticonvulsant strong inhibitor of hepatic enzymes*	296 (0.87%)	260 (0.74%)	221 (0.78%)	220 (0.74%)	68 (0.6%)	59 (0.51%)	314 (0.55%)	321 (0.56%)	46 (0.41%)	68 (0.6%)	64 (0.57%)	68 (0.6%)
	HIV protease inhibitors*	117 (0.34%)	54 (0.15%)	99 (0.35%)	33 (0.11%)	34 (0.3%)	11 (0.09%)	174 (0.3%)	191 (0.33%)	22 (0.2%)	34 (0.3%)	21 (0.19%)	34 (0.3%)
	Strong inhibitors of both CYP3A4 and P-gp*	450 (1.32%)	326 (0.92%)	385 (1.36%)	257 (0.87%)	167 (1.48%)	116 (1%)	707 (1.23%)	713 (1.25%)	110 (0.98%)	167 (1.48%)	119 (1.06%)	168 (1.49%)
	Statins*	5848 (17.2%)	5520 (15.62%)	4916 (17.36%)	4702 (15.91%)	1853 (16.43%)	1913 (16.44%)	9029 (15.67%)	9060 (15.89%)	1759 (15.7%)	1855 (16.44%)	1741 (15.48%)	1855 (16.44%)
	Selective estrogen receptor modulators*	75 (0.22%)	64 (0.18%)	65 (0.23%)	55 (0.19%)	23 (0.2%)	22 (0.19%)	117 (0.2%)	120 (0.21%)	25 (0.22%)	23 (0.2%)	29 (0.26%)	23 (0.2%)
	Serotonin reuptake inhibitors*	3924 (11.54%)	3456 (9.78%)	3069 (10.84%)	2834 (9.59%)	972 (8.62%)	1012 (8.7%)	4438 (7.7%)	4562 (8%)	900 (8.03%)	972 (8.61%)	908 (8.07%)	972 (8.61%)
	Sex hormones	881 (2.59%)	603 (1.71%)	835 (2.95%)	481 (1.63%)	308 (2.73%)	204 (1.75%)	1871 (3.25%)	1806 (3.17%)	305 (2.72%)	309 (2.74%)	908 (8.07%)	972 (8.61%)
	Erythropoiesis stimulating agents*	485 (1.43%)	554 (1.57%)	222 (0.78%)	264 (0.89%)	30 (0.27%)	35 (0.3%)	203 (0.35%)	232 (0.41%)	38 (0.34%)	30 (0.27%)	33 (0.29%)	30 (0.27%)
	Beta blockers*	19896 (58.51%)	21054 (59.59%)	16434 (58.03%)	17330 (58.65%)	6059 (53.72%)	6200 (53.29%)	31339 (54.4%)	31171 (54.67%)	6037 (53.88%)	6060 (53.7%)	5941 (52.81%)	6062 (53.71%)
	Antiarrhythmic agents*	15341 (45.11%)	16240 (45.97%)	13386 (47.27%)	14171 (47.96%)	6584 (58.38%)	6906 (59.36%)	34191 (59.35%)	33467 (58.69%)	6784 (60.55%)	6590 (58.4%)	6734 (59.86%)	6591 (58.4%)

*Variables that were adjusted for in the PS model. Scores (Charlson Comorbidity Index, HAS-BLED and CHA_2_DS_2_-VASc) were not included in the PS modelling as their components are singularly included, but were used as indicator for evaluating the fitness of the matching.

AIDS, acquired immunodeficiency syndrome; CKD, chronic kidney disease; CYP3A4, cytochrome P450 3A4; DOAC, direct oral anticoagulant; GIB, gastrointestinal bleed; HIV, human immunodeficiency virus; LTR, long-term recurrence; NSAID, nonsteroidal anti-inflammatory drug; P-gp, P-glycoprotein; PS, propensity score; SD, standard deviation; TIA, transient ischemic attack; VKA, vitamin K antagonist; VTE, venous thromboembolism.

**Table 2:** Baseline characteristics after PS matching for patients with HAS-BLED score ≥3.

		Pairwise comparison		Pairwise comparison		Pairwise comparison		Pairwise comparison		Pairwise comparison		Pairwise comparison	
Characteristic		Apixaban (n = 31,652)	VKAs (n = 31,652)	Rivaroxaban (n = 25,441)	VKAs (n = 25,441)	Dabigatran (n = 9,331)	VKAs (n = 9,331)	Apixaban (n = 46,845)	Rivaroxaban (n = 46,845)	Dabigatran (n = 9,315)	Rivaroxaban (n = 9,315)	Apixaban (n = 9,333)	Dabigatran (n = 9,333)
**Index dosage**	Standard dose	-	-	-	-	-	-	27619 (58.96%)	27387 (58.46%)	2988 (32.08%)	3003 (32.24%)	3030 (32.47%)	2988 (32.02%)
	Reduced dose	-	-	-	-	-	-	19226 (41.04%)	19458 (41.54%)	6327 (67.92%)	6312 (67.76%)	6303 (67.53%)	6345 (67.98%)
**Atrial fibrillation identification setting**	Inpatient claim with I48 code	25928 (81.92%)	26012 (82.18%)	20188 (79.35%)	20086 (78.95%)	6003 (64.33%)	5941 (63.67%)	29087 (62.09%)	29365 (62.69%)	5830 (62.59%)	5987 (64.27%)	5844 (62.62%)	6004 (64.33%)
	LTR registration with I48 code*	1022 (3.23%)	957 (3.02%)	1015 (3.99%)	935 (3.68%)	716 (7.67%)	692 (7.42%)	3754 (8.01%)	3695 (7.89%)	771 (8.28%)	717 (7.7%)	753 (8.07%)	717 (7.68%)
	Use of anti-arrhythmic drugs*	4702 (14.86%)	4683 (14.8%)	4238 (16.66%)	4420 (17.37%)	2612 (27.99%)	2698 (28.91%)	14004 (29.89%)	13785 (29.43%)	2714 (29.14%)	2611 (28.03%)	2736 (29.32%)	2612 (27.99%)
**Follow up time (months), censored at switch, discontinuation, interruption, death, pregnancy, dialysis, CKD stage V, or end of follow up, mean (SD)**		12.9 [12.2]	11.3 [11.6]	12.7 [12.3]	11.6 [11.8]	13.8 [12.7]	11.9 [12.2]	14.2 [12.9]	13.3 [12.7]	13.8 [12.7]	13.5 [12.8]	14.5 [13.0]	13.8 [12.7]
**Age at index date (years), mean (SD)**		81.5 (9.2)	81.8 (9.3)	80.2 (9.3)	80.2 (9.3)	78.9 (8.6)	79.1 (8.6)	78.6 (8.7)	78.5 (8.7)	78.9 (8.6)	78.8 (8.6)	79.1 (8.6)	78.9 (8.6)
**Age groups at index date**	18–54 years	257 (0.81%)	263 (0.83%)	252 (0.99%)	235 (0.92%)	69 (0.74%)	59 (0.63%)	340 (0.73%)	372 (0.79%)	66 (0.71%)	69 (0.74%)	60 (0.64%)	69 (0.74%)
	55–64 years*	972 (3.07%)	946 (2.99%)	924 (3.63%)	828 (3.25%)	263 (2.82%)	274 (2.94%)	1341 (2.86%)	1370 (2.92%)	262 (2.81%)	262 (2.81%)	258 (2.76%)	263 (2.82%)
	65–74 years*	5925 (18.72%)	5743 (18.14%)	5567 (21.88%)	5221 (20.52%)	2623 (28.11%)	2515 (26.95%)	14172 (30.25%)	14181 (30.27%)	2754 (29.57%)	2622 (28.15%)	2599 (27.85%)	2624 (28.12%)
	75–79 years*	4422 (13.97%)	4209 (13.3%)	3904 (15.35%)	3737 (14.69%)	1692 (18.13%)	1711 (18.34%)	8658 (18.48%)	8645 (18.45%)	1654 (17.76%)	1688 (18.12%)	1738 (18.62%)	1693 (18.14%)
	≥80 years*	20076 (63.43%)	20491 (64.74%)	14794 (58.15%)	15420 (60.61%)	4684 (50.20%)	4772 (51.14%)	22334 (47.68%)	22277 (47.56%)	4579 (49.16%)	4674 (50.18%)	4678 (50.12%)	4684 (50.19%)
**Sex**	Male	15887 (50.19%)	15945 (50.38%)	13171 (51.77%)	13080 (51.41%)	4972 (53.28%)	4928 (52.81%)	25822 (55.12%)	25826 (55.13%)	4956 (53.20%)	4966 (53.31%)	4957 (53.11%)	4973 (53.28%)
	Female*	15765 (49.81%)	15707 (49.62%)	12270 (48.23%)	12361 (48.59%)	4359 (46.72%)	4403 (47.19%)	21023 (44.88%)	21019 (44.87%)	4359 (46.8%)	4349 (46.69%)	4376 (46.89%)	4360 (46.72%)
**GIB risk factors**	Age ≥75 years	24498 (77.4%)	24700 (78.04%)	18698 (73.5%)	19157 (75.3%)	6376 (68.33%)	6483 (69.48%)	30992 (66.16%)	30922 (66.01%)	6233 (66.91%)	6362 (68.3%)	6416 (68.75%)	6377 (68.33%)
	HAS-BLED score, mean	3.6 (0.8)	3.6 (0.8)	3.5 (0.7)	3.5 (0.7)	3.3 (0.6)	3.3 (0.6)	3.3 (0.6)	3.3 (0.6)	3.3 (0.6)	3.3 (0.6)	3.3 (0.6)	3.3 (0.6)
	≥3*	31652 (100%)	31652 (100%)	25441 (100%)	25441 (100%)	9331 (100%)	9331 (100%)	46845 (100%)	46845 (100%)	9315 (100%)	9315 (100%)	9333 (100%)	9333 (100%)
	Prior medications (antiplatelets, NSAIDs, or corticosteroids)	22232 (70.24%)	22124 (69.9%)	18918 (74.36%)	18679 (73.42%)	7301 (78.24%)	7250 (77.7%)	38679 (82.57%)	38779 (82.78%)	7417 (79.62%)	7299 (78.36%)	7398 (79.27%)	7304 (78.26%)
	Renal impairment (CKD stage 3–4)	5531 (17.47%)	6084 (19.22%)	2518 (9.9%)	2917 (11.47%)	380 (4.07%)	418 (4.48%)	2518 (5.38%)	2563 (5.47%)	377 (4.05%)	380 (4.08%)	347 (3.72%)	380 (4.07%)
	Prior GI condition*	3351 (10.59%)	3170 (10.02%)	2546 (10.01%)	2419 (9.51%)	835 (8.95%)	813 (8.71%)	3759 (8.02%)	3849 (8.22%)	747 (8.02%)	835 (8.96%)	738 (7.91%)	835 (8.95%)
**Number of GIB risk factors**	1	1331 (4.21%)	1259 (3.98%)	1150 (4.52%)	1147 (4.51%)	503 (5.39%)	485 (5.2%)	1895 (4.05%)	1776 (3.79%)	427 (4.58%)	499 (5.36%)	443 (4.75%)	502 (5.38%)
	2	9497 (30%)	9276 (29.31%)	8276 (32.53%)	7923 (31.14%)	3345 (35.85%)	3270 (35.04%)	16857 (35.98%)	16943 (36.17%)	3469 (37.24%)	3337 (35.82%)	3371 (36.12%)	3347 (35.86%)
	3	16637 (52.56%)	16811 (53.11%)	13767 (54.11%)	13973 (54.92%)	4927 (52.8%)	5050 (54.12%)	25305 (54.02%)	25337 (54.09%)	4971 (53.37%)	4923 (52.85%)	5046 (54.07%)	4928 (52.8%)
	4	3907 (12.34%)	4044 (12.78%)	2122 (8.34%)	2289 (9%)	531 (5.69%)	510 (5.47%)	2671 (5.7%)	2660 (5.68%)	429 (4.61%)	531 (5.7%)	456 (4.89%)	531 (5.69%)
	5	280 (0.88%)	262 (0.83%)	126 (0.5%)	109 (0.43%)	25 (0.27%)	16 (0.17%)	117 (0.25%)	129 (0.28%)	19 (0.2%)	25 (0.27%)	17 (0.18%)	25 (0.27%)
**Charlson Comorbidity Index score**	Mean (SD)	3.1 [2.4]	3.1 [2.4]	2.7 [2.2]	2.7 [2.2]	2.1 [2.1]	2.1 [2.1]	1.9 [2.0]	2.0 [2.1]	2.1 [2.1]	2.0 [2.1]	2.0 [2.0]	2.1 [2.1]
	0	2928 (9.25%)	3199 (10.11%)	3024 (11.89%)	3178 (12.49%)	2163 (23.18%)	2098 (22.48%)	11570 (24.7%)	11532 (24.62%)	2265 (24.32%)	2164 (23.23%)	2317 (24.83%)	2164 (23.19%)
	1 or 2	11853 (37.45%)	11307 (35.72%)	11169 (43.9%)	10580 (41.59%)	4040 (43.3%)	4027 (43.16%)	21600 (46.11%)	21314 (45.5%)	4044 (43.41%)	4039 (43.36%)	4052 (43.42%)	4041 (43.3%)
	3 or 4	9950 (31.44%)	10130 (32%)	7163 (28.16%)	7482 (29.41%)	2125 (22.77%)	2196 (23.53%)	9207 (19.65%)	9354 (19.97%)	2039 (21.89%)	2109 (22.64%)	2054 (22.01%)	2125 (22.77%)
	≥5	6921 (21.87%)	7016 (22.17%)	4085 (16.06%)	4201 (16.51%)	1003 (10.75%)	1010 (10.82%)	4468 (9.54%)	4645 (9.92%)	967 (10.38%)	1003 (10.77%)	910 (9.75%)	1003 (10.75%)
**Comorbidities**	Myocardial infarction*	4230 (13.36%)	4338 (13.71%)	3316 (13.03%)	3305 (12.99%)	705 (7.56%)	685 (7.34%)	4540 (9.69%)	4614 (9.85%)	684 (7.34%)	705 (7.57%)	674 (7.22%)	705 (7.55%)
	Congestive heart failure*	15434 (48.76%)	16128 (50.95%)	11304 (44.43%)	11539 (45.36%)	2504 (26.84%)	2479 (26.57%)	14330 (30.59%)	14640 (31.25%)	2500 (26.84%)	2503 (26.87%)	2391 (25.62%)	2504 (26.83%)
	Peripheral vascular disease*	5129 (16.2%)	5088 (16.07%)	3724 (14.64%)	3668 (14.42%)	930 (9.97%)	937 (10.04%)	4789 (10.22%)	4996 (10.66%)	871 (9.35%)	930 (9.98%)	849 (9.1%)	930 (9.96%)
	Cerebrovascular disease*	8389 (26.5%)	7625 (24.09%)	6166 (24.24%)	6011 (23.63%)	2786 (29.86%)	2800 (30.01%)	8614 (18.39%)	8562 (18.28%)	2728 (29.29%)	2768 (29.72%)	2691 (28.83%)	2786 (29.85%)
	Dementia*	3630 (11.47%)	3562 (11.25%)	2566 (10.09%)	2576 (10.13%)	537 (5.76%)	520 (5.57%)	3191 (6.81%)	3224 (6.88%)	480 (5.15%)	537 (5.76%)	496 (5.31%)	537 (5.75%)
	Chronic pulmonary disease*	8100 (25.59%)	7578 (23.94%)	6300 (24.76%)	5974 (23.48%)	1920 (20.58%)	1922 (20.6%)	10081 (21.52%)	10347 (22.09%)	1845 (19.81%)	1920 (20.61%)	1841 (19.73%)	1921 (20.58%)
	Connective tissue disease*	664 (2.1%)	622 (1.97%)	470 (1.85%)	465 (1.83%)	122 (1.31%)	126 (1.35%)	614 (1.31%)	643 (1.37%)	108 (1.16%)	122 (1.31%)	108 (1.16%)	122 (1.31%)
	Ulcer disease*	627 (1.98%)	611 (1.93%)	394 (1.55%)	414 (1.63%)	121 (1.3%)	128 (1.37%)	454 (0.97%)	474 (1.01%)	94 (1.01%)	121 (1.3%)	118 (1.26%)	121 (1.3%)
	Mild liver disease*	1172 (3.7%)	1130 (3.57%)	909 (3.57%)	903 (3.55%)	206 (2.21%)	209 (2.24%)	1109 (2.37%)	1168 (2.49%)	208 (2.23%)	206 (2.21%)	172 (1.84%)	206 (2.21%)
	Diabetes*	9033 (28.54%)	9032 (28.54%)	7037 (27.66%)	6990 (27.48%)	2196 (23.53%)	2204 (23.62%)	12118 (25.87%)	12180 (26%)	2188 (23.49%)	2193 (23.54%)	2164 (23.19%)	2196 (23.53%)
	Diabetes with end-organ damage*	1737 (5.49%)	1905 (6.02%)	1032 (4.06%)	1116 (4.39%)	216 (2.31%)	233 (2.5%)	1126 (2.4%)	1145 (2.44%)	220 (2.36%)	216 (2.32%)	211 (2.26%)	216 (2.31%)
	Hemiplegia*	3582 (11.32%)	3204 (10.12%)	2478 (9.74%)	2512 (9.87%)	1314 (14.08%)	1356 (14.53%)	3212 (6.86%)	3115 (6.65%)	1251 (13.43%)	1298 (13.93%)	1245 (13.34%)	1313 (14.07%)
	Moderate or severe renal disease	8943 (28.25%)	10088 (31.87%)	4312 (16.95%)	5103 (20.06%)	704 (7.54%)	831 (8.91%)	4397 (9.39%)	4493 (9.59%)	709 (7.61%)	704 (7.56%)	669 (7.17%)	704 (7.54%)
	Any tumor (except for malignant neoplasm of skin)*	3635 (11.48%)	3438 (10.86%)	2843 (11.17%)	2667 (10.48%)	867 (9.29%)	827 (8.86%)	3928 (8.39%)	4105 (8.76%)	812 (8.72%)	867 (9.31%)	795 (8.52%)	868 (9.3%)
	Metastatic solid tumor*	688 (2.17%)	646 (2.04%)	609 (2.39%)	538 (2.11%)	166 (1.78%)	174 (1.86%)	793 (1.69%)	822 (1.75%)	155 (1.66%)	166 (1.78%)	152 (1.63%)	166 (1.78%)
	HIV/ AIDS*	47 (0.15%)	48 (0.15%)	43 (0.17%)	41 (0.16%)	8 (0.09%)	7 (0.08%)	44 (0.09%)	49 (0.1%)	9 (0.1%)	8 (0.09%)	12 (0.13%)	8 (0.09%)
	Moderate or severe liver disease*	311 (0.98%)	317 (1%)	209 (0.82%)	227 (0.89%)	53 (0.57%)	55 (0.59%)	214 (0.46%)	220 (0.47%)	48 (0.52%)	53 (0.57%)	38 (0.41%)	53 (0.57%)
	Hypertension	30727 (97.08%)	30801 (97.31%)	24651 (96.89%)	24715 (97.15%)	9040 (96.88%)	9058 (97.07%)	45622 (97.39%)	45620 (97.38%)	9024 (96.88%)	9046 (97.11%)	9099 (97.49%)	9042 (96.88%)
	Diabetes mellitus	9949 (31.43%)	10006 (31.61%)	7697 (30.25%)	7731 (30.39%)	2409 (25.82%)	2432 (26.06%)	13009 (27.77%)	13139 (28.05%)	2405 (25.82%)	2398 (25.74%)	2347 (25.15%)	2409 (25.81%)
	History of stroke, TIA, or VTE	6419 (20.28%)	5671 (17.92%)	4445 (17.47%)	4542 (17.85%)	2285 (24.49%)	2220 (23.79%)	6769 (14.45%)	6184 (13.2%)	2267 (24.34%)	2041 (21.91%)	2123 (22.75%)	2284 (24.47%)
	Stroke or TIA	6398 (20.21%)	5656 (17.87%)	4430 (17.41%)	4528 (17.8%)	2282 (24.46%)	2217 (23.76%)	6740 (14.39%)	6159 (13.15%)	2264 (24.3%)	2039 (21.89%)	2119 (22.7%)	2281 (24.44%)
	VTE	22 (0.07%)	21 (0.07%)	20 (0.08%)	20 (0.08%)	4 (0.04%)	4 (0.04%)	32 (0.07%)	32 (0.07%)	4 (0.04%)	3 (0.03%)	4 (0.04%)	4 (0.04%)
	Vascular disease + peripheral vascular stenting	10652 (33.65%)	10461 (33.05%)	7849 (30.85%)	7752 (30.47%)	1930 (20.68%)	1917 (20.54%)	10418 (22.24%)	10689 (22.82%)	1928 (20.7%)	1865 (20.02%)	1845 (19.77%)	1930 (20.68%)
	Peripheral vascular stenting	281 (0.89%)	258 (0.82%)	252 (0.99%)	210 (0.83%)	63 (0.68%)	68 (0.73%)	371 (0.79%)	415 (0.89%)	63 (0.68%)	51 (0.55%)	54 (0.58%)	63 (0.68%)
	Anemia and coagulation defects	8234 (26.01%)	8354 (26.39%)	5631 (22.13%)	5815 (22.86%)	1320 (14.15%)	1416 (15.18%)	6570 (14.02%)	6828 (14.58%)	1320 (14.17%)	1353 (14.52%)	1304 (13.97%)	1320 (14.14%)
	History of bleeding	10807 (34.14%)	10804 (34.13%)	7678 (30.18%)	7766 (30.53%)	2068 (22.16%)	2063 (22.11%)	9266 (19.78%)	9610 (20.51%)	2064 (22.16%)	1959 (21.03%)	1921 (20.58%)	2068 (22.16%)
	Thrombocytopenia	680 (2.15%)	688 (2.17%)	488 (1.92%)	502 (1.97%)	125 (1.34%)	130 (1.39%)	569 (1.21%)	578 (1.23%)	124 (1.33%)	107 (1.15%)	106 (1.14%)	125 (1.34%)
	Atherosclerotic disease	3663 (11.57%)	3561 (11.25%)	2581 (10.15%)	2471 (9.71%)	597 (6.4%)	608 (6.52%)	3319 (7.09%)	3398 (7.25%)	597 (6.41%)	588 (6.31%)	579 (6.2%)	597 (6.4%)
	Vascular disease	10651 (33.65%)	10459 (33.04%)	7847 (30.84%)	7750 (30.46%)	1930 (20.68%)	1916 (20.53%)	10417 (22.24%)	10685 (22.81%)	1928 (20.7%)	1864 (20.01%)	1845 (19.77%)	1930 (20.68%)
	Heart failure	13132 (41.49%)	13990 (44.2%)	9344 (36.73%)	9802 (38.53%)	1974 (21.16%)	2041 (21.87%)	11732 (25.04%)	11918 (25.44%)	1973 (21.18%)	2060 (22.11%)	1960 (21%)	1974 (21.15%)
	Dyspepsia or stomach discomfort	1086 (3.43%)	1019 (3.22%)	869 (3.42%)	790 (3.11%)	239 (2.56%)	222 (2.38%)	1269 (2.71%)	1345 (2.87%)	239 (2.57%)	226 (2.43%)	239 (2.56%)	239 (2.56%)
	Coronary artery disease	10160 (32.1%)	10262 (32.42%)	7913 (31.1%)	7850 (30.86%)	1927 (20.65%)	1938 (20.77%)	11183 (23.87%)	11386 (24.31%)	1924 (20.65%)	1895 (20.34%)	1844 (19.76%)	1927 (20.65%)
	Obesity (ICD-10 claims)	5508 (17.4%)	5329 (16.84%)	4495 (17.67%)	4163 (16.36%)	1257 (13.47%)	1218 (13.05%)	6200 (13.24%)	6383 (13.63%)	1253 (13.45%)	1183 (12.7%)	1179 (12.63%)	1256 (13.46%)
	Liver disease	1264 (3.99%)	1195 (3.78%)	975 (3.83%)	956 (3.76%)	214 (2.29%)	223 (2.39%)	1176 (2.51%)	1241 (2.65%)	214 (2.3%)	222 (2.38%)	187 (2%)	214 (2.29%)
	Chronic kidney disease	8458 (26.72%)	9431 (29.8%)	4007 (15.75%)	4556 (17.91%)	647 (6.93%)	715 (7.66%)	4021 (8.58%)	4132 (8.82%)	647 (6.95%)	635 (6.82%)	603 (6.46%)	647 (6.93%)
	Maximum stage 1	189 (0.6%)	184 (0.58%)	109 (0.43%)	120 (0.47%)	23 (0.25%)	20 (0.21%)	115 (0.25%)	120 (0.26%)	23 (0.25%)	25 (0.27%)	22 (0.24%)	23 (0.25%)
	Maximum stage 2	612 (1.93%)	597 (1.89%)	472 (1.86%)	487 (1.91%)	104 (1.11%)	98 (1.05%)	513 (1.1%)	534 (1.14%)	104 (1.12%)	100 (1.07%)	97 (1.04%)	104 (1.11%)
	Maximum stage 3	4253 (13.44%)	4511 (14.25%)	2179 (8.56%)	2485 (9.77%)	333 (3.57%)	362 (3.88%)	2178 (4.65%)	2224 (4.75%)	333 (3.57%)	331 (3.55%)	308 (3.3%)	333 (3.57%)
	Maximum stage 4	1278 (4.04%)	1573 (4.97%)	339 (1.33%)	432 (1.7%)	47 (0.5%)	56 (0.6%)	340 (0.73%)	339 (0.72%)	47 (0.5%)	46 (0.49%)	39 (0.42%)	47 (0.5%)
	Other/unknown	2126 (6.72%)	2566 (8.11%)	908 (3.57%)	1032 (4.06%)	140 (1.5%)	179 (1.92%)	875 (1.87%)	915 (1.95%)	140 (1.5%)	133 (1.43%)	137 (1.47%)	140 (1.5%)
	Chronic obstructive pulmonary disease	194 (0.61%)	198 (0.63%)	147 (0.58%)	147 (0.58%)	37 (0.4%)	42 (0.45%)	157 (0.34%)	181 (0.39%)	37 (0.4%)	43 (0.46%)	33 (0.35%)	37 (0.4%)
	Hospitalization with alcohol discharge code	1962 (6.20%)	1861 (5.88%)	1780 (7%)	1620 (6.37%)	524 (5.62%)	512 (5.49%)	2663 (5.68%)	2806 (5.99%)	523 (5.61%)	535 (5.74%)	477 (5.11%)	524 (5.61%)
**CHA**_**2**_ **DS**_**2**_ **-VASc score**	Mean (SD)	4.7 (1.4)	4.7 (1.4)	4.5 (1.4)	4.6 (1.3)	4.3 (1.4)	4.3 (1.4)	4.1 (1.3)	4.1 (1.3)	4.2 (1.4)	4.3 (1.4)	4.3 (1.4)	4.3 (1.4)
	0	7 (0.02%)	6 (0.02%)	8 (0.03%)	6 (0.02%)	3 (0.03%)	1 (0.01%)	11 (0.02%)	18 (0.04%)	2 (0.02%)	3 (0.03%)	1 (0.01%)	3 (0.03%)
	1	159 (0.5%)	159 (0.5%)	154 (0.61%)	147 (0.58%)	49 (0.53%)	55 (0.59%)	308 (0.66%)	320 (0.68%)	47 (0.5%)	49 (0.53%)	47 (0.5%)	49 (0.53%)
	2	1183 (3.74%)	1065 (3.36%)	1205 (4.74%)	1013 (3.98%)	666 (7.14%)	567 (6.08%)	4085 (8.72%)	4159 (8.88%)	783 (8.41%)	668 (7.17%)	712 (7.63%)	668 (7.16%)
	3	4231 (13.37%)	4344 (13.72%)	4109 (16.15%)	3999 (15.72%)	1969 (21.1%)	2063 (22.11%)	10952 (23.38%)	11110 (23.72%)	2045 (21.95%)	1969 (21.14%)	2054 (22.01%)	1969 (21.1%)
	≥4	26072 (82.37%)	26078 (82.39%)	19965 (78.48%)	20276 (79.7%)	6644 (71.2%)	6645 (71.21%)	31489 (67.22%)	31238 (66.68%)	6438 (69.11%)	6626 (71.13%)	6519 (69.85%)	6644 (71.19%)
**Concomitant treatment**	Antiplatelets*	19747 (62.39%)	20006 (63.21%)	16854 (66.25%)	16930 (66.55%)	6399 (68.58%)	6444 (69.06%)	34352 (73.33%)	34192 (72.99%)	6570 (70.53%)	6396 (68.66%)	6519 (69.85%)	6401 (68.58%)
	Aromatase inhibitors*	253 (0.8%)	217 (0.69%)	203 (0.8%)	173 (0.68%)	61 (0.65%)	55 (0.59%)	298 (0.64%)	337 (0.72%)	69 (0.74%)	61 (0.65%)	60 (0.64%)	61 (0.65%)
	NSAIDs*	2620 (8.28%)	2059 (6.51%)	2492 (9.8%)	1888 (7.42%)	1184 (12.69%)	1064 (11.4%)	6207 (13.25%)	6317 (13.48%)	1112 (11.94%)	1186 (12.73%)	1156 (12.39%)	1187 (12.72%)
	Corticosteroids*	4148 (13.11%)	3564 (11.26%)	3328 (13.08%)	2912 (11.45%)	1169 (12.53%)	1102 (11.81%)	5837 (12.46%)	5990 (12.79%)	1171 (12.57%)	1045 (11.22%)	1113 (11.93%)	1172 (12.56%)
	H2-receptor antagonists*	163 (0.51%)	160 (0.51%)	141 (0.55%)	133 (0.52%)	34 (0.36%)	28 (0.3%)	195 (0.42%)	202 (0.43%)	28 (0.3%)	34 (0.37%)	26 (0.28%)	34 (0.36%)
	Prostaglandins	717 (2.27%)	544 (1.72%)	768 (3.02%)	443 (1.74%)	287 (3.08%)	175 (1.88%)	1731 (3.7%)	1578 (3.37%)	263 (2.82%)	290 (3.11%)	321 (3.44%)	290 (3.11%)
	Proton pump inhibitors*	18529 (58.54%)	18575 (58.69%)	14604 (57.4%)	14531 (57.12%)	4818 (51.63%)	4902 (52.53%)	23710 (50.61%)	23922 (51.07%)	4758 (51.08%)	4810 (51.64%)	4743 (50.82%)	4819 (51.63%)
	Anticonvulsant strong inhibitor of hepatic enzymes*	267 (0.84%)	259 (0.82%)	210 (0.83%)	195 (0.77%)	60 (0.64%)	70 (0.75%)	284 (0.61%)	287 (0.61%)	62 (0.67%)	60 (0.64%)	55 (0.59%)	60 (0.64%)
	HIV protease inhibitors*	106 (0.33%)	102 (0.32%)	109 (0.43%)	84 (0.33%)	43 (0.46%)	38 (0.41%)	243 (0.52%)	226 (0.48%)	37 (0.4%)	43 (0.46%)	37 (0.4%)	43 (0.46%)
	Strong inhibitors of both CYP3A4 and P-gp*	483 (1.53%)	405 (1.28%)	421 (1.65%)	332 (1.3%)	180 (1.93%)	163 (1.75%)	818 (1.75%)	825 (1.76%)	162 (1.74%)	182 (1.95%)	181 (1.94%)	183 (1.96%)
	Statins*	6138 (19.39%)	5799 (18.32%)	4924 (19.35%)	4836 (19.01%)	1955 (20.95%)	1942 (20.81%)	9721 (20.75%)	9851 (21.03%)	1877 (20.15%)	1950 (20.93%)	1970 (21.11%)	1956 (20.96%)
	Selective estrogen receptor modulators*	52 (0.16%)	41 (0.13%)	45 (0.18%)	33 (0.13%)	17 (0.18%)	14 (0.15%)	77 (0.16%)	79 (0.17%)	15 (0.16%)	17 (0.18%)	21 (0.23%)	17 (0.18%)
	Selective serotonin reuptake inhibitors*	3314 (10.47%)	3111 (9.83%)	2554 (10.04%)	2400 (9.43%)	857 (9.18%)	885 (9.48%)	3619 (7.73%)	3752 (8.01%)	828 (8.89%)	854 (9.17%)	849 (9.1%)	857 (9.18%)
	Hormones	969 (3.06%)	726 (2.29%)	980 (3.85%)	596 (2.34%)	365 (3.91%)	247 (2.65%)	2173 (4.64%)	2020 (4.31%)	344 (3.69%)	368 (3.95%)	413 (4.43%)	368 (3.94%)
	Erythropoesis stimulating agents*	540 (1.71%)	597 (1.89%)	238 (0.94%)	269 (1.06%)	36 (0.39%)	34 (0.36%)	243 (0.52%)	251 (0.54%)	33 (0.35%)	36 (0.39%)	41 (0.44%)	36 (0.39%)
	Beta blockers*	20343 (64.27%)	20667 (65.29%)	16321 (64.15%)	16523 (64.95%)	5684 (60.92%)	5725 (61.35%)	29419 (62.8%)	29333 (62.62%)	5627 (60.41%)	5675 (60.92%)	5620 (60.22%)	5684 (60.9%)
	Antiarrhythmic agents*	14165 (44.75%)	14315 (45.23%)	11935 (46.91%)	12053 (47.38%)	5153 (55.22%)	5200 (55.73%)	27081 (57.81%)	26945 (57.52%)	5268 (56.55%)	5149 (55.28%)	5224 (55.97%)	5154 (55.22%)

*Variables that were adjusted for in the PS model. Scores (Charlson Comorbidity Index, HAS-BLED and CHA_2_DS_2_-VASc) were not included in the PS modelling as their components are singularly included, but were used as indicator for evaluating the fitness of the matching.

AIDS, acquired immunodeficiency syndrome; CKD, chronic kidney disease; CYP3A4, cytochrome P450 3A4; DOAC, direct oral anticoagulant; GIB, gastrointestinal bleed; HIV, human immunodeficiency virus; LTR, long-term recurrence; NSAID, nonsteroidal anti-inflammatory drug; P-gp, P-glycoprotein; PS, propensity score; SD, standard deviation; TIA, transient ischemic attack; VKA, vitamin K antagonist; VTE, venous thromboembolism.

**Table 3:** Baseline characteristics after PS matching for patients receiving concomitant medication.

		Pairwise comparison		Pairwise comparison		Pairwise comparison		Pairwise comparison		Pairwise comparison		Pairwise comparison	
Characteristic		Apixaban (n = 26,155)	VKAs (n = 26,155)	Rivaroxaban (n = 22,619)	VKAs (n = 22,619)	Dabigatran (n = 9,946)	VKAs (n = 9,946)	Apixaban (n = 57,573)	Rivaroxaban (n = 57,573)	Rivaroxaban (n = 10,141)	Dabigatran (n = 10,141)	Apixaban (n = 10,145)	Dabigatran (n = 10,145)
**Index dosage**	Standard dose	-	-	-	-	-	-	37931 (65.88%)	37637 (65.37%)	4037 (39.81%)	4013 (39.57%)	4102 (40.43%)	4013 (39.56%)
	Reduced dose	-	-	-	-	-	-	19642 (34.12%)	19936 (34.63%)	6104 (60.19%)	6128 (60.43%)	6043 (59.57%)	6132 (60.44%)
**Atrial fibrillation identification setting**	Inpatient claim with I48 code	19785 (75.65%)	19613 (74.99%)	16504 (72.97%)	16277 (71.96%)	5444 (54.74%)	5441 (54.71%)	30951 (53.76%)	31226 (54.24%)	5166 (50.94%)	5455 (53.79%)	5317 (52.41%)	5459 (53.81%)
	LTR registration with I48 code*	1315 (5.03%)	1167 (4.46%)	1251 (5.53%)	1152 (5.09%)	1047 (10.53%)	955 (9.6%)	6342 (11.02%)	6236 (10.83%)	1196 (11.79%)	1111 (10.96%)	1145 (11.29%)	1111 (10.95%)
	Use of anti-arrhythmic drugs*	5055 (19.33%)	5375 (20.55%)	4864 (21.5%)	5190 (22.95%)	3455 (34.74%)	3550 (35.69%)	20280 (35.22%)	20111 (34.93%)	3779 (37.26%)	3575 (35.25%)	3683 (36.3%)	3575 (35.24%)
**Follow up time (months), censored at switch, discontinuation, interruption, death, pregnancy, dialysis, CKD stage V, or end of follow up, mean (SD)**		13.0 [12.4]	11.2 [11.8]	12.7 [12.4]	11.4 [12]	13.7 [12.9]	11.2 [12.1]	13.9 [13.0]	13.1 [12.8]	13.3 [13]	13.7 [12.9]	14.2 [13.2]	13.7 [12.9]
**Age at index date (years), mean (SD)**		79.1 (11.0)	79.5 (11.0)	77.9 (11.2)	78.6 (11.1)	75.8 (10.8)	76.3 (10.9)	74.5 (11.2)	74.2 (11.4)	75.6 (10.8)	75.7 (10.8)	75.8 (10.7)	75.7 (10.8)
**Age groups at index date**	18–54 years	789 (3.02%)	680 (2.6%)	759 (3.36%)	666 (2.94%)	346 (3.48%)	347 (3.49%)	2823 (4.9%)	3109 (5.4%)	345 (3.4%)	351 (3.46%)	357 (3.52%)	351 (3.46%)
	55–64 years*	2086 (7.98%)	1961 (7.5%)	2080 (9.2%)	1877 (8.3%)	1067 (10.73%)	1023 (10.29%)	7430 (12.91%)	7546 (13.11%)	1147 (11.31%)	1111 (10.96%)	1122 (11.06%)	1111 (10.95%)
	65–74 years*	5081 (19.43%)	5017 (19.18%)	4857 (21.47%)	4720 (20.87%)	2770 (27.85%)	2621 (26.35%)	17096 (29.69%)	16922 (29.39%)	2905 (28.65%)	2882 (28.42%)	2932 (28.9%)	2883 (28.42%)
	75–79 years*	3656 (13.98%)	3420 (13.08%)	3295 (14.57%)	3122 (13.8%)	1582 (15.91%)	1590 (15.99%)	9127 (15.85%)	9132 (15.86%)	1659 (16.36%)	1608 (15.86%)	1567 (15.45%)	1610 (15.87%)
	≥80 years*	14543 (55.60%)	15077 (57.64%)	11628 (51.41%)	12234 (54.09%)	4181 (42.04%)	4365 (43.89%)	21097 (36.64%)	20864 (36.24%)	4085 (40.28%)	4189 (41.31%)	4167 (41.07%)	4190 (41.30%)
**Sex**	Male	14160 (54.14%)	14284 (54.61%)	12506 (55.29%)	12537 (55.43%)	5644 (56.75%)	5584 (56.14%)	33994 (59.04%)	34113 (59.25%)	5847 (57.66%)	5773 (56.93%)	5741 (56.59%)	5775 (56.92%)
	Female*	11995 (45.86%)	11871 (45.39%)	10113 (44.71%)	10082 (44.57%)	4302 (43.25%)	4362 (43.86%)	23579 (40.95%)	23460 (40.75%)	4294 (42.34%)	4368 (43.07%)	4404 (43.41%)	4370 (43.08%)
**GIB risk factors**	Age ≥75 years	18199 (69.58%)	18497 (70.72%)	14923 (65.98%)	15356 (67.89%)	5763 (57.94%)	5955 (59.87%)	30224 (52.5%)	29996 (52.1%)	5744 (56.64%)	5797 (57.16%)	5734 (56.52%)	5800 (57.17%)
	HAS-BLED score, mean (SD)	3.3 (1.1)	3.4 (1.0)	3.2 (1.0)	3.2 (1.0)	2.9 (0.9)	2.9 (0.9)	2.8 (0.9)	2.8 (1.0)	2.9 (0.9)	2.9 (1.0)	2.9 (0.9)	2.9 (1.0)
	0	123 (0.47%)	123 (0.47%)	138 (0.61%)	81 (0.36%)	83 (0.83%)	68 (0.68%)	711 (1.23%)	792 (1.38%)	100 (0.99%)	85 (0.84%)	102 (1.01%)	85 (0.84%)
	1	879 (3.36%)	879 (3.36%)	883 (3.9%)	708 (3.13%)	582 (5.85%)	531 (5.34%)	4191 (7.28%)	4551 (7.9%)	633 (6.24%)	615 (6.06%)	633 (6.24%)	615 (6.06%)
	2	3729 (14.26%)	3729 (14.26%)	3606 (15.94%)	3236 (14.31%)	2053 (20.64%)	1995 (20.06%)	13525 (23.49%)	13450 (23.36%)	2099 (20.7%)	2140 (21.1%)	2149 (21.18%)	2141 (21.1%)
	≥3*	21424 (81.91%)	22117 (84.56%)	17992 (79.54%)	18594 (82.21%)	7228 (72.67%)	7352 (73.92%)	39146 (67.99%)	38780 (67.36%)	7309 (72.07%)	7301 (71.99%)	7261 (71.57%)	7304 (72%)
	Prior medications (antiplatelets, NSAIDs, or corticosteroids)	26155 (100%)	26155 (100%)	22619 (100%)	22619 (100%)	9946 (100%)	9946 (100%)	57573 (100%)	57573 (100%)	10141 (100%)	10141 (100%)	10145 (100%)	10145 (100%)
	Renal impairment (CKD stage 3–4)	3226 (12.33%)	3598 (13.76%)	1460 (6.45%)	1822 (8.06%)	228 (2.29%)	263 (2.64%)	1510 (2.62%)	1511 (2.62%)	234 (2.31%)	228 (2.25%)	198 (1.95%)	228 (2.25%)
	Prior GI condition*	2174 (8.31%)	1957 (7.48%)	1750 (7.74%)	1582 (6.99%)	689 (6.93%)	682 (6.86%)	3466 (6.02%)	3522 (6.12%)	647 (6.38%)	693 (6.83%)	596 (5.87%)	697 (6.87%)
**Number of GIB risk factors**	1	2818 (10.77%)	2349 (8.98%)	2781 (12.29%)	2349 (10.39%)	1794 (18.04%)	1619 (16.28%)	12958 (22.51%)	13351 (23.19%)	1920 (18.93%)	1896 (18.7%)	1965 (19.37%)	1896 (18.69%)
	2	5930 (22.67%)	5742 (21.95%)	5761 (25.47%)	5554 (24.55%)	2957 (29.73%)	2977 (29.93%)	17678 (30.71%)	17461 (30.33%)	3020 (29.78%)	3031 (29.89%)	3013 (29.7%)	3031 (29.88%)
	3	13405 (51.25%)	14038 (53.67%)	11991 (53.01%)	12452 (55.05%)	4659 (46.84%)	4791 (48.17%)	24257 (42.13%)	24064 (41.8%)	4711 (46.45%)	4679 (46.14%)	4740 (46.72%)	4681 (46.14%)
	4	3725 (14.24%)	3753 (14.35%)	1962 (8.67%)	2160 (9.55%)	511 (5.14%)	543 (5.46%)	2566 (4.46%)	2568 (4.46%)	468 (4.61%)	510 (5.03%)	412 (4.06%)	512 (5.05%)
	5	277 (1.06%)	273 (1.04%)	124 (0.55%)	104 (0.46%)	25 (0.25%)	16 (0.16%)	114 (0.2%)	129 (0.22%)	22 (0.22%)	25 (0.25%)	15 (0.15%)	25 (0.25%)
**Charlson Comorbidity Index score**	Mean (SD)	2.7 [2.3]	2.7 [2.4]	2.3 [2.2]	2.4 [2.2]	1.6 [1.9]	1.7 [1.9]	1.5 [1.8]	1.5 [1.9]	1.5 [1.9]	1.6 [1.9]	1.5 [1.9]	1.6 [1.9]
	0	3767 (14.4%)	3990 (15.26%)	3699 (16.35%)	3973 (17.56%)	3246 (32.64%)	3056 (30.73%)	19937 (34.63%)	20101 (34.91%)	3661 (36.1%)	3401 (33.54%)	3622 (35.7%)	3401 (33.52%)
	1 or 2	10977 (41.97%)	10629 (40.64%)	10800 (47.75%)	10248 (45.31%)	4508 (45.32%)	4584 (46.09%)	26566 (46.14%)	26099 (45.33%)	4367 (43.06%)	4540 (44.77%)	4500 (44.36%)	4542 (44.77%)
	3 or 4	6814 (26.05%)	6849 (26.19%)	5233 (23.14%)	5404 (23.89%)	1484 (14.92%)	1558 (15.66%)	7536 (13.09%)	7794 (13.54%)	1456 (14.36%)	1494 (14.73%)	1403 (13.83%)	1494 (14.73%)
	≥5	4597 (17.58%)	4687 (17.92%)	2887 (12.76%)	2994 (13.24%)	708 (7.12%)	748 (7.52%)	3534 (6.14%)	3579 (6.22%)	657 (6.48%)	706 (6.96%)	620 (6.11%)	708 (6.98%)
**Comorbidities**	Myocardial infarction*	4311 (16.48%)	4354 (16.65%)	3527 (15.59%)	3553 (15.71%)	777 (7.81%)	825 (8.29%)	5509 (9.57%)	5596 (9.72%)	765 (7.54%)	778 (7.67%)	737 (7.26%)	778 (7.67%)
	Congestive heart failure*	12249 (46.83%)	12590 (48.14%)	9652 (42.67%)	9856 (43.57%)	2373 (23.86%)	2386 (23.99%)	14558 (25.29%)	14571 (25.31%)	2250 (22.19%)	2373 (23.4%)	2297 (22.64%)	2374 (23.4%)
	Peripheral vascular disease*	4464 (17.07%)	4350 (16.63%)	3404 (15.05%)	3378 (14.93%)	835 (8.4%)	851 (8.56%)	4928 (8.56%)	5061 (8.79%)	776 (7.65%)	837 (8.25%)	778 (7.67%)	838 (8.26%)
	Cerebrovascular disease*	5090 (19.46%)	4706 (17.99%)	3922 (17.34%)	3827 (16.92%)	1743 (17.52%)	1712 (17.21%)	6230 (10.82%)	6234 (10.83%)	1699 (16.75%)	1754 (17.3%)	1624 (16.01%)	1757 (17.32%)
	Dementia*	2325 (8.89%)	2139 (8.18%)	1687 (7.46%)	1687 (7.46%)	401 (4.03%)	399 (4.01%)	2314 (4.02%)	2367 (4.11%)	351 (3.46%)	401 (3.95%)	364 (3.59%)	401 (3.95%)
	Chronic pulmonary disease*	6930 (26.5%)	6490 (24.81%)	5977 (26.42%)	5483 (24.24%)	2092 (21.03%)	2154 (21.66%)	12454 (21.63%)	12601 (21.89%)	2043 (20.15%)	2109 (20.8%)	2035 (20.06%)	2109 (20.79%)
	Connective tissue disease*	748 (2.86%)	645 (2.47%)	592 (2.62%)	525 (2.32%)	154 (1.55%)	153 (1.54%)	923 (1.6%)	938 (1.63%)	138 (1.36%)	155 (1.53%)	136 (1.34%)	155 (1.53%)
	Ulcer disease*	347 (1.33%)	332 (1.27%)	227 (1%)	232 (1.03%)	75 (0.75%)	82 (0.82%)	331 (0.57%)	331 (0.57%)	68 (0.67%)	76 (0.75%)	56 (0.55%)	76 (0.75%)
	Mild liver disease*	555 (2.12%)	554 (2.12%)	443 (1.96%)	449 (1.99%)	107 (1.08%)	118 (1.19%)	628 (1.09%)	660 (1.15%)	105 (1.04%)	107 (1.06%)	108 (1.06%)	107 (1.05%)
	Diabetes*	7302 (27.92%)	7381 (28.22%)	6098 (26.96%)	6078 (26.87%)	2207 (22.19%)	2242 (22.54%)	12867 (22.35%)	12947 (22.49%)	2157 (21.27%)	2214 (21.83%)	2137 (21.06%)	2215 (21.83%)
	Diabetes with end-organ damage*	1199 (4.58%)	1381 (5.28%)	756 (3.34%)	858 (3.79%)	155 (1.56%)	171 (1.72%)	872 (1.51%)	889 (1.54%)	131 (1.29%)	155 (1.53%)	141 (1.39%)	155 (1.53%)
	Hemiplegia*	1747 (6.68%)	1618 (6.19%)	1286 (5.69%)	1275 (5.64%)	662 (6.66%)	652 (6.56%)	1843 (3.2%)	1836 (3.19%)	641 (6.32%)	671 (6.62%)	602 (5.93%)	673 (6.63%)
	Moderate or severe renal disease	5231 (20%)	6042 (23.1%)	2550 (11.27%)	3244 (14.34%)	422 (4.24%)	498 (5.01%)	2688 (4.67%)	2718 (4.72%)	413 (4.07%)	422 (4.16%)	405 (3.99%)	422 (4.16%)
	Any tumor (except for malignant neoplasm of skin)*	2675 (10.23%)	2490 (9.52%)	2304 (10.19%)	2081 (9.2%)	777 (7.81%)	834 (8.39%)	4017 (6.98%)	4086 (7.1%)	718 (7.08%)	778 (7.67%)	705 (6.95%)	779 (7.68%)
	Metastatic solid tumor*	566 (2.16%)	509 (1.95%)	481 (2.13%)	453 (2%)	172 (1.73%)	198 (1.99%)	828 (1.44%)	845 (1.47%)	153 (1.51%)	172 (1.7%)	163 (1.61%)	172 (1.7%)
	HIV/ AIDS*	46 (0.18%)	49 (0.19%)	51 (0.23%)	51 (0.23%)	9 (0.09%)	8 (0.08%)	55 (0.1%)	59 (0.1%)	12 (0.12%)	9 (0.09%)	9 (0.09%)	9 (0.09%)
	Moderate or severe liver disease*	125 (0.48%)	142 (0.54%)	87 (0.38%)	103 (0.46%)	24 (0.24%)	25 (0.25%)	102 (0.18%)	106 (0.18%)	16 (0.16%)	24 (0.24%)	30 (0.3%)	24 (0.24%)
	Hypertension	23403 (89.48%)	23781 (90.92%)	20029 (88.55%)	20312 (89.8%)	8331 (83.76%)	8415 (84.61%)	47269 (82.1%)	46904 (81.47%)	8434 (83.17%)	8432 (83.15%)	8417 (82.97%)	8436 (83.15%)
	Diabetes mellitus	7935 (30.34%)	8075 (30.87%)	6564 (29.02%)	6634 (29.33%)	2360 (23.73%)	2430 (24.43%)	13778 (23.93%)	13852 (24.06%)	2335 (23.03%)	2367 (23.34%)	2275 (22.42%)	2368 (23.34%)
	History of stroke, TIA, or VTE	3597 (13.75%)	3216 (12.3%)	2596 (11.48%)	2662 (11.77%)	1332 (13.39%)	1246 (12.53%)	4543 (7.89%)	4143 (7.2%)	1153 (11.37%)	1339 (13.2%)	1202 (11.85%)	1341 (13.22%)
	Stroke or TIA	3581 (13.69%)	3204 (12.25%)	2581 (11.41%)	2651 (11.72%)	1328 (13.35%)	1242 (12.49%)	4525 (7.86%)	4125 (7.16%)	1151 (11.35%)	1335 (13.16%)	1198 (11.81%)	1337 (13.18%)
	VTE	17 (0.06%)	14 (0.05%)	17 (0.08%)	12 (0.05%)	4 (0.04%)	5 (0.05%)	18 (0.03%)	21 (0.04%)	2 (0.02%)	4 (0.04%)	4 (0.04%)	4 (0.04%)
	Vascular disease + peripheral vascular stenting	9184 (35.11%)	8976 (34.32%)	7205 (31.85%)	7181 (31.75%)	1791 (18.01%)	1827 (18.37%)	10982 (19.07%)	11163 (19.39%)	1717 (16.93%)	1793 (17.68%)	1664 (16.4%)	1795 (17.69%)
	Peripheral vascular stenting	292 (1.12%)	256 (0.98%)	228 (1.01%)	216 (0.95%)	64 (0.64%)	66 (0.66%)	455 (0.79%)	460 (0.8%)	58 (0.57%)	64 (0.63%)	62 (0.61%)	64 (0.63%)
	Anemia and coagulation defects	4625 (17.68%)	4654 (17.79%)	3088 (13.65%)	3245 (14.35%)	773 (7.77%)	837 (8.42%)	3875 (6.73%)	4018 (6.98%)	748 (7.38%)	771 (7.6%)	691 (6.81%)	773 (7.62%)
	History of bleeding	5991 (22.91%)	6012 (22.99%)	4199 (18.56%)	4318 (19.09%)	1166 (11.72%)	1191 (11.97%)	5529 (9.6%)	5691 (9.88%)	1100 (10.85%)	1166 (11.5%)	1043 (10.28%)	1168 (11.51%)
	Thrombocytopenia	428 (1.64%)	434 (1.66%)	315 (1.39%)	321 (1.42%)	85 (0.85%)	83 (0.83%)	405 (0.7%)	417 (0.72%)	76 (0.75%)	85 (0.84%)	67 (0.66%)	85 (0.84%)
	Atherosclerotic disease	3114 (11.91%)	2975 (11.37%)	2304 (10.19%)	2232 (9.87%)	532 (5.35%)	527 (5.3%)	3352 (5.82%)	3353 (5.82%)	509 (5.02%)	534 (5.27%)	515 (5.08%)	534 (5.26%)
	Vascular disease	9184 (35.11%)	8973 (34.31%)	7202 (31.84%)	7178 (31.73%)	1791 (18.01%)	1824 (18.34%)	10980 (19.07%)	11159 (19.38%)	1716 (16.92%)	1793 (17.68%)	1664 (16.4%)	1795 (17.69%)
	Heart failure	10043 (38.4%)	10541 (40.3%)	7702 (34.05%)	8116 (35.88%)	1849 (18.59%)	1944 (19.55%)	11581 (20.12%)	11535 (20.04%)	1797 (17.72%)	1849 (18.23%)	1848 (18.22%)	1850 (18.24%)
	Dyspepsia or stomach discomfort	804 (3.07%)	720 (2.75%)	682 (3.02%)	594 (2.63%)	245 (2.46%)	242 (2.43%)	1375 (2.39%)	1390 (2.41%)	238 (2.35%)	247 (2.44%)	200 (1.97%)	247 (2.43%)
	Coronary artery disease	9584 (36.64%)	9605 (36.72%)	7801 (34.49%)	7832 (34.63%)	2030 (20.41%)	2062 (20.73%)	12838 (22.3%)	12933 (22.46%)	1940 (19.13%)	2030 (20.02%)	1930 (19.02%)	2031 (20.02%)
	Obesity (ICD-10 claims)	4403 (16.83%)	4262 (16.3%)	3756 (16.61%)	3564 (15.76%)	1208 (12.15%)	1241 (12.48%)	6687 (11.61%)	6862 (11.92%)	1129 (11.13%)	1212 (11.95%)	1122 (11.06%)	1213 (11.96%)
	Liver disease	608 (2.32%)	590 (2.26%)	475 (2.1%)	477 (2.11%)	114 (1.15%)	125 (1.26%)	674 (1.17%)	701 (1.22%)	114 (1.12%)	114 (1.12%)	121 (1.19%)	114 (1.12%)
	Chronic kidney disease	4970 (19%)	5621 (21.49%)	2371 (10.48%)	2889 (12.77%)	392 (3.94%)	431 (4.33%)	2447 (4.25%)	2496 (4.34%)	375 (3.7%)	392 (3.87%)	368 (3.63%)	392 (3.86%)
	Maximum stage 1	112 (0.43%)	109 (0.42%)	70 (0.31%)	81 (0.36%)	15 (0.15%)	16 (0.16%)	73 (0.13%)	77 (0.13%)	14 (0.14%)	15 (0.15%)	12 (0.12%)	15 (0.15%)
	Maximum stage 2	333 (1.27%)	329 (1.26%)	251 (1.11%)	283 (1.25%)	61 (0.61%)	55 (0.55%)	303 (0.53%)	311 (0.54%)	49 (0.48%)	61 (0.6%)	58 (0.57%)	61 (0.6%)
	Maximum stage 3	2493 (9.53%)	2640 (10.09%)	1256 (5.55%)	1543 (6.82%)	198 (1.99%)	214 (2.15%)	1284 (2.23%)	1307 (2.27%)	209 (2.06%)	198 (1.95%)	179 (1.76%)	198 (1.95%)
	Maximum stage 4	733 (2.8%)	958 (3.66%)	204 (0.9%)	279 (1.23%)	30 (0.3%)	49 (0.49%)	226 (0.39%)	204 (0.35%)	25 (0.25%)	30 (0.3%)	19 (0.19%)	30 (0.3%)
	Other/unknown	1299 (4.97%)	1585 (6.06%)	590 (2.61%)	703 (3.11%)	88 (0.88%)	97 (0.98%)	561 (0.97%)	597 (1.04%)	78 (0.77%)	88 (0.87%)	100 (0.99%)	88 (0.87%)
	Chronic obstructive pulmonary disease	151 (0.58%)	154 (0.59%)	126 (0.56%)	127 (0.56%)	36 (0.36%)	45 (0.45%)	176 (0.31%)	175 (0.3%)	29 (0.29%)	36 (0.35%)	25 (0.25%)	36 (0.35%)
	Hospitalization with alcohol discharge code	1081 (4.13%)	997 (3.81%)	966 (4.27%)	893 (3.95%)	291 (2.93%)	290 (2.92%)	1648 (2.86%)	1742 (3.03%)	292 (2.88%)	292 (2.88%)	270 (2.66%)	292 (2.88%)
**CHA**_**2**_ **DS**_**2**_ **-VASc score**	Mean (SD)	4.3 (1.6)	4.3 (1.5)	4.1 (1.5)	4.2 (1.5)	3.6 (1.6)	3.7 (1.5)	3.4 (1.6)	3.4 (1.6)	3.5 (1.6)	3.6 (1.6)	3.5 (1.6)	3.6 (1.6)
	0	228 (0.87%)	167 (0.64%)	249 (1.1%)	167 (0.74%)	218 (2.19%)	153 (1.54%)	1619 (2.81%)	2020 (3.51%)	259 (2.55%)	232 (2.29%)	257 (2.53%)	232 (2.29%)
	1	805 (3.08%)	702 (2.68%)	891 (3.94%)	696 (3.08%)	627 (6.3%)	563 (5.66%)	4672 (8.11%)	4968 (8.63%)	743 (7.33%)	683 (6.74%)	711 (7.01%)	683 (6.73%)
	2	2118 (8.1%)	1850 (7.07%)	2008 (8.88%)	1821 (8.05%)	1399 (14.07%)	1278 (12.85%)	9673 (16.8%)	9296 (16.15%)	1520 (14.99%)	1476 (14.55%)	1536 (15.14%)	1476 (14.55%)
	3	4221 (16.14%)	4403 (16.83%)	4141 (18.31%)	4224 (18.67%)	2326 (23.39%)	2603 (26.17%)	13958 (24.24%)	13749 (23.88%)	2422 (23.88%)	2360 (23.27%)	2409 (23.75%)	2360 (23.26%)
	≥4	18783 (71.81%)	19033 (72.77%)	15330 (67.77%)	15711 (69.46%)	5376 (54.05%)	5349 (53.78%)	27651 (48.03%)	27540 (47.83%)	5197 (51.25%)	5390 (53.15%)	5232 (51.57%)	5394 (53.17%)
**Concomitant treatment**	Antiplatelets*	21625 (82.68%)	22183 (84.81%)	18402 (81.36%)	19037 (84.16%)	7708 (77.5%)	7881 (79.24%)	43891 (76.24%)	43610 (75.75%)	7900 (77.9%)	7827 (77.18%)	7835 (77.23%)	7830 (77.18%)
	Aromatase inhibitors*	177 (0.68%)	163 (0.62%)	153 (0.68%)	147 (0.65%)	58 (0.58%)	52 (0.52%)	328 (0.57%)	330 (0.57%)	58 (0.57%)	58 (0.57%)	68 (0.67%)	58 (0.57%)
	NSAIDs*	3274 (12.52%)	2636 (10.08%)	3117 (13.78%)	2439 (10.78%)	1796 (18.06%)	1630 (16.39%)	10909 (18.95%)	11129 (19.33%)	1831 (18.06%)	1876 (18.5%)	1903 (18.76%)	1876 (18.49%)
	Corticosteroids*	6274 (23.99%)	5314 (20.32%)	5308 (23.47%)	4655 (20.58%)	2236 (22.48%)	2184 (21.96%)	13127 (22.8%)	13377 (23.23%)	2129 (20.99%)	2271 (22.39%)	2174 (21.43%)	2272 (22.4%)
	H2-receptor antagonists*	152 (0.58%)	145 (0.55%)	148 (0.65%)	127 (0.56%)	45 (0.45%)	33 (0.33%)	251 (0.44%)	264 (0.46%)	44 (0.43%)	45 (0.44%)	44 (0.43%)	45 (0.44%)
	Prostaglandins	858 (3.28%)	621 (2.37%)	885 (3.91%)	504 (2.23%)	349 (3.51%)	258 (2.59%)	2476 (4.3%)	2320 (4.03%)	341 (3.36%)	357 (3.52%)	405 (3.99%)	357 (3.52%)
	Proton pump inhibitors*	16332 (62.44%)	16204 (61.95%)	13625 (60.24%)	13613 (60.18%)	5142 (51.7%)	5252 (52.81%)	28301 (49.16%)	28443 (49.4%)	5081 (50.1%)	5182 (51.1%)	5131 (50.58%)	5185 (51.11%)
	Anticonvulsant strong inhibitor of hepatic enzymes*	221 (0.84%)	218 (0.83%)	202 (0.89%)	184 (0.81%)	60 (0.6%)	65 (0.65%)	314 (0.55%)	332 (0.58%)	51 (0.5%)	60 (0.59%)	51 (0.5%)	60 (0.59%)
	HIV protease inhibitors*	174 (0.67%)	135 (0.52%)	139 (0.61%)	114 (0.5%)	54 (0.54%)	59 (0.59%)	337 (0.59%)	349 (0.61%)	44 (0.43%)	54 (0.53%)	51 (0.5%)	54 (0.53%)
	Strong inhibitors of both CYP3A4 and P-gp*	585 (2.24%)	437 (1.67%)	464 (2.05%)	371 (1.64%)	218 (2.19%)	214 (2.15%)	1287 (2.24%)	1272 (2.21%)	189 (1.86%)	228 (2.25%)	205 (2.02%)	229 (2.26%)
	Statins*	5695 (21.77%)	5394 (20.62%)	4855 (21.46%)	4669 (20.64%)	2102 (21.13%)	2134 (21.46%)	11415 (19.83%)	11473 (19.93%)	2079 (20.5%)	2120 (20.91%)	2066 (20.36%)	2120 (20.9%)
	Selective estrogen receptor modulators*	48 (0.18%)	39 (0.15%)	41 (0.18%)	36 (0.16%)	19 (0.19%)	18 (0.18%)	93 (0.16%)	99 (0.17%)	17 (0.17%)	19 (0.19%)	15 (0.15%)	19 (0.19%)
	Selective serotonin reuptake inhibitors*	2590 (9.9%)	2290 (8.76%)	2053 (9.08%)	1920 (8.49%)	753 (7.57%)	772 (7.76%)	3802 (6.6%)	3870 (6.72%)	744 (7.34%)	762 (7.51%)	693 (6.83%)	762 (7.51%)
	Hormones	1107 (4.23%)	816 (3.12%)	1122 (4.96%)	681 (3.01%)	457 (4.59%)	363 (3.65%)	3204 (5.57%)	3074 (5.34%)	476 (4.69%)	471 (4.64%)	537 (5.29%)	471 (4.64%)
	Erythropoesis stimulating agents*	405 (1.55%)	454 (1.74%)	198 (0.88%)	235 (1.04%)	29 (0.29%)	32 (0.32%)	213 (0.37%)	215 (0.37%)	24 (0.24%)	29 (0.29%)	27 (0.27%)	29 (0.29%)
	Beta blockers*	16981 (64.92%)	17220 (65.84%)	14570 (64.41%)	14792 (65.4%)	5909 (59.41%)	5887 (59.19%)	34919 (60.65%)	34595 (60.09%)	5922 (58.4%)	5970 (58.87%)	5968 (58.83%)	5973 (58.88%)
	Antiarrhythmic agents*	13237 (50.61%)	13554 (51.82%)	11962 (52.88%)	12120 (53.58%)	6276 (63.1%)	6271 (63.05%)	36741 (63.82%)	36823 (63.96%)	6587 (64.95%)	6457 (63.67%)	6598 (65.04%)	6460 (63.68%)

*Variables that were adjusted for in the PS model. Scores (Charlson Comorbidity Index, HAS-BLED and CHA_2_DS_2_-VASc) were not included in the PS modelling as their components are singularly included, but were used as indicator for evaluating the fitness of the matching.

AIDS, acquired immunodeficiency syndrome; CKD, chronic kidney disease; CYP3A4, cytochrome P450 3A4; DOAC, direct oral anticoagulant; GIB, gastrointestinal bleed; HIV, human immunodeficiency virus; LTR, long-term recurrence; NSAID, nonsteroidal anti-inflammatory drug; P-gp, P-glycoprotein; PS, propensity score; SD, standard deviation; TIA, transient ischemic attack; VKA, vitamin K antagonist; VTE, venous thromboembolism.

**Table 4:** Baseline characteristics after PS matching for patients with CKD stage 3 or 4.

		Pairwise comparison		Pairwise comparison		Pairwise comparison	
Characteristic		Apixaban (n = 5,908)	VKAs (n = 5,908)	Rivaroxaban (n 2,638)	VKAs (n = 2,638)	Apixaban (n = 2,650)	Rivaroxaban (n = 2,650)
**Index dosage**	Standard dose	-	-	-	-	772 (29.13%)	742 (28.00%)
	Reduced dose	-	-	-	-	1878 (70.87%)	1908 (72.00%)
**Atrial fibrillation identification setting**	Inpatient claim with I48 code	5413 (91.62%)	5432 (91.94%)	2330 (88.32%)	2326 (88.17%)	2330 (87.92%)	2338 (88.23%)
	LTR registration with I48 code*	45 (0.76%)	42 (0.71%)	34 (1.29%)	31 (1.18%)	39 (1.47%)	36 (1.36%)
	Use of anti-arrhythmic drugs*	450 (7.62%)	434 (7.35%)	274 (10.39%)	281 (10.65%)	281 (10.6%)	276 (10.42%)
**Follow up time (months), censored at switch, discontinuation, interruption, death, pregnancy, dialysis, CKD stage V, or end of follow up, mean (SD)**		10.4 [10.3]	10.4 [10.7]	10.4 [10.7]	10.6 [10.8]	10.5 [10.6]	10.5 [10.7]
**Age at index date (years), mean (SD)**		84.0 (8.8)	83.8 (8.8)	82.2 (9.4)	82.4 (9.4)	82.2 (9.4)	82.2 (9.4)
**Age groups at index date**	18–54 years	28 (0.47%)	27 (0.46%)	20 (0.76%)	19 (0.72%)	20 (0.75%)	19 (0.72%)
	55–64 years*	161 (2.73%)	171 (2.89%)	102 (3.87%)	99 (3.75%)	106 (4%)	102 (3.85%)
	65–74 years*	679 (11.49%)	690 (11.68%)	415 (15.73%)	408 (15.47%)	420 (15.85%)	419 (15.81%)
	75–79 years*	592 (10.02%)	630 (10.66%)	358 (13.57%)	336 (12.74%)	366 (13.81%)	362 (13.66%)
	≥80 years*	4448 (75.29%)	4390 (74.31%)	1743 (66.07%)	1776 (67.32%)	1738 (65.58%)	1748 (65.96%)
**Sex**	Male	2707 (45.82%)	2865 (48.49%)	1320 (50.04%)	1321 (50.08%)	1339 (50.53%)	1326 (50.04%)
	Female*	3201 (54.18%)	3043 (51.51%)	1318 (49.96%)	1317 (49.92%)	1311 (49.47%)	1324 (49.96%)
**GIB risk factors**	Age ≥75 years	5040 (85.31%)	5020 (84.97%)	2101 (79.64%)	2112 (80.06%)	2104 (79.4%)	2110 (79.62%)
	HAS-BLED score, mean	4.1 (0.9)	4.1 (0.9)	4.0 (0.9)	4.0 (0.9)	4.0 (0.9)	4.0 (0.9)
	0	0	0	0	0	0	0
	1	8 (0.14%)	1 (0.02%)	4 (0.15%)	1 (0.04%)	6 (0.23%)	5 (0.19%)
	2	146 (2.47%)	140 (2.37%)	86 (3.26%)	89 (3.37%)	93 (3.51%)	87 (3.28%)
	≥3*	5754 (97.39%)	5767 (97.61%)	2548 (96.59%)	2548 (96.59%)	2551 (96.26%)	2558 (96.53%)
	Prior medications (antiplatelets, NSAIDs, or corticosteroids)	3345 (56.62%)	3373 (57.09%)	1503 (56.97%)	1527 (57.88%)	1487 (56.11%)	1505 (56.79%)
	Renal impairment (CKD stage 3–4)	5908 (100%)	5908 (100%)	2638 (100%)	2638 (100%)	2650 (100%)	2650 (100%)
	Prior GI condition*	595 (10.07%)	632 (10.7%)	264 (10.01%)	271 (10.27%)	282 (10.64%)	270 (10.19%)
**Number of GIB risk factors**	1	52 (0.88%)	42 (0.71%)	36 (1.36%)	26 (0.99%)	36 (1.36%)	36 (1.36%)
	2	315 (5.33%)	287 (4.86%)	204 (7.73%)	163 (6.18%)	206 (7.77%)	207 (7.81%)
	3	2489 (42.13%)	2476 (41.91%)	1110 (42.08%)	1152 (43.67%)	1132 (42.72%)	1114 (42.04%)
	4	2767 (46.83%)	2859 (48.39%)	1160 (43.97%)	1197 (45.38%)	1150 (43.4%)	1164 (43.92%)
	5	285 (4.82%)	244 (4.13%)	128 (4.85%)	100 (3.79%)	126 (4.75%)	129 (4.87%)
**Charlson Comorbidity Index score**	Mean (SD)	4.8 [2.2]	4.9 [2.2]	4.7 [2.2]	4.7 [2.1]	4.7 [2.2]	4.7 [2.2]
	0	0	0	0	0	0	0
	1 or 2	431 (7.3%)	425 (7.19%)	251 (9.51%)	245 (9.29%)	218 (8.23%)	256 (9.66%)
	3 or 4	2722 (46.07%)	2654 (44.92%)	1259 (47.73%)	1260 (47.76%)	1274 (48.08%)	1263 (47.66%)
	≥5	2755 (46.63%)	2829 (47.88%)	1128 (42.76%)	1133 (42.95%)	1158 (43.7%)	1131 (42.68%)
**Comorbidities**	Myocardial infarction*	797 (13.49%)	854 (14.45%)	312 (11.83%)	325 (12.32%)	319 (12.04%)	312 (11.77%)
	Congestive heart failure*	3729 (63.12%)	3890 (65.84%)	1538 (58.3%)	1536 (58.23%)	1573 (59.36%)	1541 (58.15%)
	Peripheral vascular disease*	1145 (19.38%)	1217 (20.6%)	453 (17.17%)	448 (16.98%)	452 (17.06%)	451 (17.02%)
	Cerebrovascular disease*	1360 (23.02%)	1293 (21.89%)	503 (19.07%)	517 (19.6%)	465 (17.55%)	506 (19.09%)
	Dementia*	1091 (18.47%)	985 (16.67%)	459 (17.4%)	484 (18.35%)	468 (17.66%)	462 (17.43%)
	Chronic pulmonary disease*	1587 (26.86%)	1605 (27.17%)	744 (28.2%)	726 (27.52%)	751 (28.34%)	745 (28.11%)
	Connective tissue disease*	174 (2.95%)	151 (2.56%)	59 (2.24%)	60 (2.27%)	59 (2.23%)	59 (2.23%)
	Ulcer disease*	103 (1.74%)	108 (1.83%)	44 (1.67%)	43 (1.63%)	43 (1.62%)	44 (1.66%)
	Mild liver disease*	144 (2.44%)	155 (2.62%)	80 (3.03%)	87 (3.3%)	81 (3.06%)	79 (2.98%)
	Diabetes*	1943 (32.89%)	2026 (34.29%)	874 (33.13%)	879 (33.32%)	876 (33.06%)	874 (32.98%)
	Diabetes with end-organ damage*	757 (12.81%)	849 (14.37%)	315 (11.94%)	301 (11.41%)	324 (12.23%)	315 (11.89%)
	Hemiplegia*	524 (8.87%)	501 (8.48%)	176 (6.67%)	184 (6.97%)	174 (6.57%)	176 (6.64%)
	Moderate or severe renal disease	5908 (100%)	5908 (100%)	2638 (100%)	2638 (100%)	2650 (100%)	2650 (100%)
	Any tumor (except for malignant neoplasm of skin)*	699 (11.83%)	740 (12.53%)	302 (11.45%)	314 (11.9%)	320 (12.08%)	304 (11.47%)
	Metastatic solid tumor*	99 (1.68%)	110 (1.86%)	59 (2.24%)	57 (2.16%)	65 (2.45%)	61 (2.3%)
	HIV/ AIDS*	6 (0.1%)	7 (0.12%)	6 (0.23%)	4 (0.15%)	6 (0.23%)	6 (0.23%)
	Moderate or severe liver disease*	20 (0.34%)	27 (0.46%)	8 (0.3%)	7 (0.27%)	10 (0.38%)	8 (0.3%)
	Hypertension	5656 (95.73%)	5693 (96.36%)	2483 (94.12%)	2498 (94.69%)	2472 (93.28%)	2487 (93.85%)
	Diabetes mellitus	2139 (36.21%)	2236 (37.85%)	956 (36.24%)	967 (36.66%)	983 (37.09%)	958 (36.15%)
	History of stroke, TIA, or VTE	1019 (17.25%)	906 (15.34%)	335 (12.7%)	353 (13.38%)	332 (12.53%)	337 (12.72%)
	Stroke or TIA	1017 (17.21%)	902 (15.27%)	333 (12.62%)	351 (13.31%)	330 (12.45%)	333 (12.57%)
	VTE	2 (0.03%)	4 (0.07%)	2 (0.08%)	2 (0.08%)	2 (0.08%)	4 (0.15%)
	Vascular disease + peripheral vascular stenting	2224 (37.64%)	2294 (38.83%)	916 (34.72%)	921 (34.91%)	919 (34.68%)	916 (34.57%)
	Peripheral vascular stenting	51 (0.86%)	60 (1.02%)	23 (0.87%)	20 (0.76%)	22 (0.83%)	23 (0.87%)
	Anemia and coagulation defects	2058 (34.83%)	2088 (35.34%)	798 (30.25%)	831 (31.5%)	858 (32.38%)	805 (30.38%)
	History of bleeding	2381 (40.3%)	2434 (41.2%)	952 (36.09%)	991 (37.57%)	981 (37.02%)	959 (36.19%)
	Thrombocytopenia	141 (2.39%)	152 (2.57%)	64 (2.43%)	64 (2.43%)	60 (2.26%)	64 (2.42%)
	Atherosclerotic disease	839 (14.2%)	928 (15.71%)	341 (12.93%)	335 (12.7%)	333 (12.57%)	339 (12.79%)
	Vascular disease	2224 (37.64%)	2293 (38.81%)	916 (34.72%)	921 (34.91%)	919 (34.68%)	916 (34.57%)
	Heart failure	3376 (57.14%)	3550 (60.09%)	1360 (51.55%)	1393 (52.81%)	1419 (53.55%)	1362 (51.4%)
	Dyspepsia or stomach discomfort	243 (4.11%)	241 (4.08%)	120 (4.55%)	126 (4.78%)	124 (4.68%)	121 (4.57%)
	Coronary artery disease	2047 (34.65%)	2147 (36.34%)	871 (33.02%)	888 (33.66%)	887 (33.47%)	869 (32.79%)
	Obesity (ICD-10 claims)	1250 (21.16%)	1278 (21.63%)	589 (22.33%)	595 (22.55%)	599 (22.6%)	592 (22.34%)
	Liver disease	153 (2.59%)	162 (2.74%)	83 (3.15%)	89 (3.37%)	84 (3.17%)	82 (3.09%)
	Chronic kidney disease	5908 (100%)	5908 (100%)	2638 (100%)	2638 (100%)	2650 (100%)	2650 (100%)
	Maximum stage 1	-	-	-	-	-	-
	Maximum stage 2	-	-	-	-	-	-
	Maximum stage 3	4608 (78%)	4564 (77.25%)	2291 (86.85%)	2313 (87.68%)	2209 (83.36%)	2303 (86.91%)
	Maximum stage 4	1300 (22%)	1344 (22.75%)	347 (13.15%)	325 (12.32%)	441 (16.64%)	347 (13.09%)
	Other/unknown	-	-	-	-	-	-
	Chronic obstructive pulmonary disease	36 (0.61%)	38 (0.64%)	13 (0.49%)	14 (0.53%)	15 (0.57%)	13 (0.49%)
	Hospitalization with alcohol discharge code	215 (3.64%)	233 (3.94%)	149 (5.65%)	146 (5.53%)	152 (5.74%)	151 (5.7%)
**CHA**_**2**_ **DS**_**2**_ **-VASc score**	Mean (SD)	5.0 (1.4)	5.0 (1.3)	4.7 (1.4)	4.8 (1.3)	4.7 (1.4)	4.7 (1.4)
	0	3 (0.05%)	2 (0.03%)	2 (0.08%)	3 (0.11%)	3 (0.11%)	4 (0.15%)
	1	38 (0.64%)	24 (0.41%)	23 (0.87%)	18 (0.68%)	25 (0.94%)	22 (0.83%)
	2	121 (2.05%)	122 (2.06%)	101 (3.83%)	85 (3.22%)	98 (3.7%)	102 (3.85%)
	3	489 (8.28%)	507 (8.58%)	324 (12.28%)	305 (11.56%)	301 (11.36%)	328 (12.38%)
	≥4	5257 (88.98%)	5253 (88.91%)	2188 (82.94%)	2227 (84.42%)	2223 (83.89%)	2194 (82.79%)
**Concomitant treatment**	Antiplatelets*	2882 (48.78%)	2925 (49.51%)	1275 (48.33%)	1289 (48.86%)	1261 (47.58%)	1277 (48.19%)
	Aromatase inhibitors*	59 (1%)	48 (0.81%)	15 (0.57%)	16 (0.61%)	17 (0.64%)	14 (0.53%)
	NSAIDs*	314 (5.31%)	287 (4.86%)	171 (6.48%)	153 (5.8%)	186 (7.02%)	170 (6.42%)
	Corticosteroids*	822 (13.91%)	775 (13.12%)	391 (14.82%)	387 (14.67%)	409 (15.43%)	393 (14.83%)
	H2-receptor antagonists*	26 (0.44%)	29 (0.49%)	12 (0.45%)	12 (0.45%)	13 (0.49%)	12 (0.45%)
	Prostaglandins*	170 (2.88%)	156 (2.64%)	94 (3.56%)	64 (2.43%)	105 (3.96%)	93 (3.51%)
	Proton pump inhibitors*	3326 (56.3%)	3397 (57.5%)	1447 (54.85%)	1453 (55.08%)	1455 (54.91%)	1447 (54.6%)
	Anticonvulsant strong inhibitor of hepatic enzymes*	40 (0.68%)	42 (0.71%)	20 (0.76%)	20 (0.76%)	22 (0.83%)	21 (0.79%)
	HIV protease inhibitors*	28 (0.47%)	29 (0.49%)	15 (0.57%)	14 (0.53%)	23 (0.87%)	15 (0.57%)
	Strong inhibitors of both CYP3A4 and P-gp*	136 (2.3%)	123 (2.08%)	50 (1.9%)	46 (1.74%)	62 (2.34%)	51 (1.92%)
	Statins*	924 (15.64%)	944 (15.98%)	450 (17.06%)	427 (16.19%)	432 (16.3%)	449 (16.94%)
	Selective estrogen receptor modulators*	4 (0.07%)	4 (0.07%)	4 (0.15%)	4 (0.15%)	3 (0.11%)	5 (0.19%)
	Selective serotonin reuptake inhibitors*	624 (10.56%)	591 (10%)	281 (10.65%)	280 (10.61%)	286 (10.79%)	282 (10.64%)
	Hormones	216 (3.66%)	185 (3.13%)	122 (4.62%)	80 (3.03%)	127 (4.79%)	121 (4.57%)
	Erythropoesis stimulating agents*	181 (3.06%)	190 (3.22%)	48 (1.82%)	57 (2.16%)	48 (1.81%)	48 (1.81%)
	Beta blockers*	3855 (65.25%)	3902 (66.05%)	1735 (65.77%)	1708 (64.75%)	1718 (64.83%)	1737 (65.55%)
	Antiarrhythmic agents*	2311 (39.12%)	2318 (39.23%)	1143 (43.33%)	1121 (42.49%)	1164 (43.92%)	1150 (43.4%)

*Variables that were adjusted for in the PS model. Scores (Charlson Comorbidity Index, HAS-BLED and CHA_2_DS_2_-VASc) were not included in the PS modelling as their components are singularly included, but were used as indicator for evaluating the fitness of the matching.

AIDS, acquired immunodeficiency syndrome; CKD, chronic kidney disease; CYP3A4, cytochrome P450 3A4; DOAC, direct oral anticoagulant; GIB, gastrointestinal bleed; HIV, human immunodeficiency virus; LTR, long-term recurrence; NSAID, nonsteroidal anti-inflammatory drug; P-gp, P-glycoprotein; PS, propensity score; SD, standard deviation; TIA, transient ischemic attack; VKA, vitamin K antagonist; VTE, venous thromboembolism.

### Patients age ≥75 years

#### VKA versus DOAC

After PS matching among patients age ≥75 years, apixaban (HR, 0.53, 95% CI, 0.50–0.58), rivaroxaban (HR, 0.84; 95% CI, 0.78–0.91), and dabigatran (HR, 0.62; 95% CI, 0.55–0.71) use were associated with a lower risk of major bleeding leading to hospitalization than VKAs (*p*<0.0001 for all; Fig 2). Patients receiving apixaban had a lower risk of GIB versus those receiving VKAs (HR, 0.53; 95% CI, 0.47–0.60; *p*<0.0001). Rivaroxaban (HR, 0.99; 95% CI, 0.88–1.11; *p* = 0.85) and dabigatran (HR, 1.09; 95% CI, 0.89–1.32; *p* = 0.40) were associated with similar risk of GIB versus VKAs. Compared to VKAs, DOACs were similarly associated with a lower risk of ICH (apixaban HR, 0.54, 95% CI, 0.47–0.63; rivaroxaban HR, 0.62; 95% CI, 0.53–0.72; dabigatran HR, 0.31; 95% CI, 0.23–0.42) and other bleeds (apixaban HR, 0.52, 95% CI, 0.47–0.59; rivaroxaban HR, 0.87; 95% CI, 0.78–0.98; dabigatran HR, 0.49; 95% CI, 0.40–0.61; *p*<0.02 for all).

Apixaban (HR, 0.73, 95% CI, 0.67–0.80), rivaroxaban (HR, 0.74; 95% CI, 0.68–0.82), and dabigatran (HR, 0.65; 95% CI, 0.55–0.77) use were associated with lower risk of stroke/SE versus VKAs (*p*<0.0001 for all).

#### DOAC versus DOAC

Among patients age ≥75 years, apixaban use was associated with a lower risk of major bleeding versus dabigatran (HR, 0.67; 95% CI, 0.58–0.78; *p*<0.0001) and rivaroxaban (HR, 0.61; 95% CI, 0.57–0.65; *p*<0.0001; Fig 3). Risk of major bleeding was lower with dabigatran use versus rivaroxaban (HR, 0.78; 95% CI, 0.69–0.90; *p*<0.001). Apixaban use was also associated with a lower risk of GIB versus dabigatran (HR, 0.42; 95% CI, 0.33–0.53; *p*<0.0001) and rivaroxaban (HR, 0.52; 95% CI, 0.46–0.58; *p*<0.0001). Risk of GIB was similar for dabigatran and rivaroxaban (HR, 1.21, 95% CI, 0.99–1.46; *p* = 0.06). Risk of ICH was similar when comparing apixaban with rivaroxaban (HR, 0.90; 95% CI, 0.79–1.03; *p* = 0.12) and apixaban versus dabigatran (HR, 1.34; 95% CI, 0.94–1.91; *p* = 0.10). Dabigatran use was associated with a lower risk of ICH versus rivaroxaban (HR, 0.56, 95% CI, 0.40–0.78; *p*<0.001). Apixaban use was associated with a similar risk of other bleeding versus dabigatran (HR, 0.80; 95% CI, 0.62–1.04; *p =* 0.09) whereas risk was lower versus rivaroxaban (HR, 0.56; 95% CI, 0.51–0.62; *p*<0.0001). Dabigatran use was associated with a lower risk of other bleeding versus rivaroxaban (HR, 0.55; 95% CI, 0.44–0.69; *p*<0.0001).

Compared with rivaroxaban, apixaban use was associated with a lower risk of stroke/SE (HR, 0.88; 95% CI, 0.81–0.95; *p* = 0.002). Risk of stroke/SE was similar when comparing apixaban with dabigatran (HR, 0.90; 95% CI, 0.74–1.09; *p* = 0.27) and dabigatran with rivaroxaban (HR, 0.89, 95% CI, 0.74–1.06; *p* = 0.19).

### Patients with HAS-BLED score ≥3

#### VKA versus DOAC

Among patients with HAS-BLED score ≥3, apixaban (HR, 0.52, 95% CI, 0.49–0.56), rivaroxaban (HR, 0.77; 95% CI, 0.72–0.83), and dabigatran (HR, 0.59; 95% CI, 0.51–0.68) use were associated with a lower risk of major bleeding leading to hospitalization than VKAs (*p*<0.0001 for all; Fig 4). Patients receiving apixaban had a lower risk of GIB versus those receiving VKAs (HR, 0.54; 95% CI, 0.48–0.61; *p*<0.0001). Rivaroxaban (HR, 0.92; 95% CI, 0.82–1.04; *p* = 0.18) and dabigatran (HR, 0.95; 95% CI, 0.77–1.17; *p* = 0.64) were similar with regard to risk of GIB versus VKAs.

Compared to VKAs, DOACs were similarly associated with a lower risk of ICH (apixaban HR, 0.52, 95% CI, 0.45–0.60; rivaroxaban HR, 0.49; 95% CI, 0.42–0.59; dabigatran HR, 0.26; 95% CI, 0.18–0.37) and other bleeds (apixaban HR, 0.50, 95% CI, 0.45–0.56; rivaroxaban HR, 0.81; 95% CI, 0.72–0.90; dabigatran HR, 0.51; 95% CI, 0.41–0.63; *p*<0.001 for all). Apixaban (HR, 0.74, 95% CI, 0.68–0.81), rivaroxaban (HR, 0.76; 95% CI, 0.69–0.84), and dabigatran (HR, 0.72; 95% CI, 0.60–0.86) use were associated with lower risk of stroke/SE versus VKAs (*p*<0.001 for all).

#### DOAC versus DOAC

Among patients with HAS-BLED score ≥3, risk of major bleeding was lower with apixaban use versus rivaroxaban (HR, 0.64; 95% CI, 0.60–0.68; *p*<0.0001; Fig 5) and versus dabigatran (HR, 0.74; 95% CI, 0.63–0.87; *p* = 0.0003); risk was lower with dabigatran use versus rivaroxaban (HR, 0.78, 95% CI, 0.67–0.90; *p* = 0.0006). Apixaban use was associated with a lower risk of GIB versus rivaroxaban (HR, 0.54; 95% CI, 0.48–0.60; *p*<0.0001) and versus dabigatran (HR, 0.49; 95% CI, 0.38–0.63; *p*<0.0001), whereas risk was similar with dabigatran versus rivaroxaban (HR, 1.03, 95% CI, 0.84–1.27; *p* = 0.78).

Risk of ICH (HR, 0.89; 95% CI, 0.76–1.04; *p* = 0.13) was similar between apixaban and rivaroxaban; risk was lower with dabigatran versus apixaban (HR, 1.89; 95% CI, 1.28–2.79; *p* = 0.0014) and versus rivaroxaban (HR, 0.54, 95% CI, 0.36–0.80; *p* = 0.0023). Risk of other bleeding was lower with apixaban use versus dabigatran (HR, 0.73; 95% CI, 0.56–0.95; *p* = 0.0207) and versus rivaroxaban (HR, 0.64, 95% CI, 0.58–0.71; *p*<0.0001) as well as with dabigatran use versus rivaroxaban (HR, 0.62, 95% CI, 0.49–0.77; *p*<0.0001). Risk of stroke/SE was lower with apixaban use versus rivaroxaban (HR, 0.90, 95% CI, 0.82–0.98; *p* = 0.0135) and was similar with dabigatran versus apixaban (HR, 1.05; 95% CI, 0.87–1.26; *p* = 0.6063) as well as dabigatran versus rivaroxaban (HR, 0.91, 95% CI, 0.76–1.10; *p* = 0.32).

### Patients receiving concomitant antiplatelets, NSAIDs, or corticosteroids associated with GIB risk

#### VKA versus DOAC

Apixaban (HR, 0.45, 95% CI, 0.42–0.49), rivaroxaban (HR, 0.75; 95% CI, 0.69–0.82), and dabigatran (HR, 0.54; 95% CI, 0.47–0.63) use were associated with a lower risk of major bleeding leading to hospitalization than VKAs (*p*<0.0001 for all; Fig 6) among patients receiving concomitant antiplatelets, NSAIDs, or corticosteroids. Apixaban (HR, 0.48; 95% CI, 0.42–0.55; *p*<0.0001) and rivaroxaban (HR, 0.87; 95% CI, 0.77–0.99; *p* = 0.04) also had a lower risk of GIB versus VKAs. Dabigatran (HR, 0.90; 95% CI, 0.72–1.12; *p* = 0.36) was associated with similar risk of GIB versus VKAs.

Compared to VKAs, DOACs were associated with a lower risk of ICH (apixaban HR, 0.47, 95% CI, 0.39–0.57; rivaroxaban HR, 0.51; 95% CI, 0.42–0.62; dabigatran HR, 0.26; 95% CI, 0.18–0.38) and other bleeds (apixaban HR, 0.42, 95% CI, 0.37–0.48; rivaroxaban HR, 0.78; 95% CI, 0.69–0.88; dabigatran HR, 0.44; 95% CI, 0.35–0.56; *p*<0.0001 for all). Apixaban (HR, 0.74, 95% CI, 0.67–0.83), rivaroxaban (HR, 0.82; 95% CI, 0.74–0.92), and dabigatran (HR, 0.64; 95% CI, 0.53–0.77) use were associated with lower risk of stroke/SE versus VKAs (*p*<0.001 for all).

#### DOAC versus DOAC

Among patients receiving concomitant antiplatelets, NSAIDs, or corticosteroids, apixaban use was associated with a lower risk of major bleeding versus dabigatran (HR, 0.80; 95% CI, 0.68–0.95; *p* = 0.009) and rivaroxaban (HR, 0.61; 95% CI, 0.57–0.65; *p*<0.0001; Fig 7). Compared with rivaroxaban, dabigatran use was associated with a lower risk of major bleeding (HR, 0.79; 95% CI, 0.68–0.92; *p* = 0.003). Apixaban use was also associated with a lower risk of GIB versus dabigatran (HR, 0.56; 95% CI, 0.44–0.72; *p*<0.0001) and rivaroxaban (HR, 0.53; 95% CI, 0.48–0.59; *p*<0.0001). Risk of GIB was similar with dabigatran and rivaroxaban (HR, 1.10, 95% CI, 0.88–1.38; *p* = 0.39).

Risk of ICH was similar when comparing apixaban with rivaroxaban (HR, 0.86; 95% CI, 0.74–1.01). Apixaban use was associated with a higher risk of ICH versus dabigatran (HR, 1.44; 95% CI, 0.94–2.21), and dabigatran use was associated with a lower risk versus rivaroxaban (HR, 0.61; 95% CI, 0.40–0.93; *p* = 0.02). Risk of other bleeding was similar with apixaban versus dabigatran (HR, 0.94; 95% CI, 0.72–1.22; *p* = 0.64), whereas risk was lower versus rivaroxaban (HR, 0.59; 95% CI, 0.53–0.65; *p*<0.0001). This was also lower with dabigatran versus rivaroxaban (HR, 0.60, 95% CI, 0.48–0.76; *p*<0.0001). Compared with rivaroxaban, apixaban use was associated with a lower risk of stroke/SE (HR, 0.89; 95% CI, 0.82–0.97; *p* = 0.01). Risk of stroke/SE was similar when comparing apixaban with dabigatran (HR, 0.96; 95% CI, 0.79–1.18) and dabigatran with rivaroxaban (HR, 0.92; 95% CI, 0.75–1.12).

### Patients with CKD stage 3 or 4

#### VKA versus DOAC

In patients with CKD stage 3 or 4, apixaban (HR, 0.51, 95% CI, 0.43–0.60; *p*<0.0001) and rivaroxaban (HR, 0.62; 95% CI, 0.49–0.78; *p*<0.0001) use were associated with a lower risk of major bleeding leading to hospitalization than VKAs (Fig 8). Apixaban had a lower risk of GIB versus VKAs (HR, 0.54; 95% CI, 0.41–0.71; *p*<0.0001), whereas rivaroxaban had similar risk (HR, 0.81; 95% CI, 0.57–1.16; *p* = 0.26). Risk of ICH was reduced with apixaban (HR, 0.53, 95% CI, 0.38–0.75; *p*<0.001) and rivaroxaban (HR, 0.31, 95% CI, 0.17–0.55; *p*<0.0001) versus VKAs; this was similar for risk of other bleeds with apixaban (HR, 0.47, 95% CI, 0.36–0.61; *p*<0.0001) and rivaroxaban (HR, 0.64, 95% CI, 0.45–0.90; *p* = 0.0096) versus VKAs. Apixaban (HR, 0.71, 95% CI, 0.57–0.87; *p* = 0.0014) use was also associated with lower risk of stroke/SE versus VKAs. Rivaroxaban (HR, 0.87, 95% CI, 0.63–1.19; *p* = 0.38) use was similar with regard to risk of stroke/SE versus VKAs.

#### DOAC versus DOAC

Among patients with CKD stage 3 or 4, risk of major bleeding was lower with apixaban use versus rivaroxaban (HR, 0.76; 95% CI, 0.58–1.00; *p* = 0.0495; Fig 9). Apixaban use was associated with a lower risk of GIB versus rivaroxaban (HR, 0.64; 95% CI, 0.41–0.98; *p* = 0.0394) and a similar risk of ICH versus rivaroxaban (HR, 1.52; 95% CI, 0.80–2.92; *p* = 0.20). Risks of other bleeding (HR, 0.71, 95% CI, 0.47–1.07; *p* = 0.10) and stroke/SE (HR, 0.81; 95% CI, 0.57–1.15; *p* = 0.23) were similar when comparing apixaban with rivaroxaban.

### Sensitivity analyses

Results of AFT analyses were similar to the main analyses using Cox proportional hazards models. Estimated relative acceleration factors favored DOACs versus VKAs for risk of major bleedings (*p*<0.01 for all) as well as most outcomes of interest; relative acceleration factors for DOAC–DOAC comparisons were generally consistent with results from the main analyses (S6–S9 Tables).

## Discussion

### Overview of results

Stroke prevention in patients with NVAF can be challenging, requiring a balance between prevention of thromboembolism and serious bleeding, and made more complicated in those subgroups of patients with additional risk factors for bleeds. In this analysis using a large, real-world French dataset, we report that adults with NVAF at high risk for GIB with key risk factors for bleeding who received apixaban, dabigatran, and rivaroxaban had lower risks of major bleedings, GIB, ICH, and stroke/SE versus those who received VKAs. Second, apixaban was associated with lower risks of major bleeding and GIB versus rivaroxaban and dabigatran in these patient subgroups.

These findings are consistent with previous reports of observational studies such as ARISTOPHANES and NAXOS, which evaluated comparative effectiveness and safety in patients with NVAF [6, 14]. Our results highlight the importance of evaluating DOACs individually in patients with NVAF who have additional risk factors for major bleeding, as differences in efficacy/effectiveness and safety between agents have been reported in both clinical and observational studies [6, 14–17].

### Comparison with results from other studies in key subgroups

The ARISTOPHANES study evaluated elderly patients who were stratified as either age 75–79 or ≥80 years; apixaban was the only DOAC to be associated with lower risk of major bleeding versus warfarin in both age groups, whereas dabigatran was associated with similar risk in patients aged ≥80 years and rivaroxaban was associated with a similar risk in patients aged 75–79 and a higher risk in patients aged ≥80 years [14]. Similar to the results of the current analysis, all three DOACs were associated with lower risk of stroke/SE versus warfarin in both age groups of elderly patients (aged 75–79 and ≥80 years) [14]. Among patients age ≥75 years from the ROCKET AF trial, rivaroxaban showed non-significantly different risks of stroke/SE versus warfarin and major/clinically relevant bleeding [17]. In a recent retrospective cohort study among elderly patients age ≥80 years with NVAF, DOACs (apixaban, rivaroxaban, dabigatran, and edoxaban) as a group were associated with lower risks of both major bleeding and ischemic stroke versus warfarin [18]. Similar results were reported by Chao et al in an analysis of a Taiwanese national health insurance database; patients age ≥65 years who received apixaban, rivaroxaban, or dabigatran had lower risks of both major bleeding and ischemic stroke versus warfarin [19]. Results were also generally consistent when stratifying further by age [19]. Indeed, DOACs have associated with a lower risk of ischemic stroke, mortality, and a composite endpoint of ischemic stroke, ICH, major bleed, and mortality, when compared with non-OAC use in high-risk elderly AF patients at increased bleeding risk [20].

The HAS-BLED score is a widely used measure developed to estimate risk of major bleeding in patients receiving anticoagulants in order to assess risks and benefits in NVAF care [21]. It incorporates the following risk factors: hypertension, abnormal renal/liver function, stroke, bleeding history or predisposition, labile international normalized ratio, elderly [age ≥65 years], and concomitant drugs/alcohol; patients with a score of ≥3 are considered at high risk for bleeding. HAS-BLED is the most advocated scoring system for patients with NVAF receiving anticoagulant therapy [22], and has been validated even in DOAC users [23, 24]. In the subgroup of patients from ARISTOPHANES with HAS-BLED score ≥3, apixaban and dabigatran both were associated with lower risk of major bleeding versus warfarin, whereas risk was similar with rivaroxaban; all three DOACs were associated with lower risk of stroke/SE versus warfarin [25]. A recent population-based study in Korean patients with NVAF found that in patients with HAS-BLED score ≥3, apixaban was associated with lower risk of major bleeding versus both dabigatran and rivaroxaban, while dabigatran was associated with lower risk versus rivaroxaban [26]. For stroke/SE, risk was broadly similar among all three DOACs [26].

Chao et al evaluated changes in HAS-BLED score among patients with NVAF who received anticoagulant therapy with either warfarin or DOACs, whereby patients who continued to receive anticoagulants after their score increased to ≥3 had lower risk of both major bleeding and ischemic stroke [27]. Current guidelines recommend the HAS-BLED score to assess bleeding risk; however, in the absence of absolute contraindications to oral anticoagulants, estimated bleeding risk alone should not guide treatment decisions when prescribing anticoagulation therapy [1, 2].

Polypharmacy, defined as the use of ≥5 concomitant medications, is frequent among patients with NVAF [28]. In a nationwide Belgian study, patients with polypharmacy who received apixaban were associated with lower risk of major bleeding versus VKAs, similar to the results of the current study for patients receiving concomitant antiplatelets, NSAIDs, or corticosteroids; however, patients who received dabigatran had similar risk versus VKAs [28]. Apixaban was the only DOAC to be associated with lower risk of GIB versus VKAs, whereas all DOACs were associated with lower risk of stroke/SE. In an analysis of the RE-LY trial, risk of major bleeding was lower with dabigatran use versus warfarin regardless of concomitant antiplatelet (aspirin or clopidogrel) use [29]. While dabigatran was more effective than warfarin in lowering risk of stroke/SE, this effect was attenuated somewhat by concomitant antiplatelet use [29]. Similarly, apixaban was associated with lower risk of major bleeding and stroke/SE versus warfarin among patients in the ARISTOTLE trial who received concomitant aspirin [30]. Results from both trials align with results seen here. Similarly, randomized trials on the use of DOACs with concomitant antiplatelets have been conducted in patients with AF who underwent percutaneous coronary intervention, and have reported favorable results with regimens containing apixaban (AUGUSTUS) [31], rivaroxaban (PIONEER AF-PCI) [32], and dabigatran (RE-DUAL-PCI) [33].

In randomized controlled trials of DOACs, patients with reduced kidney function (i.e., creatinine clearance <25–30 mL/min) were often excluded, and few post-marketing studies have had access to patient data on renal function. This lack of comparative data has resulted in uncertainty around the use of DOACs in patients with reduced kidney function [34]. The GLORIA-AF prospective registry study evaluated a subgroup of patients with NVAF who had CKD, defined as CHA_2_DS_2_-VASc score ≥2 and creatinine clearance <60 mL/min, and found that they were at significantly higher risk for thromboembolism versus other groups of patients with NVAF considered to have clinically complex disease [7]. In a subgroup of patients from the ARISTOPHANES study with renal disease (CKD stage 3–5), apixaban use was associated with lower risk of major bleeding versus warfarin [25]. For stroke/SE, apixaban and rivaroxaban use were associated with lower risk versus warfarin. When comparing DOACs individually, risk of major bleeding and GIB were lower with apixaban versus dabigatran and rivaroxaban. In the ARISTOTLE trial, patients with severe or moderate renal impairment who received apixaban had lower risk of major bleeding versus warfarin, whereas risk of stroke/SE was not significantly different [15]. Similarly, apixaban was associated with lower risk of major bleeding versus warfarin among patients with venous thromboembolism and CKD [35]. These data generally align with results of the current study, although comparison with dabigatran was not possible due to sample size.

### Strengths & limitations

This study was limited by its retrospective design; claims were used to determine risk factors for GIB which may be misclassified due to lack of information or data entry errors. It was also limited to patients in clinical practice in France; therefore, applicability to other populations with different demographic, racial, and/or socioeconomic characteristics cannot be established. The study uses the SNDS database which covers almost the entire French population, giving rich information on a range of data points as well as statistical power for robust comparative evidence. Using a single database that covers >99% of the patient population minimizes selection bias. Despite this large dataset, not all subgroup comparisons were possible due to sample size.

Because studies using real-world evidence are non-randomized, provider preferences can lead to selection bias and may result in imbalances between treatment groups. While residual confounding cannot be ruled out, we endeavored to address this imbalance by using PS matching to align the treatment cohorts using demographic and clinical characteristics. Because large datasets were used, very small departures from proportional hazards can be detected without an impact on the overall results; this was confirmed by AFT sensitivity analyses, which were similar to the main Cox proportional hazards analysis.

## Conclusions

Clinical complexity is common among patients with NVAF, which can pose challenges to treatment decision-making. DOACs were associated with improved safety and effectiveness when compared to VKAs among the key subgroups of NVAF patients at high risk of GIB. Apixaban was associated with lower risks of major bleeding, GIB, and stroke/SE versus rivaroxaban as well as lower risks of major bleeding, GIB and similar risk of stroke/SE versus dabigatran among several of these patient subgroups.

## Supporting information

S1 Table ICD-10 codes used to identify safety and effectiveness outcomes. (DOCX)

S2 Table Baseline characteristics prior to PS matching for patients age ≥75 years. (DOCX)

S3 Table Baseline characteristics prior to PS matching for patients with HAS-BLED score ≥3. (DOCX)

S4 Table Baseline characteristics prior to PS matching for patients receiving concomitant medication. (DOCX)

S5 Table Baseline characteristics prior to PS matching for patients with CKD stage 3 or 4. (DOCX)

S6 Table Estimated relative acceleration factors and 95% CI from the AFT analysis (PS matched population age ≥75 years). (DOCX)

S7 Table Estimated relative acceleration factors and 95% CI from the AFT analysis (PS matched population with HAS-BLED score ≥3). (DOCX)

S8 Table Estimated relative acceleration factors and 95% CI from the AFT analysis (PS matched population receiving concomitant medication). (DOCX)

S9 Table Estimated relative acceleration factors and 95% CI from the AFT analysis (PS matched population with CKD stage 3 or 4). (DOCX)
