# Supplementary material for: Comparative safety and effectiveness of oral anticoagulants in key subgroups of patients with non-valvular atrial fibrillation and at high risk of gastrointestinal bleeding: A cohort study based on the French National Health Data System (SNDS)
Source: PLoS One. 2025 Jan 22;20(1):e0317895. doi: 10.1371/journal.pone.0317895 (PMC11753696; doi:10.1371/journal.pone.0317895)
Supplement: S1 Table — (DOCX) [file pone.0317895.s001.docx]

**S1 Table**. ICD-10 codes used to identify safety and effectiveness outcomes

| MB, overall | Acute posthemorrhagic anemia: D62* – Intraocular bleeding Retinal hemorrhage: code H356 Vitreous hemorrhage: code H431 Vitreous hemorrhage in diseases classified elsewhere: code H450 – Otorrhagia: code H922 – Pericardic Hemopericardium, not elsewhere classified: code I312 – Respiratory bleeding Hemothorax: code J942 Hemorrhage from respiratory passages: R04*  Recurrent and persistent hematuria: code N02*  Unspecified hematuria: code R31*  Hemoperitoneum: code K661 – Intra articular bleeding Hemarthrosis: code M250 – Other bleeding Hemorrhage, not elsewhere classified: code R58* Traumatic secondary and recurrent hemorrhage: code T792  Intracranial hemorrhage: I60* to I62* Epidural hemorrhage: S064 Traumatic subdural hemorrhage: S065 Traumatic subarachnoid hemorrhage: S066  Oesophageal varices with bleeding: I850 Gastro‐oesophageal laceration‐hemorrhage syndrome: K226 Gastric ulcer/duodenal ulcer/peptic ulcer/gastrojejunal ulcer with hemorrhage: K250, K252, K254, K256, K260, K262, K264, K266, K270, K272, K274, K276, K280, K282, K284, K286 Acute hemorrhagic gastritis: K290 Hemorrhage of anus and rectum: K625 Hematemesis: K920 Melaena: K921; Gastrointestinal hemorrhage, unspecified: K922  Excessive and frequent menstruation with regular cycle N920  Ovulation bleeding N923  Postcoital and contact bleeding N930  Other specified abnormal uterine and vaginal bleeding N938  Abnormal uterine and vaginal bleeding, unspecified N939  Postmenopausal bleeding N950 |
| --- | --- |
| MB, GIB | Oesophageal varices with bleeding: I850 Gastro‐oesophageal laceration‐hemorrhage syndrome: K226 Gastric ulcer/duodenal ulcer/peptic ulcer/gastrojejunal ulcer with hemorrhage: K250, K252, K254, K256, K260, K262, K264, K266, K270, K272, K274, K276, K280, K282, K284, K286 Acute hemorrhagic gastritis: K290 Hemorrhage of anus and rectum: K625 Hematemesis: K920 Melaena: K921; Gastrointestinal hemorrhage, unspecified: K922 |
| MB, ICH | Intracranial hemorrhage: I60* to I62* Epidural hemorrhage: S064 Traumatic subdural hemorrhage: S065 Traumatic subarachnoid hemorrhage: S066 |
| MB, other sites | Excessive and frequent menstruation with regular cycle N920  Ovulation bleeding N923  Postcoital and contact bleeding N930  Other specified abnormal uterine and vaginal bleeding N938  Abnormal uterine and vaginal bleeding, unspecified N939  Postmenopausal bleeding N950 |
| Stroke (ischemic or hemorrhagic) | |
| Stroke (ischemic) | Cerebral infarction due to thrombosis of precerebral arteries I630  Cerebral infarction due to embolism of precerebral arteries I631  Cerebral infarction due to unspecified occlusion or stenosis of precerebral arteries I632  Cerebral infarction due to thrombosis of cerebral arteries I633  Cerebral infarction due to embolism of cerebral arteries I634  : Cerebral infarction due to unspecified occlusion or stenosis of cerebral arteries I635  Cerebral infarction due to cerebral venous thrombosis, nonpyogenic I636  Other cerebral infarction I638  Cerebral infarction, unspecified I639  Other cerebrovascular diseases I67* |
| Stroke (hemorrhagic) | Subarachnoid hemorrhage: I60*  Intracerebral hemorrhage I61* |
| Systemic embolism (SE) | Arterial embolism and thrombosis I74*  Atheroembolism I75* |
| Peripheral vascular disorders (non-stroke/SE peripheral vascular disease) | Atherosclerosis I70*  Aortic aneurysm and dissection I71*  Other aneurysm and dissections I72*  Other peripheral vascular diseases I73*  Other disorders of arteries and arterioles I77*  Diseases of capillaries I78*  Disorder of arteries, arterioles and capillaries in diseases classified elsewhere I79*  Chronic vascular disorder of intestine K551  Other vascular disorder of intestine K558  Vascular disorder of intestine, unspecified K559  Presence of other cardiac and vascular implants and grafts Z958  Presence of cardiac and vascular implants and grafts, unspecified Z959 |
