# Supplementary material for: Comparative safety and effectiveness of oral anticoagulants in key subgroups of patients with non-valvular atrial fibrillation and at high risk of gastrointestinal bleeding: A cohort study based on the French National Health Data System (SNDS)
Source: PLoS One. 2025 Jan 22;20(1):e0317895. doi: 10.1371/journal.pone.0317895 (PMC11753696; doi:10.1371/journal.pone.0317895)
Supplement: S2 Table — (DOCX) [file pone.0317895.s002.docx]

**S2 Table**. Baseline characteristics prior to PS matching for patients age ≥75 years

| **Characteristic** | | **Apixaban**  **(n = 120,696)** | **Rivaroxaban**  **(n = 57,020)** | **Dabigatran**  **(n = 11,286)** | **VKAs**  **(n = 37035)** |
| --- | --- | --- | --- | --- | --- |
| **Index dosage** | Standard dose | 55527 (46.01%) | 30358 (53.24%) | 2250 (19.94%) | - |
|  | Reduced dose | 65169 (53.99%) | 26662 (46.76%) | 9036 (80.06%) | - |
| **Atrial fibrillation identification setting** | Inpatient claim with I48 code | 81675 (67.67%) | 32959 (57.8%) | 6468 (57.31%) | 29186 (78.81%) |
|  | LTR registration with I48 code | 10109 (8.38%) | 6275 (11%) | 1309 (11.6%) | 1636 (4.42%) |
|  | Use of anti-arrhythmic drugs | 28912 (23.95%) | 17786 (31.19%) | 3509 (31.09%) | 6213 (16.78%) |
| **Follow up time (months), censored at switch, discontinuation, interruption, death, pregnancy, dialysis, CKD stage V, or end of follow up, mean (SD)** | | 13.4 [12.4] | 12.9 [12.6] | 13.3 [12.6] | 10.4 [11.1] |
| **Age at index date (years), mean (SD)** | | 84.5 [5.7] | 12.9 [12.6] | 83.3 [5.3] | 85.7 [5.8] |
|  | 75-79 years | 27567 (22.84%) | 17456 (30.61%) | 3182 (28.19%) | 6290 (16.98%) |
|  | ≥80 years | 93129 (77.16%) | 39564 (69.39%) | 8104 (71.81%) | 30745 (83.02%) |
| **Sex** | Male | 48389 (40.09%) | 25566 (44.84%) | 4999 (44.29%) | 15533 (41.94%) |
|  | Female | 72307 (59.91%) | 31454 (55.16%) | 6287 (55.71%) | 21502 (58.06%) |
| **GIB risk factors** | Age ≥75 years | 120696 (100%) | 57020 (100%) | 11286 (100%) | 37035 (100%) |
|  | HAS-BLED score, mean (SD) | 2.8 [1] | 2.6 [0.9] | 2.7 [0.9] | 3.1 [1.1] |
|  | 0 | - | - | - | - |
|  | 1 | 10114 (8.38%) | 6263 (10.98%) | 1158 (10.26%) | 1864 (5.03%) |
|  | 2 | 38732 (32.09%) | 19835 (34.79%) | 3751 (33.24%) | 8782 (23.71%) |
|  | ≥3 | 71850 (59.53%) | 30922 (54.23%) | 6377 (56.5%) | 26389 (71.25%) |
|  | Prior medications (antiplatelets, NSAIDs, or corticosteroids) | 63686 (52.77%) | 29996 (52.61%) | 5800 (51.39%) | 19430 (52.46%) |
|  | Renal impairment (CKD stage 3-4) | 6563 (5.44%) | 2111 (3.7%) | 319 (2.83%) | 6742 (18.2%) |
|  | Prior GI condition | 7910 (6.55%) | 3520 (6.17%) | 793 (7.03%) | 3081 (8.32%) |
| **Number of GIB risk factors** | 1 | 38189 (31.64%) | 20296 (35.59%) | 3876 (34.34%) | 8597 (23.21%) |
|  | 2 | 22669 (18.78%) | 9789 (17.17%) | 2107 (18.67%) | 6758 (18.25%) |
|  | 3 | 52535 (43.53%) | 24174 (42.4%) | 4752 (42.11%) | 16512 (44.58%) |
|  | 4 | 6942 (5.75%) | 2632 (4.62%) | 526 (4.66%) | 4812 (12.99%) |
|  | 5 | 361 (0.3%) | 129 (0.23%) | 25 (0.22%) | 356 (0.96%) |
| **Charlson Comorbidity Index score** | Mean (SD) | 1.7 [1.9] | 1.5 [1.8] | 1.5 [1.9] | 2.6 [2.3] |
|  | 0 | 35350 (29.29%) | 20558 (36.05%) | 4013 (35.56%) | 6233 (16.83%) |
|  | 1 or 2 | 53927 (44.68%) | 25065 (43.96%) | 4777 (42.33%) | 14560 (39.31%) |
|  | 3 or 4 | 21707 (17.98%) | 7934 (13.91%) | 1756 (15.56%) | 9869 (26.65%) |
|  | ≥5 | 9712 (8.05%) | 3463 (6.07%) | 740 (6.56%) | 6373 (17.21%) |
| **Comorbidities** | Myocardial infarction | 7761 (6.43%) | 3281 (5.75%) | 514 (4.55%) | 3668 (9.9%) |
|  | Congestive heart failure | 38306 (31.74%) | 15067 (26.42%) | 2610 (23.13%) | 17621 (47.58%) |
|  | Peripheral vascular disease | 9150 (7.58%) | 3697 (6.48%) | 2610 (23.13%) | 4607 (12.44%) |
|  | Cerebrovascular disease | 21602 (17.9%) | 6828 (11.97%) | 2135 (18.92%) | 6749 (18.22%) |
|  | Dementia | 12652 (10.48%) | 4873 (8.55%) | 806 (7.14%) | 5145 (13.89%) |
|  | Chronic pulmonary disease | 23687 (19.63%) | 11108 (19.48%) | 2097 (18.58%) | 8215 (22.18%) |
|  | Connective tissue disease | 1989 (1.65%) | 757 (1.33%) | 142 (1.26%) | 677 (1.83%) |
|  | Ulcer disease | 1067 (0.88%) | 360 (0.63%) | 101 (0.89%) | 562 (1.52%) |
|  | Mild liver disease | 1082 (0.9%) | 440 (0.77%) | 76 (0.67%) | 544 (1.47%) |
|  | Diabetes | 21528 (17.84%) | 10300 (18.06%) | 1867 (16.54%) | 8498 (22.95%) |
|  | Diabetes with end-organ damage | 1870 (1.55%) | 672 (1.18%) | 120 (1.06%) | 1645 (4.44%) |
|  | Hemiplegia | 8989 (7.45%) | 2398 (4.21%) | 954 (8.45%) | 2711 (7.32%) |
|  | Moderate or severe renal disease | 10609 (8.79%) | 3543 (6.21%) | 550 (4.87%) | 10175 (27.47%) |
|  | Any tumor (except for malignant neoplasm of skin) | 9255 (7.67%) | 4321 (7.58%) | 912 (8.08%) | 3595 (9.71%) |
|  | Metastatic solid tumor | 1521 (1.26%) | 784 (1.37%) | 178 (1.58%) | 634 (1.71%) |
|  | HIV/ AIDS | 34 (0.03%) | 13 (0.02%) | 4 (0.04%) | 22 (0.06%) |
|  | Moderate or severe liver disease | 196 (0.16%) | 63 (0.11%) | 18 (0.16%) | 134 (0.36%) |
|  | Hypertension | 102133 (84.62%) | 46610 (81.74%) | 9286 (82.28%) | 33219 (89.7%) |
|  | Diabetes mellitus | 23555 (19.52%) | 11142 (19.54%) | 2034 (18.02%) | 9391 (25.36%) |
|  | History of stroke, TIA, or VTE | 17015 (14.1%) | 4926 (8.64%) | 1716 (15.2%) | 4973 (13.43%) |
|  | Stroke or TIA | 16979 (14.07%) | 4899 (8.59%) | 1712 (15.17%) | 4952 (13.37%) |
|  | VTE | 40 (0.03%) | 30 (0.05%) | 4 (0.04%) | 25 (0.07%) |
|  | Vascular disease + peripheral vascular stenting | 22453 (18.6%) | 8784 (15.41%) | 1607 (14.24%) | 9928 (26.81%) |
|  | Peripheral vascular stenting | 573 (0.47%) | 284 (0.5%) | 44 (0.39%) | 219 (0.59%) |
|  | Anemia and coagulation defects | 14274 (11.83%) | 5262 (9.23%) | 1039 (9.21%) | 7719 (20.84%) |
|  | History of bleeding | 19176 (15.89%) | 7147 (12.53%) | 1524 (13.5%) | 9696 (26.18%) |
|  | Thrombocytopenia | 1116 (0.92%) | 452 (0.79%) | 103 (0.91%) | 581 (1.57%) |
|  | Atherosclerotic disease | 6348 (5.26%) | 2520 (4.42%) | 455 (4.03%) | 3289 (8.88%) |
|  | Vascular disease | 22452 (18.6%) | 8781 (15.4%) | 1607 (14.24%) | 9928 (26.81%) |
|  | Heart failure | 33785 (27.99%) | 13167 (23.09%) | 2252 (19.95%) | 15918 (42.98%) |
|  | Dyspepsia or stomach discomfort | 3120 (2.59%) | 1395 (2.45%) | 258 (2.29%) | 1059 (2.86%) |
|  | Coronary artery disease | 20139 (16.69%) | 8508 (14.92%) | 1461 (12.95%) | 9015 (24.34%) |
|  | Obesity (ICD-10 claims) | 10198 (8.45%) | 4785 (8.39%) | 871 (7.72%) | 4286 (11.57%) |
|  | Liver disease | 1162 (0.96%) | 463 (0.81%) | 81 (0.72%) | 582 (1.57%) |
|  | Chronic kidney disease | 10063 (8.34%) | 3320 (5.82%) | 515 (4.56%) | 9714 (26.23%) |
|  | Maximum stage 1 | 250 (0.21%) | 93 (0.16%) | 14 (0.12%) | 149 (0.4%) |
|  | Maximum stage 2 | 1120 (0.93%) | 433 (0.76%) | 78 (0.69%) | 497 (1.34%) |
|  | Maximum stage 3 | 5414 (4.49%) | 1828 (3.21%) | 281 (2.49%) | 3934 (10.62%) |
|  | Maximum stage 4 | 1149 (0.95%) | 283 (0.5%) | 38 (0.34%) | 2808 (7.58%) |
|  | Other/unknown | 2130 (1.76%) | 683 (1.2%) | 104 (0.92%) | 2326 (6.28%) |
|  | Chronic obstructive pulmonary disease | 432 (0.36%) | 192 (0.34%) | 42 (0.37%) | 233 (0.63%) |
|  | Hospitalization with alcohol discharge code | 1937 (1.6%) | 915 (1.6%) | 181 (1.6%) | 763 (2.06%) |
| **CHA_2_DS_2_-VASc score** | Mean (SD) | 4.4 [1.3] | 4.2 [1.2] | 4.2 [1.3] | 4.7 [1.3] |
|  | 2 | 5085 (4.21%) | 3607 (6.33%) | 619 (5.48%) | 841 (2.27%) |
|  | 3 | 22846 (18.93%) | 13553 (23.77%) | 2653 (23.51%) | 5002 (13.51%) |
|  | ≥4 | 92765 (76.86%) | 39860 (69.91%) | 8014 (71.01%) | 31192 (84.22%) |
| **Concomitant treatment** | Antiplatelets | 52128 (43.19%) | 24459 (42.9%) | 4742 (42.02%) | 16800 (45.36%) |
|  | Aromatase inhibitors | 924 (0.77%) | 450 (0.79%) | 76 (0.67%) | 287 (0.77%) |
|  | NSAIDs | 9317 (7.72%) | 4337 (7.61%) | 812 (7.19%) | 1574 (4.25%) |
|  | Corticosteroids | 15339 (12.71%) | 6686 (11.73%) | 1313 (11.63%) | 3792 (10.24%) |
|  | H2-receptor antagonists | 470 (0.39%) | 226 (0.4%) | 47 (0.42%) | 166 (0.45%) |
|  | Prostaglandins | 3566 (2.95%) | 1171 (2.05%) | 204 (1.81%) | 386 (1.04%) |
|  | Proton pump inhibitors | 55025 (45.59%) | 23966 (42.03%) | 4956 (43.91%) | 19365 (52.29%) |
|  | Anticonvulsant strong inhibitor of hepatic enzymes | 666 (0.55%) | 321 (0.56%) | 68 (0.6%) | 280 (0.76%) |
|  | HIV protease inhibitors | 634 (0.53%) | 191 (0.33%) | 34 (0.3%) | 54 (0.15%) |
|  | Strong inhibitors of both CYP3A4 and P-gp | 2376 (1.97%) | 713 (1.25%) | 168 (1.49%) | 337 (0.91%) |
|  | Statins | 18717 (15.51%) | 9060 (15.89%) | 1855 (16.44%) | 5794 (15.64%) |
|  | Selective estrogen receptor modulators | 220 (0.18%) | 120 (0.21%) | 23 (0.2%) | 67 (0.18%) |
|  | Serotonin reuptake inhibitors | 11140 (9.23%) | 4562 (8%) | 972 (8.61%) | 3624 (9.79%) |
|  | Sex hormones | 4790 (3.97%) | 1806 (3.17%) | 309 (2.74%) | 612 (1.65%) |
|  | Erythropoiesis stimulating agents | 647 (0.54%) | 232 (0.41%) | 30 (0.27%) | 888 (2.4%) |
|  | Beta blockers | 69682 (57.73%) | 31172 (54.67%) | 6062 (53.71%) | 22143 (59.79%) |
|  | Antiarrhythmic agents | 62730 (51.97%) | 33467 (58.69%) | 6591 (58.4%) | 16889 (45.6%) |

AIDS, acquired immunodeficiency syndrome; CKD, chronic kidney disease; CYP3A4, cytochrome P450 3A4; DOAC, direct oral anticoagulant; GIB, gastrointestinal bleed; HIV, human immunodeficiency virus; LTR, long-term recurrence; NSAID, nonsteroidal anti-inflammatory drug; P-gp, P-glycoprotein; PS, propensity score; SD, standard deviation; TIA, transient ischemic attack; VKA, vitamin K antagonist; VTE, venous thromboembolism.
