# Supplementary material for: Comparative safety and effectiveness of oral anticoagulants in key subgroups of patients with non-valvular atrial fibrillation and at high risk of gastrointestinal bleeding: A cohort study based on the French National Health Data System (SNDS)
Source: PLoS One. 2025 Jan 22;20(1):e0317895. doi: 10.1371/journal.pone.0317895 (PMC11753696; doi:10.1371/journal.pone.0317895)
Supplement: S3 Table — (DOCX) [file pone.0317895.s003.docx]

**S3 Table**. Baseline characteristics prior to PS matching for patients with HAS-BLED score ≥3.

| **Characteristic** | | **Apixaban**  **(n = 96,146)** | **Rivaroxaban**  **(n = 46,847)** | **Dabigatran**  **(n = 9,334)** | **VKAs**  **(n = 33,696)** |
| --- | --- | --- | --- | --- | --- |
| **Index dosage** | Standard dose | 51075 (53.12%) | 27389 (58.46%) | 2988 (32.01%) | - |
|  | Reduced dose | 45071 (46.88%) | 19458 (41.54%) | 6346 (67.99%) | - |
| **Atrial fibrillation identification setting** | Inpatient claim with I48 code | 69396 (72.18%) | 29365 (62.68%) | 6004 (64.32%) | 27957 (82.97%) |
|  | LTR registration with I48 code | 5606 (5.83%) | 3695 (7.89%) | 717 (7.68%) | 957 (2.84%) |
|  | Use of anti-arrhythmic drugs | 21144 (21.99%) | 13787 (29.43%) | 2613 (27.99%) | 4782 (14.19%) |
| **Follow up time (months), censored at switch, discontinuation, interruption, death, pregnancy, dialysis, CKD stage V, or end of follow up, mean (SD)** | | 13.7 [12.6] | 13.3 [12.7] | 13.8 [12.7] | 11.1 [11.5] |
| **Age at index date (years), mean (SD)** | | 80.8 [8.8] | 78.5 [8.7] | 78.9 [8.6] | 81.9 [9.3] |
|  | 18-54 years | 475 (0.49%) | 373 (0.8%) | 69 (0.74%) | 282 (0.84%) |
|  | 55-64 years | 2140 (2.23%) | 1370 (2.92%) | 263 (2.82%) | 1033 (3.07%) |
|  | 65-74 years | 21681 (22.55%) | 14182 (30.27%) | 2625 (28.12%) | 5992 (17.78%) |
|  | 75-79 years | 15002 (15.6%) | 8645 (18.45%) | 1693 (18.14%) | 4421 (13.12%) |
|  | ≥80 years | 56848 (59.13%) | 22277 (47.55%) | 4684 (50.18%) | 21968 (65.19%) |
| **Sex** | Male | 46800 (48.68%) | 25828 (55.13%) | 4974 (53.29%) | 17017 (50.50%) |
|  | Female | 49346 (51.32%) | 21019 (44.87%) | 4360 (46.71%) | 16679 (49.50%) |
| **GIB risk factors** | Age ≥75 years | 71850 (74.73%) | 30922 (66.01%) | 6377 (68.32%) | 26389 (78.31%) |
|  | HAS-BLED score, mean (SD) | 3.4 [0.6] | 3.3 [0.6] | 3.3 [0.6] | 3.6 [0.8] |
|  | 0 | - | - | - | - |
|  | 1 | - | - | - | - |
|  | 2 | - | - | - | - |
|  | ≥3 | 96146 (100%) | 46847 (100%) | 9334 (100%) | 33696 (100%) |
|  | Prior medications (antiplatelets, NSAIDs, or corticosteroids) | 74562 (77.55%) | 38780 (82.78%) | 7304 (78.25%) | 23299 (69.14%) |
|  | Renal impairment (CKD stage 3-4) | 7370 (7.67%) | 2563 (5.47%) | 380 (4.07%) | 7881 (23.39%) |
|  | Prior GI condition | 8047 (8.37%) | 3849 (8.22%) | 835 (8.95%) | 3440 (10.21%) |
| **Number of GIB risk factors** | 1 | 3696 (3.84%) | 1777 (3.79%) | 503 (5.39%) | 1279 (3.8%) |
|  | 2 | 30805 (32.04%) | 16944 (36.17%) | 3347 (35.86%) | 9451 (28.05%) |
|  | 3 | 54272 (56.45%) | 25337 (54.08%) | 4928 (52.8%) | 17696 (52.52%) |
|  | 4 | 7012 (7.29%) | 2660 (5.68%) | 531 (5.69%) | 4914 (14.58%) |
|  | 5 | 361 (0.38%) | 129 (0.28%) | 25 (0.27%) | 356 (1.06%) |
| **Charlson Comorbidity Index score** | Mean (SD) | 2.3 [2.1] | 2 [2.1] | 2.1 [2.1] | 3.3 [2.4] |
|  | 0 | 18373 (19.11%) | 11532 (24.62%) | 2164 (23.18%) | 3199 (9.49%) |
|  | 1 or 2 | 42206 (43.9%) | 21315 (45.5%) | 4041 (43.29%) | 11366 (33.73%) |
|  | 3 or 4 | 23686 (24.64%) | 9354 (19.97%) | 2126 (22.78%) | 10884 (32.3%) |
|  | ≥5 | 11881 (12.36%) | 4646 (9.92%) | 1003 (10.75%) | 8247 (24.47%) |
| **Comorbidities** | Myocardial infarction | 9521 (9.9%) | 4614 (9.85%) | 705 (7.55%) | 4734 (14.05%) |
|  | Congestive heart failure | 34039 (35.4%) | 14640 (31.25%) | 2504 (26.83%) | 17796 (52.81%) |
|  | Peripheral vascular disease | 11023 (11.46%) | 4996 (10.66%) | 930 (9.96%) | 5668 (16.82%) |
|  | Cerebrovascular disease | 25230 (26.24%) | 8562 (18.28%) | 2787 (29.86%) | 7992 (23.72%) |
|  | Dementia | 8706 (9.05%) | 3224 (6.88%) | 537 (5.75%) | 3824 (11.35%) |
|  | Chronic pulmonary disease | 20790 (21.62%) | 10348 (22.09%) | 1921 (20.58%) | 8224 (24.41%) |
|  | Connective tissue disease | 1642 (1.71%) | 643 (1.37%) | 122 (1.31%) | 659 (1.96%) |
|  | Ulcer disease | 1224 (1.27%) | 474 (1.01%) | 121 (1.3%) | 677 (2.01%) |
|  | Mild liver disease | 2159 (2.25%) | 1170 (2.5%) | 206 (2.21%) | 1253 (3.72%) |
|  | Diabetes | 23441 (24.38%) | 12180 (26%) | 2196 (23.53%) | 9963 (29.57%) |
|  | Diabetes with end-organ damage | 2698 (2.81%) | 1145 (2.44%) | 216 (2.31%) | 2441 (7.24%) |
|  | Hemiplegia | 10916 (11.35%) | 3115 (6.65%) | 1314 (14.08%) | 3344 (9.92%) |
|  | Moderate or severe renal disease | 12326 (12.82%) | 4493 (9.59%) | 704 (7.54%) | 12103 (35.92%) |
|  | Any tumor (except for malignant neoplasm of skin) | 8430 (8.77%) | 4106 (8.76%) | 868 (9.3%) | 3752 (11.13%) |
|  | Metastatic solid tumor | 1535 (1.6%) | 823 (1.76%) | 166 (1.78%) | 692 (2.05%) |
|  | HIV/ AIDS | 65 (0.07%) | 50 (0.11%) | 8 (0.09%) | 57 (0.17%) |
|  | Moderate or severe liver disease | 423 (0.44%) | 220 (0.47%) | 53 (0.57%) | 361 (1.07%) |
|  | Hypertension | 93463 (97.21%) | 45621 (97.38%) | 9043 (96.88%) | 32818 (97.39%) |
|  | Diabetes mellitus | 25567 (26.59%) | 13139 (28.05%) | 2409 (25.81%) | 11017 (32.7%) |
|  | History of stroke, TIA, or VTE | 20106 (20.91%) | 6184 (13.2%) | 2285 (24.48%) | 5912 (17.55%) |
|  | Stroke or TIA | 20066 (20.87%) | 6159 (13.15%) | 2282 (24.45%) | 5896 (17.5%) |
|  | VTE | 44 (0.05%) | 32 (0.07%) | 4 (0.04%) | 22 (0.07%) |
|  | Vascular disease + peripheral vascular stenting | 24356 (25.33%) | 10690 (22.82%) | 1930 (20.68%) | 11395 (33.82%) |
|  | Peripheral vascular stenting | 752 (0.78%) | 415 (0.89%) | 63 (0.67%) | 285 (0.85%) |
|  | Anemia and coagulation defects | 16786 (17.46%) | 6828 (14.58%) | 1320 (14.14%) | 9456 (28.06%) |
|  | History of bleeding | 23378 (24.32%) | 9610 (20.51%) | 2068 (22.16%) | 12016 (35.66%) |
|  | Thrombocytopenia | 1254 (1.3%) | 578 (1.23%) | 125 (1.34%) | 783 (2.32%) |
|  | Atherosclerotic disease | 7715 (8.02%) | 3398 (7.25%) | 597 (6.4%) | 4019 (11.93%) |
|  | Vascular disease | 24354 (25.33%) | 10686 (22.81%) | 1930 (20.68%) | 11393 (33.81%) |
|  | Heart failure | 28447 (29.59%) | 11918 (25.44%) | 1974 (21.15%) | 15534 (46.1%) |
|  | Dyspepsia or stomach discomfort | 2755 (2.87%) | 1347 (2.88%) | 239 (2.56%) | 1105 (3.28%) |
|  | Coronary artery disease | 23793 (24.75%) | 11386 (24.3%) | 1927 (20.64%) | 11182 (33.18%) |
|  | Obesity (ICD-10 claims) | 12550 (13.05%) | 6383 (13.63%) | 1257 (13.47%) | 5888 (17.47%) |
|  | Liver disease | 2299 (2.39%) | 1243 (2.65%) | 214 (2.29%) | 1327 (3.94%) |
|  | Chronic kidney disease | 11548 (12.01%) | 4132 (8.82%) | 647 (6.93%) | 11431 (33.92%) |
|  | Maximum stage 1 | 304 (0.32%) | 120 (0.26%) | 23 (0.25%) | 184 (0.55%) |
|  | Maximum stage 2 | 1327 (1.38%) | 534 (1.14%) | 104 (1.11%) | 600 (1.78%) |
|  | Maximum stage 3 | 6079 (6.32%) | 2224 (4.75%) | 333 (3.57%) | 4580 (13.59%) |
|  | Maximum stage 4 | 1291 (1.34%) | 339 (0.72%) | 47 (0.5%) | 3301 (9.8%) |
|  | Other/unknown | 2547 (2.65%) | 915 (1.95%) | 140 (1.5%) | 2766 (8.21%) |
|  | Chronic obstructive pulmonary disease | 358 (0.37%) | 181 (0.39%) | 37 (0.4%) | 224 (0.66%) |
|  | Hospitalization with alcohol discharge code | 4750 (4.94%) | 2808 (5.99%) | 524 (5.61%) | 1963 (5.83%) |
| **CHA_2_DS_2_-VASc score** | Mean (SD) | 4.5 [1.4] | 4.1 [1.3] | 4.3 [1.4] | 4.8 [1.4] |
|  | 0 | 14 (0.01%) | 18 (0.04%) | 3 (0.03%) | 7 (0.02%) |
|  | 1 | 418 (0.43%) | 321 (0.69%) | 49 (0.52%) | 163 (0.48%) |
|  | 2 | 5205 (5.41%) | 4160 (8.88%) | 668 (7.16%) | 1088 (3.23%) |
|  | 3 | 16915 (17.59%) | 11110 (23.72%) | 1969 (21.09%) | 4454 (13.22%) |
|  | ≥4 | 73594 (76.54%) | 31238 (66.68%) | 6645 (71.19%) | 27984 (83.05%) |
| **Concomitant treatment** | Antiplatelets | 65677 (68.31%) | 34192 (72.99%) | 6401 (68.58%) | 21085 (62.57%) |
|  | Aromatase inhibitors | 694 (0.72%) | 337 (0.72%) | 61 (0.65%) | 232 (0.69%) |
|  | NSAIDs | 12098 (12.58%) | 6318 (13.49%) | 1187 (12.72%) | 2088 (6.2%) |
|  | Corticosteroids | 13680 (14.23%) | 5991 (12.79%) | 1172 (12.56%) | 3758 (11.15%) |
|  | H2-receptor antagonists | 410 (0.43%) | 202 (0.43%) | 34 (0.36%) | 167 (0.5%) |
|  | Prostaglandins | 4464 (4.64%) | 1579 (3.37%) | 290 (3.11%) | 555 (1.65%) |
|  | Proton pump inhibitors | 51484 (53.55%) | 23922 (51.06%) | 4819 (51.63%) | 19930 (59.15%) |
|  | Anticonvulsant strong inhibitor of hepatic enzymes | 581 (0.6%) | 287 (0.61%) | 60 (0.64%) | 280 (0.83%) |
|  | HIV protease inhibitors | 738 (0.77%) | 226 (0.48%) | 43 (0.46%) | 104 (0.31%) |
|  | Strong inhibitors of both CYP3A4 and P-gp | 2617 (2.72%) | 825 (1.76%) | 183 (1.96%) | 417 (1.24%) |
|  | Statins | 19143 (19.91%) | 9851 (21.03%) | 1956 (20.96%) | 6099 (18.1%) |
|  | Selective estrogen receptor modulators | 133 (0.14%) | 79 (0.17%) | 17 (0.18%) | 43 (0.13%) |
|  | Serotonin reuptake inhibitors | 9262 (9.63%) | 3752 (8.01%) | 857 (9.18%) | 3274 (9.72%) |
|  | Sex hormones | 5315 (5.53%) | 2021 (4.31%) | 368 (3.94%) | 740 (2.2%) |
|  | Erythropoiesis stimulating agents | 687 (0.71%) | 251 (0.54%) | 36 (0.39%) | 1035 (3.07%) |
|  | Beta blockers | 61306 (63.76%) | 29333 (62.61%) | 5685 (60.91%) | 22021 (65.35%) |
|  | Antiarrhythmic agents | 48207 (50.14%) | 26947 (57.52%) | 5155 (55.23%) | 15121 (44.87%) |

AIDS, acquired immunodeficiency syndrome; CKD, chronic kidney disease; CYP3A4, cytochrome P450 3A4; DOAC, direct oral anticoagulant; GIB, gastrointestinal bleed; HIV, human immunodeficiency virus; LTR, long-term recurrence; NSAID, nonsteroidal anti-inflammatory drug; P-gp, P-glycoprotein; PS, propensity score; SD, standard deviation; TIA, transient ischemic attack; VKA, vitamin K antagonist; VTE, venous thromboembolism.
