# Supplementary material for: Comparative safety and effectiveness of oral anticoagulants in key subgroups of patients with non-valvular atrial fibrillation and at high risk of gastrointestinal bleeding: A cohort study based on the French National Health Data System (SNDS)
Source: PLoS One. 2025 Jan 22;20(1):e0317895. doi: 10.1371/journal.pone.0317895 (PMC11753696; doi:10.1371/journal.pone.0317895)
Supplement: S4 Table — (DOCX) [file pone.0317895.s004.docx]

**S4 Table**. Baseline characteristics prior to PS matching for patients receiving concomitant medication.

| **Characteristic** | | **Apixaban**  **(n = 98,803)** | **Rivaroxaban**  **(n = 57,580)** | **Dabigatran**  **(n = 10,145)** | **VKAs**  **(n = 27,340)** |
| --- | --- | --- | --- | --- | --- |
| **Index dosage** | Standard dose | 58359 (59.07%) | 37644 (65.38%) | 4013 (39.56%) | - |
|  | Reduced dose | 40444 (40.93%) | 19936 (34.62%) | 6132 (60.44%) | - |
| **Atrial fibrillation identification setting** | Inpatient claim with I48 code | 63046 (63.81%) | 31226 (54.23%) | 5459 (53.81%) | 20708 (75.74%) |
|  | LTR registration with I48 code | 8371 (8.47%) | 6237 (10.83%) | 1111 (10.95%) | 1168 (4.27%) |
|  | Use of anti-arrhythmic drugs | 27386 (27.72%) | 20117 (34.94%) | 3575 (35.24%) | 5464 (19.99%) |
| **Follow up time (months), censored at switch, discontinuation, interruption, death, pregnancy, dialysis, CKD stage V, or end of follow up, mean (SD)** | | 13.5 [12.7] | 13.1 [12.8] | 13.7 [12.9] | 11.1 [11.7] |
| **Age at index date (years), mean (SD)** | | 77.8 [11] | 74.2 [11.4] | 75.7 [10.8] | 79.6 [10.9] |
|  | 18-54 years | 2978 (3.01%) | 3111 (5.4%) | 351 (3.46%) | 689 (2.52%) |
|  | 55-64 years | 8777 (8.88%) | 7551 (13.11%) | 1111 (10.95%) | 2018 (7.38%) |
|  | 65-74 years | 23362 (23.65%) | 16922 (29.39%) | 2883 (28.42%) | 5203 (19.03%) |
|  | 75-79 years | 14508 (14.68%) | 9132 (15.86%) | 1610 (15.87%) | 3552 (12.99%) |
|  | ≥80 years | 49178 (49.77%) | 20864 (36.23%) | 4190 (41.30%) | 15878 (58.08%) |
| **Sex** | Male | 51926 (52.56%) | 34120 (59.26%) | 5775 (56.92%) | 14959 (54.71%) |
|  | Female | 46877 (47.44%) | 23460 (40.74%) | 4370 (43.08%) | 12381 (45.29%) |
| **GIB risk factors** | Age ≥75 years | 63686 (64.46%) | 29996 (52.09%) | 5800 (57.17%) | 19430 (71.07%) |
|  | HAS-BLED score, mean (SD) | 3 [1] | 2.8 [1] | 2.9 [1] | 3.4 [1] |
|  | 0 | 770 (0.78%) | 792 (1.38%) | 85 (0.84%) | 81 (0.3%) |
|  | 1 | 4981 (5.04%) | 4556 (7.91%) | 615 (6.06%) | 708 (2.59%) |
|  | 2 | 18490 (18.71%) | 13452 (23.36%) | 2141 (21.1%) | 3252 (11.89%) |
|  | ≥3 | 74562 (75.47%) | 38780 (67.35%) | 7304 (72%) | 23299 (85.22%) |
|  | Prior medications (antiplatelets, NSAIDs, or corticosteroids) | 98803 (100%) | 57580 (100%) | 10145 (100%) | 27340 (100%) |
|  | Renal impairment (CKD stage 3-4) | 4306 (4.36%) | 1511 (2.62%) | 228 (2.25%) | 4634 (16.95%) |
|  | Prior GI condition | 6423 (6.5%) | 3522 (6.12%) | 697 (6.87%) | 2093 (7.66%) |
| **Number of GIB risk factors** | 1 | 14716 (14.89%) | 13358 (23.2%) | 1896 (18.69%) | 2349 (8.59%) |
|  | 2 | 26647 (26.97%) | 17461 (30.32%) | 3031 (29.88%) | 5779 (21.14%) |
|  | 3 | 50351 (50.96%) | 24064 (41.79%) | 4681 (46.14%) | 14315 (52.36%) |
|  | 4 | 6728 (6.81%) | 2568 (4.46%) | 512 (5.05%) | 4541 (16.61%) |
|  | 5 | 361 (0.37%) | 129 (0.22%) | 25 (0.25%) | 356 (1.3%) |
| **Charlson Comorbidity Index score** | Mean (SD) | 1.8 [2.0] | 1.5 [1.9] | 1.6 [1.9] | 2.8 [2.5] |
|  | 0 | 27668 (28%) | 20106 (34.92%) | 3401 (33.52%) | 3990 (14.59%) |
|  | 1 or 2 | 45814 (46.37%) | 26100 (45.33%) | 4542 (44.77%) | 10656 (38.98%) |
|  | 3 or 4 | 17095 (17.3%) | 7794 (13.54%) | 1494 (14.73%) | 7217 (26.4%) |
|  | ≥5 | 8226 (8.33%) | 3580 (6.22%) | 708 (6.98%) | 5477 (20.03%) |
| **Comorbidities** | Myocardial infarction | 10291 (10.42%) | 5596 (9.72%) | 778 (7.67%) | 4694 (17.17%) |
|  | Congestive heart failure | 30930 (31.3%) | 14571 (25.31%) | 2374 (23.4%) | 13580 (49.67%) |
|  | Peripheral vascular disease | 10069 (10.19%) | 5061 (8.79%) | 838 (8.26%) | 4792 (17.53%) |
|  | Cerebrovascular disease | 16071 (16.27%) | 6234 (10.83%) | 1757 (17.32%) | 4970 (18.18%) |
|  | Dementia | 6157 (6.23%) | 2367 (4.11%) | 401 (3.95%) | 2267 (8.29%) |
|  | Chronic pulmonary disease | 22144 (22.41%) | 12601 (21.88%) | 2109 (20.79%) | 6863 (25.1%) |
|  | Connective tissue disease | 2102 (2.13%) | 938 (1.63%) | 155 (1.53%) | 682 (2.49%) |
|  | Ulcer disease | 804 (0.81%) | 331 (0.57%) | 76 (0.75%) | 365 (1.34%) |
|  | Mild liver disease | 1209 (1.22%) | 660 (1.15%) | 107 (1.05%) | 607 (2.22%) |
|  | Diabetes | 22103 (22.37%) | 12949 (22.49%) | 2215 (21.83%) | 7975 (29.17%) |
|  | Diabetes with end-organ damage | 1895 (1.92%) | 889 (1.54%) | 155 (1.53%) | 1739 (6.36%) |
|  | Hemiplegia | 5511 (5.58%) | 1836 (3.19%) | 673 (6.63%) | 1711 (6.26%) |
|  | Moderate or severe renal disease | 7249 (7.34%) | 2718 (4.72%) | 422 (4.16%) | 7201 (26.34%) |
|  | Any tumor (except for malignant neoplasm of skin) | 7358 (7.45%) | 4086 (7.1%) | 779 (7.68%) | 2650 (9.69%) |
|  | Metastatic solid tumor | 1446 (1.46%) | 845 (1.47%) | 172 (1.7%) | 537 (1.96%) |
|  | HIV/ AIDS | 66 (0.07%) | 60 (0.1%) | 9 (0.09%) | 58 (0.21%) |
|  | Moderate or severe liver disease | 217 (0.22%) | 106 (0.18%) | 24 (0.24%) | 159 (0.58%) |
|  | Hypertension | 84445 (85.47%) | 46906 (81.46%) | 8436 (83.15%) | 24960 (91.29%) |
|  | Diabetes mellitus | 23883 (24.17%) | 13854 (24.06%) | 2368 (23.34%) | 8712 (31.87%) |
|  | History of stroke, TIA, or VTE | 11889 (12.03%) | 4143 (7.2%) | 1341 (13.22%) | 3376 (12.35%) |
|  | Stroke or TIA | 11866 (12.01%) | 4125 (7.16%) | 1337 (13.18%) | 3364 (12.3%) |
|  | VTE | 25 (0.03%) | 21 (0.04%) | 4 (0.04%) | 15 (0.05%) |
|  | Vascular disease + peripheral vascular stenting | 22437 (22.71%) | 11163 (19.39%) | 1795 (17.69%) | 9671 (35.37%) |
|  | Peripheral vascular stenting | 798 (0.81%) | 460 (0.8%) | 64 (0.63%) | 277 (1.01%) |
|  | Anemia and coagulation defects | 9496 (9.61%) | 4018 (6.98%) | 773 (7.62%) | 5271 (19.28%) |
|  | History of bleeding | 13133 (13.29%) | 5691 (9.88%) | 1168 (11.51%) | 6687 (24.46%) |
|  | Thrombocytopenia | 866 (0.88%) | 417 (0.72%) | 85 (0.84%) | 486 (1.78%) |
|  | Atherosclerotic disease | 6919 (7%) | 3353 (5.82%) | 534 (5.26%) | 3322 (12.15%) |
|  | Vascular disease | 22434 (22.71%) | 11159 (19.38%) | 1795 (17.69%) | 9668 (35.36%) |
|  | Heart failure | 25242 (25.55%) | 11535 (20.03%) | 1850 (18.24%) | 11436 (41.83%) |
|  | Dyspepsia or stomach discomfort | 2493 (2.52%) | 1390 (2.41%) | 247 (2.43%) | 769 (2.81%) |
|  | Coronary artery disease | 24490 (24.79%) | 12933 (22.46%) | 2031 (20.02%) | 10292 (37.64%) |
|  | Obesity (ICD-10 claims) | 11554 (11.69%) | 6863 (11.92%) | 1213 (11.96%) | 4611 (16.87%) |
|  | Liver disease | 1293 (1.31%) | 701 (1.22%) | 114 (1.12%) | 649 (2.37%) |
|  | Chronic kidney disease | 6777 (6.86%) | 2496 (4.33%) | 392 (3.86%) | 6769 (24.76%) |
|  | Maximum stage 1 | 168 (0.17%) | 77 (0.13%) | 15 (0.15%) | 109 (0.4%) |
|  | Maximum stage 2 | 720 (0.73%) | 311 (0.54%) | 61 (0.6%) | 329 (1.2%) |
|  | Maximum stage 3 | 3553 (3.6%) | 1307 (2.27%) | 198 (1.95%) | 2703 (9.89%) |
|  | Maximum stage 4 | 753 (0.76%) | 204 (0.35%) | 30 (0.3%) | 1931 (7.06%) |
|  | Other/unknown | 1583 (1.6%) | 597 (1.04%) | 88 (0.87%) | 1697 (6.21%) |
|  | Chronic obstructive pulmonary disease | 328 (0.33%) | 175 (0.3%) | 36 (0.35%) | 170 (0.62%) |
|  | Hospitalization with alcohol discharge code | 2815 (2.85%) | 1742 (3.03%) | 292 (2.88%) | 1058 (3.87%) |
| **CHA_2_DS_2_-VASc score** | Mean (SD) | 3.9 [1.6] | 3.4 [1.6] | 3.6 [1.6] | 4.4 [1.5] |
|  | 0 | 1655 (1.68%) | 2025 (3.52%) | 232 (2.29%) | 167 (0.61%) |
|  | 1 | 4984 (5.04%) | 4968 (8.63%) | 683 (6.73%) | 702 (2.57%) |
|  | 2 | 11551 (11.69%) | 9298 (16.15%) | 1476 (14.55%) | 1860 (6.8%) |
|  | 3 | 20596 (20.85%) | 13749 (23.88%) | 2360 (23.26%) | 4453 (16.29%) |
|  | ≥4 | 60017 (60.74%) | 27540 (47.83%) | 5394 (53.17%) | 20158 (73.73%) |
| **Concomitant treatment** | Antiplatelets | 77045 (77.98%) | 43616 (75.75%) | 7830 (77.18%) | 23268 (85.11%) |
|  | Aromatase inhibitors | 619 (0.63%) | 330 (0.57%) | 58 (0.57%) | 171 (0.63%) |
|  | NSAIDs | 17723 (17.94%) | 11130 (19.33%) | 1876 (18.49%) | 2666 (9.75%) |
|  | Corticosteroids | 24687 (24.99%) | 13377 (23.23%) | 2272 (22.4%) | 5523 (20.2%) |
|  | H2-receptor antagonists | 447 (0.45%) | 264 (0.46%) | 45 (0.44%) | 154 (0.56%) |
|  | Prostaglandins | 5663 (5.73%) | 2320 (4.03%) | 357 (3.52%) | 630 (2.3%) |
|  | Proton pump inhibitors | 52362 (53%) | 28443 (49.4%) | 5185 (51.11%) | 17097 (62.53%) |
|  | Anticonvulsant strong inhibitor of hepatic enzymes | 557 (0.56%) | 332 (0.58%) | 60 (0.59%) | 231 (0.84%) |
|  | HIV protease inhibitors | 1045 (1.06%) | 349 (0.61%) | 54 (0.53%) | 138 (0.5%) |
|  | Strong inhibitors of both CYP3A4 and P-gp | 3502 (3.54%) | 1272 (2.21%) | 229 (2.26%) | 453 (1.66%) |
|  | Statins | 20046 (20.29%) | 11474 (19.93%) | 2120 (20.9%) | 5623 (20.57%) |
|  | Selective estrogen receptor modulators | 156 (0.16%) | 99 (0.17%) | 19 (0.19%) | 40 (0.15%) |
|  | Serotonin reuptake inhibitors | 7876 (7.97%) | 3870 (6.72%) | 762 (7.51%) | 2401 (8.78%) |
|  | Sex hormones | 6788 (6.87%) | 3074 (5.34%) | 471 (4.64%) | 829 (3.03%) |
|  | Erythropoiesis stimulating agents | 533 (0.54%) | 215 (0.37%) | 29 (0.29%) | 734 (2.68%) |
|  | Beta blockers | 61866 (62.62%) | 34596 (60.08%) | 5973 (58.88%) | 18066 (66.08%) |
|  | Antiarrhythmic agents | 56666 (57.35%) | 36830 (63.96%) | 6460 (63.68%) | 14071 (51.47%) |

AIDS, acquired immunodeficiency syndrome; CKD, chronic kidney disease; CYP3A4, cytochrome P450 3A4; DOAC, direct oral anticoagulant; GIB, gastrointestinal bleed; HIV, human immunodeficiency virus; LTR, long-term recurrence; NSAID, nonsteroidal anti-inflammatory drug; P-gp, P-glycoprotein; PS, propensity score; SD, standard deviation; TIA, transient ischemic attack; VKA, vitamin K antagonist; VTE, venous thromboembolism.
