# Supplementary material for: Comparative safety and effectiveness of oral anticoagulants in key subgroups of patients with non-valvular atrial fibrillation and at high risk of gastrointestinal bleeding: A cohort study based on the French National Health Data System (SNDS)
Source: PLoS One. 2025 Jan 22;20(1):e0317895. doi: 10.1371/journal.pone.0317895 (PMC11753696; doi:10.1371/journal.pone.0317895)
Supplement: S5 Table — (DOCX) [file pone.0317895.s005.docx]

**S5 Table**. Baseline characteristics prior to PS matching for patients with CKD stage 3 or 4.

| **Characteristic** | | **Apixaban**  **(n = 7,594)** | **Rivaroxaban**  **(n = 2,656)** | **VKAs**  **(n = 8,058)** |
| --- | --- | --- | --- | --- |
| **Index dosage** | Standard dose | 2091 (27.53%) | 743 (27.97%) | - |
|  | Reduced dose | 5503 (72.47%) | 1913 (72.03%) | - |
| **Atrial fibrillation identification setting** | Inpatient claim with I48 code | 6943 (91.43%) | 2340 (88.1%) | 7473 (92.74%) |
|  | LTR registration with I48 code | 73 (0.96%) | 36 (1.36%) | 44 (0.55%) |
|  | Use of anti-arrhythmic drugs | 578 (7.61%) | 280 (10.54%) | 541 (6.71%) |
| **Follow up time (months), censored at switch, discontinuation, interruption, death, pregnancy, dialysis, CKD stage V, or end of follow up, mean (SD)** | | 10.4 [10.4] | 10.5 [10.7] | 10.1 [10.5] |
| **Age at index date (years), mean (SD)** | | 84.3 [8.7] | 82.2 [9.4] | 83.4 [9] |
|  | 18-54 years | 30 (0.4%) | 21 (0.79%) | 40 (0.5%) |
|  | 55-64 years | 196 (2.58%) | 103 (3.88%) | 251 (3.11%) |
|  | 65-74 years | 805 (10.6%) | 421 (15.85%) | 1025 (12.72%) |
|  | 75-79 years | 766 (10.09%) | 362 (13.63%) | 910 (11.29%) |
|  | ≥80 years | 5797 (76.34%) | 1749 (65.85%) | 5832 (72.38%) |
| **Sex** | Male | 3334 (43.90%) | 1331 (50.11%) | 4081 (50.64%) |
|  | Female | 4260 (56.1%) | 1325 (49.89%) | 3977 (49.35%) |
| **GIB risk factors** | Age ≥75 years | 6563 (86.42%) | 2111 (79.48%) | 6742 (83.67%) |
|  | HAS-BLED score, mean (SD) | 4 [0.9] | 4 [0.9] | 4.1 [0.9] |
|  | 0 | 0 (0.0%) | 0 (0.0%) | 0 (0.0%) |
|  | 1 | 10 (0.13%) | 5 (0.19%) | 2 (0.02%) |
|  | 2 | 214 (2.82%) | 88 (3.31%) | 175 (2.17%) |
|  | ≥3 | 7370 (97.05%) | 2563 (96.5%) | 7881 (97.8%) |
|  | Prior medications (antiplatelets, NSAIDs, or corticosteroids) | 4306 (56.7%) | 1511 (56.89%) | 4634 (57.51%) |
|  | Renal impairment (CKD stage 3-4) | 7594 (100%) | 2656 (100%) | 8058 (100%) |
|  | Prior GI condition | 757 (9.97%) | 271 (10.2%) | 901 (11.18%) |
| **Number of GIB risk factors** | 1 | 66 (0.87%) | 36 (1.36%) | 57 (0.71%) |
|  | 2 | 404 (5.32%) | 208 (7.83%) | 401 (4.98%) |
|  | 3 | 3141 (41.36%) | 1117 (42.06%) | 3399 (42.18%) |
|  | 4 | 3622 (47.7%) | 1166 (43.9%) | 3845 (47.72%) |
|  | 5 | 361 (4.75%) | 129 (4.86%) | 356 (4.42%) |
| **Charlson Comorbidity Index score** | Mean (SD) | 4.7 [2.1] | 4.7 [2.2] | 5.1 [2.3] |
|  | 0 | 0 (0.0%) | 0 (0.0%) | 0 (0.0%) |
|  | 1 or 2 | 602 (7.93%) | 257 (9.68%) | 509 (6.32%) |
|  | 3 or 4 | 3560 (46.88%) | 1264 (47.59%) | 3484 (43.24%) |
|  | ≥5 | 3432 (45.19%) | 1135 (42.73%) | 4065 (50.45%) |
| **Comorbidities** | Myocardial infarction | 950 (12.51%) | 312 (11.75%) | 1234 (15.31%) |
|  | Congestive heart failure | 4601 (60.59%) | 1542 (58.06%) | 5544 (68.8%) |
|  | Peripheral vascular disease | 1351 (17.79%) | 453 (17.06%) | 1802 (22.36%) |
|  | Cerebrovascular disease | 1838 (24.2%) | 507 (19.09%) | 1654 (20.53%) |
|  | Dementia | 1385 (18.24%) | 462 (17.39%) | 1228 (15.24%) |
|  | Chronic pulmonary disease | 2003 (26.38%) | 749 (28.2%) | 2266 (28.12%) |
|  | Connective tissue disease | 240 (3.16%) | 59 (2.22%) | 174 (2.16%) |
|  | Ulcer disease | 123 (1.62%) | 44 (1.66%) | 174 (2.16%) |
|  | Mild liver disease | 179 (2.36%) | 80 (3.01%) | 233 (2.89%) |
|  | Diabetes | 2392 (31.5%) | 875 (32.94%) | 3024 (37.53%) |
|  | Diabetes with end-organ damage | 887 (11.68%) | 315 (11.86%) | 1452 (18.02%) |
|  | Hemiplegia | 702 (9.24%) | 177 (6.66%) | 632 (7.84%) |
|  | Moderate or severe renal disease | 7594 (100%) | 2656 (100%) | 8058 (100%) |
|  | Any tumor (except for malignant neoplasm of skin) | 868 (11.43%) | 305 (11.48%) | 1052 (13.06%) |
|  | Metastatic solid tumor | 122 (1.61%) | 61 (2.3%) | 143 (1.77%) |
|  | HIV/ AIDS | 11 (0.14%) | 7 (0.26%) | 8 (0.1%) |
|  | Moderate or severe liver disease | 25 (0.33%) | 8 (0.3%) | 44 (0.55%) |
|  | Hypertension | 7193 (94.72%) | 2493 (93.86%) | 7803 (96.84%) |
|  | Diabetes mellitus | 2654 (34.95%) | 959 (36.11%) | 3338 (41.42%) |
|  | History of stroke, TIA, or VTE | 1388 (18.28%) | 338 (12.73%) | 1122 (13.92%) |
|  | Stroke or TIA | 1381 (18.19%) | 334 (12.58%) | 1118 (13.87%) |
|  | VTE | 9 (0.12%) | 4 (0.15%) | 4 (0.05%) |
|  | Vascular disease + peripheral vascular stenting | 2737 (36.04%) | 919 (34.6%) | 3213 (39.87%) |
|  | Peripheral vascular stenting | 66 (0.87%) | 23 (0.87%) | 89 (1.1%) |
|  | Anemia and coagulation defects | 2577 (33.93%) | 805 (30.31%) | 3092 (38.37%) |
|  | History of bleeding | 2977 (39.2%) | 959 (36.11%) | 3543 (43.97%) |
|  | Thrombocytopenia | 178 (2.34%) | 64 (2.41%) | 210 (2.61%) |
|  | Atherosclerotic disease | 989 (13.02%) | 341 (12.84%) | 1382 (17.15%) |
|  | Vascular disease | 2737 (36.04%) | 919 (34.6%) | 3212 (39.86%) |
|  | Heart failure | 4182 (55.07%) | 1363 (51.32%) | 5079 (63.03%) |
|  | Dyspepsia or stomach discomfort | 315 (4.15%) | 121 (4.56%) | 350 (4.34%) |
|  | Coronary artery disease | 2468 (32.5%) | 871 (32.79%) | 3054 (37.9%) |
|  | Obesity (ICD-10 claims) | 1570 (20.67%) | 593 (22.33%) | 1859 (23.07%) |
|  | Liver disease | 189 (2.49%) | 83 (3.13%) | 247 (3.07%) |
|  | Chronic kidney disease | 7594 (100%) | 2656 (100%) | 8058 (100%) |
|  | Maximum stage 1 | - | - | - |
|  | Maximum stage 2 | - | - | - |
|  | Maximum stage 3 | 6280 (82.7%) | 2309 (86.94%) | 4699 (58.31%) |
|  | Maximum stage 4 | 1314 (17.3%) | 347 (13.06%) | 3359 (41.69%) |
|  | Other/unknown | - | - | - |
|  | Chronic obstructive pulmonary disease | 43 (0.57%) | 13 (0.49%) | 59 (0.73%) |
|  | Hospitalization with alcohol discharge code | 271 (3.57%) | 151 (5.69%) | 314 (3.9%) |
| **CHA_2_DS_2_-VASc score** | Mean (SD) | 5 [1.4] | 4.7 [1.4] | 5 [1.3] |
|  | 0 | 4 (0.05%) | 4 (0.15%) | 4 (0.05%) |
|  | 1 | 43 (0.57%) | 24 (0.9%) | 33 (0.41%) |
|  | 2 | 163 (2.15%) | 103 (3.88%) | 161 (2%) |
|  | 3 | 650 (8.56%) | 329 (12.39%) | 661 (8.2%) |
|  | ≥4 | 6734 (88.68%) | 2196 (82.68%) | 7199 (89.34%) |
| **Concomitant treatment** | Antiplatelets | 3632 (47.83%) | 1280 (48.19%) | 4066 (50.46%) |
|  | Aromatase inhibitors | 69 (0.91%) | 15 (0.56%) | 58 (0.72%) |
|  | NSAIDs | 515 (6.78%) | 172 (6.48%) | 341 (4.23%) |
|  | Corticosteroids | 1191 (15.68%) | 395 (14.87%) | 1009 (12.52%) |
|  | H2-receptor antagonists | 27 (0.36%) | 12 (0.45%) | 37 (0.46%) |
|  | Prostaglandins | 316 (4.16%) | 94 (3.54%) | 184 (2.28%) |
|  | Proton pump inhibitors | 4251 (55.98%) | 1451 (54.63%) | 4732 (58.72%) |
|  | Anticonvulsant strong inhibitor of hepatic enzymes | 48 (0.63%) | 21 (0.79%) | 50 (0.62%) |
|  | HIV protease inhibitors | 49 (0.65%) | 15 (0.56%) | 34 (0.42%) |
|  | Strong inhibitors of both CYP3A4 and P-gp | 267 (3.52%) | 52 (1.96%) | 139 (1.72%) |
|  | Statins | 1180 (15.54%) | 451 (16.98%) | 1301 (16.15%) |
|  | Selective estrogen receptor modulators | 7 (0.09%) | 5 (0.19%) | 6 (0.07%) |
|  | Serotonin reuptake inhibitors | 812 (10.69%) | 283 (10.66%) | 780 (9.68%) |
|  | Sex hormones | 378 (4.98%) | 122 (4.59%) | 218 (2.71%) |
|  | Erythropoiesis stimulating agents | 185 (2.44%) | 48 (1.81%) | 603 (7.48%) |
|  | Beta blockers | 4916 (64.74%) | 1743 (65.63%) | 5368 (66.62%) |
|  | Antiarrhythmic agents | 2987 (39.33%) | 1155 (43.49%) | 3212 (39.86%) |

AIDS, acquired immunodeficiency syndrome; CKD, chronic kidney disease; CYP3A4, cytochrome P450 3A4; DOAC, direct oral anticoagulant; GIB, gastrointestinal bleed; HIV, human immunodeficiency virus; LTR, long-term recurrence; NSAID, nonsteroidal anti-inflammatory drug; P-gp, P-glycoprotein; PS, propensity score; SD, standard deviation; TIA, transient ischemic attack; VKA, vitamin K antagonist; VTE, venous thromboembolism.
