# Supplementary material for: Comparative safety and effectiveness of oral anticoagulants in key subgroups of patients with non-valvular atrial fibrillation and at high risk of gastrointestinal bleeding: A cohort study based on the French National Health Data System (SNDS)
Source: PLoS One. 2025 Jan 22;20(1):e0317895. doi: 10.1371/journal.pone.0317895 (PMC11753696; doi:10.1371/journal.pone.0317895)
Supplement: S6 Table — (DOCX) [file pone.0317895.s006.docx]

**S6 Table.** Estimated relative acceleration factors and 95% CI from the AFT analysis (PS matched population age ≥75 years)

|  | **Apixaban vs VKAs**  **(n = 34,007)** | **Dabigatran vs VKAs (n = 11,278)** | **Rivaroxaban vs VKAs (n = 28,318)** | **Apixaban vs dabigatran**  **(n = 11,249)** | **Dabigatran vs rivaroxaban**  **(n = 11,284)** | **Apixaban vs rivaroxaban**  **(n = 57,612)** |
| --- | --- | --- | --- | --- | --- | --- |
| **Major bleed** | 0.448(0.407;0.493)  *p*<0.0001 | 0.752(0.608;0.93)  *p*<0.01 | 0.826(0.743;0.919)  *p*<0.001 | 0.553(0.443;0.691)  *p*<0.0001 | 0.704(0.578;0.857)  *p*<0.001 | 0.492(0.449;0.539)  *p*<0.0001 |
| **GIB** | 0.435(0.368;0.514)  *p*<0.0001 | 0.468(0.333;0.657)  *p*<0.0001 | 1.029(0.861;1.229)  *p*=0.7554 | 0.292(0.205;0.414)  *p*<0.0001 | 1.308(0.971;1.761)  *p*=0.0772 | 0.374(0.319;0.44)  *p*<0.0001 |
| **ICH** | 0.493(0.416;0.585)  *p*<0.0001 | 1.526(0.902;2.583)  *p*=0.1155 | 0.584(0.492;0.694)  *p*<0.0001 | 1.386(0.892;2.155)  *p*=0.1466 | 0.484(0.314;0.746)  *p*=0.0010 | 0.898(0.769;1.048)  *p*=0.1724 |
| **Other bleed** | 0.416(0.356;0.487)  *p*<0.0001 | 0.899(0.628;1.288)  *p*=0.5632 | 0.855(0.724;1.01)  *p*=0.0659 | 0.727(0.518;1.021)  *p*<0.0661 | 0.439(0.323;0.595)  *p*<0.0001 | 0.427(0.371;0.492)  *p*<0.0001 |
| **Stroke/SE** | 0.681(0.608;0.764)  *p*<0.0001 | 0.936(0.701;1.25)  *p*=0.6528 | 0.695(0.613;0.787)  *p*<0.0001 | 0.865(0.665;1.124)  *p*=0.2770 | 0.85(0.67;1.079)  *p*=0.1812 | 0.846(0.762;0.939)  *p*<0.01 |
| **SE** | 0.748(0.606;0.923)  *p*<0.01 | 1.178(0.736;1.887)  *p*=0.4943 | 0.725(0.567;0.925)  *p*<0.0001 | 0.779(0.441;1.375)  *p*=0.3888 | 0.67(0.4;1.121)  *p*=0.1271 | 0.863(0.679;1.097)  *p*=0.2282 |
| **Stroke (ischemic or hemorrhagic)** | 0.656(0.573;0.75)  *p*<0.0001 | 0.854(0.615;1.187)  *p*=0.3478 | 0.681(0.589;0.787)  *p*<0.01 | 0.888(0.663;1.19)  *p*=0.4269 | 0.909(0.695;1.189)  *p*=0.4867 | 0.836(0.743;0.94)  *p*<0.01 |
| **Ischemic stroke** | 0.805(0.684;0.947)  *p*<0.01 | 0.816(0.558;1.192)  *p*=0.2929 | 0.738(0.615;0.886)  *p*<0.01 | 0.834(0.59;1.179)  *p*=0.3035 | 1.038(0.754;1.428)  *p*=0.8200 | 0.866(0.75;1.001)  *p*=0.0518 |
| **Hemorrhagic stroke** | 0.388(0.303;0.498)  *p*<0.0001 | 0.875(0.455;1.683)  *p*=0.6884 | 0.55(0.43;0.703)  *p*<0.0001 | 0.931(0.544;1.593)  *p*=0.7936 | 0.733(0.478;1.123)  *p*=0.1536 | 0.773(0.63;0.949)  *p*=0.0140 |

AFT, accelerated failure time; CI, confidence interval; GIB, gastrointestinal bleeding; ICH, intracranial hemorrhage; PS, propensity score; SE, systemic embolism; VKA, vitamin K antagonist.
