# Supplementary material for: Comparative safety and effectiveness of oral anticoagulants in key subgroups of patients with non-valvular atrial fibrillation and at high risk of gastrointestinal bleeding: A cohort study based on the French National Health Data System (SNDS)
Source: PLoS One. 2025 Jan 22;20(1):e0317895. doi: 10.1371/journal.pone.0317895 (PMC11753696; doi:10.1371/journal.pone.0317895)
Supplement: S7 Table — (DOCX) [file pone.0317895.s007.docx]

**S7 Table.** Estimated relative acceleration factors and 95% CI from the AFT analysis (PS matched population with HAS-BLED score ≥3)

|  | **Apixaban vs VKAs**  **(n = 31,652)** | **Dabigatran vs VKAs (n = 9,331)** | **Rivaroxaban vs VKAs (n = 25,441)** | **Apixaban vs dabigatran**  **(n = 9,333)** | **Dabigatran vs rivaroxaban**  **(n = 9,315)** | **Apixaban vs rivaroxaban**  **(n = 46,845)** |
| --- | --- | --- | --- | --- | --- | --- |
| **Major bleed** | 0.427(0.387;0.471)  *p*<0.0001 | 0.505(0.421;0.607)  *p*<0.0001 | 0.716(0.641;0.799)  *p*<0.0001 | 0.664(0.534;0.826)  *p*<0.001 | 0.709(0.574;0.876)  *p*<0.001 | 0.526(0.477;0.579)  *p*<0.0001 |
| **GIB** | 0.428(0.361;0.507)  *p*<0.0001 | 0.928(0.694;1.242)  *p*=0.6156 | 0.918(0.767;1.097)  *p*=0.3460 | 0.327(0.224;0.478)  *p*<0.0001 | 1.077(0.782;1.484)  *p*=0.6486 | 0.394(0.334;0.465)  *p*<0.0001 |
| **ICH** | 0.464(0.388;0.554)  *p*<0.0001 | 0.217(0.14;0.337)  *p*<0.0001 | 0.45(0.369;0.55)  *p*<0.0001 | 2.086(1.311;3.319)  *p*<0.01 | 0.484(0.297;0.791)  *p*<0.01 | 0.869(0.719;1.05)  *p*=0.1454 |
| **Other bleed** | 0.38(0.322;0.447)  *p*<0.0001 | 0.393(0.289;0.535)  *p*<0.0001 | 0.745(0.631;0.881)  *p*<0.001 | 0.656(0.447;0.963)  *p*=0.0313 | 0.51(0.372;0.699)  *p*<0.0001 | 0.518(0.447;0.6)  *p*<0.0001 |
| **Stroke/SE** | 0.688(0.613;0.771)  *p*<0.0001 | 0.642(0.504;0.818)  *p*<0.001 | 0.717(0.633;0.811)  *p*<0.0001 | 1.072(0.842;1.363)  *p*=0.5736 | 0.882(0.695;1.121)  *p*=0.3049 | 0.872(0.782;0.972)  *p*=0.0134 |
| **SE** | 0.74(0.601;0.912)  *p*<0.01 | 0.698(0.456;1.067)  *p*=0.0970 | 0.748(0.597;0.936)  *p*=0.0112 | 1.301(0.864;1.96)  *p*=0.2077 | 0.91(0.572;1.45)  *p*=0.6920 | 0.936(0.762;1.149)  *p*=0.5271 |
| **Stroke (ischemic or hemorrhagic)** | 0.671(0.583;0.772)  *p*<0.0001 | 0.618(0.464;0.824)  *p*<0.01 | 0.701(0.602;0.816)  *p*<0.0001 | 1.004(0.75;1.343)  *p*=0.9795 | 0.865(0.652;1.147)  *p*=0.3136 | 0.843(0.739;0.961)  *p*=0.0108 |
| **Ischemic stroke** | 0.86(0.726;1.018)  *p=*0.0790 | 0.868(0.617;1.221)  *p*=0.4164 | 0.823(0.683;0.991)  *p*=0.0402 | 0.844(0.598;1.19)  *p*=0.3325 | 1.01(0.729;1.399)  *p*=0.9512 | 0.876(0.749;1.025)  *p*=0.0974 |
| **Hemorrhagic stroke** | 0.355(0.273;0.461)  *p*<0.0001 | 0.246(0.14;0.432)  *p*<0.0001 | 0.458(0.348;0.603)  *p*<0.0001 | 1.616(0.995;2.625)  *p*=0.0522 | 0.619(0.379;1.012)  *p*=0.0561 | 0.782(0.611;1.002)  *p*=0.0519 |

AFT, accelerated failure time; CI, confidence interval; GIB, gastrointestinal bleeding; ICH, intracranial hemorrhage; PS, propensity score; SE, systemic embolism; VKA, vitamin K antagonist.
