# Supplementary material for: Comparative safety and effectiveness of oral anticoagulants in key subgroups of patients with non-valvular atrial fibrillation and at high risk of gastrointestinal bleeding: A cohort study based on the French National Health Data System (SNDS)
Source: PLoS One. 2025 Jan 22;20(1):e0317895. doi: 10.1371/journal.pone.0317895 (PMC11753696; doi:10.1371/journal.pone.0317895)
Supplement: S8 Table — (DOCX) [file pone.0317895.s008.docx]

**S8 Table.** Estimated relative acceleration factors and 95% CI from the AFT analysis (PS matched population receiving concomitant medication)

|  | **Apixaban vs VKAs**  **(n = 26,155)** | **Dabigatran vs VKAs (n = 9,946)** | **Rivaroxaban vs VKAs (n = 22,619)** | **Apixaban vs dabigatran**  **(n = 10,145)** | **Dabigatran vs rivaroxaban**  **(n = 10,141)** | **Apixaban vs rivaroxaban**  **(n = 57,573)** |
| --- | --- | --- | --- | --- | --- | --- |
| **Major bleed** | 0.366(0.328;0.408)  *p*<0.0001 | 0.466(0.388;0.56)  *p*<0.0001 | 0.767(0.66;0.89)  *p*<0.001 | 0.752(0.608;0.93)  *p*<0.01 | 0.733(0.599;0.897)  *p*<0.01 | 0.489(0.445;0.538)  *p*<0.0001 |
| **GIB** | 0.373(0.307;0.452)  *p*<0.0001 | 0.87(0.657;1.152)  *p*=0.3304 | 0.83(0.686;1.005)  *p*=0.0561 | 0.468(0.333;0.657)  *p*<0.0001 | 1.146(0.84;1.564)  *P*=0.39 | 0.405(0.348;0.472)  *p*<0.0001 |
| **ICH** | 0.442(0.361;0.54)  *p*<0.0001 | 0.257(0.176;0.376)  *p*<0.0001 | 0.456(0.363;0.573)  *p*<0.0001 | 1.526(0.902;2.583)  *p*=0.1155 | 0.607(0.398;0.926)  *p*=0.02 | 0.84(0.701;1.006)  *p*=0.0583 |
| **Other bleed** | 0.305(0.254;0.367)  *p*<0.0001 | 0.319(0.23;0.442)  *p*<0.0001 | 0.719(0.601;0.861)  *p*<0.001 | 0.899(0.628;1.288)  *p*=0.5632 | 0.514(0.376;0.703)  *P*<0.0001 | 0.461(0.398;0.533)  *p*<0.0001 |
| **Stroke/SE** | 0.689(0.604;0.785)  *p*<0.0001 | 0.559(0.437;0.716)  *p*<0.0001 | 0.79(0.689;0.906)  *p*<0.001 | 0.936(0.701;1.25)  *p*=0.6528 | 0.897(0.7;1.149)  *p*=0.39 | 0.871(0.784;0.968)  *p*=0.0104 |
| **SE** | 0.871(0.695;1.09)  *p*=0.2273 | 0.554(0.363;0.846)  *p*<0.01 | 0.898(0.722;1.118)  *p*=0.3354 | 1.178(0.736;1.887)  *p*=0.4943 | 0.901(0.546;1.484)  *p*=0.68 | 1.041(0.856;1.266)  *p*=0.6894 |
| **Stroke (ischemic or hemorrhagic)** | 0.609(0.516;0.718)  *p*<0.0001 | 0.557(0.413;0.752)  *p*<0.001 | 0.733(0.617;0.871)  *p*<0.001 | 0.854(0.615;1.187)  *p*=0.3478 | 0.894(0.668;1.196)  *p*=0.45 | 0.802(0.705;0.912)  *p*<0.001 |
| **Ischemic stroke** | 0.729(0.595;0.893)  *p*<0.01 | 0.737(0.517;1.05)  *p*=0.0912 | 0.841(0.681;1.038)  *p*=0.1070 | 0.816(0.558;1.192)  *p*=0.2929 | 0.956(0.68;1.344)  *p*=0.80 | 0.829(0.71;0.968)  *p*=0.0178 |
| **Hemorrhagic stroke** | 0.381(0.284;0.51)  *p*<0.0001 | 0.26(0.147;0.463)  *p*<0.0001 | 0.515(0.377;0.704)  *p*<0.0001 | 0.875(0.455;1.683)  *p*=0.6884 | 0.759(0.45;1.281)  *p*=0.30 | 0.729(0.577;0.92)  *p*<0.01 |

AFT, accelerated failure time; CI, confidence interval; GIB, gastrointestinal bleeding; ICH, intracranial hemorrhage; PS, propensity score; SE, systemic embolism; VKA, vitamin K antagonist.
