# Supplementary material for: Comparative safety and effectiveness of oral anticoagulants in key subgroups of patients with non-valvular atrial fibrillation and at high risk of gastrointestinal bleeding: A cohort study based on the French National Health Data System (SNDS)
Source: PLoS One. 2025 Jan 22;20(1):e0317895. doi: 10.1371/journal.pone.0317895 (PMC11753696; doi:10.1371/journal.pone.0317895)
Supplement: S9 Table — (DOCX) [file pone.0317895.s009.docx]

**S9 Table.** Estimated relative acceleration factors and 95% CI from the AFT analysis (PS matched population with CKD stage 3 or 4)

|  | **Apixaban vs VKAs**  **(n = 5,908)** | **Rivaroxaban vs VKAs**  **(n = 2,638)** | **Apixaban vs rivaroxaban**  **(n = 2,650)** |
| --- | --- | --- | --- |
| **Major bleed** | 0.432(0.348;0.536)  *p*<0.0001 | 0.553(0.396;0.773)  *p*<0.001 | 0.812(0.473;1.392)  *p*=0.4482 |
| **GIB** | 0.448(0.31;0.648)  *p*<0.0001 | 0.79(0.463;1.349)  *p*=0.3883 | 0.485(0.249;0.945)  *p*=0.0335 |
| **ICH** | 0.435(0.278;0.68)  *p*<0.001 | 0.228(0.104;0.5)  *p*<0.001 | 1.647(0.712;3.812)  *p*=0.2436 |
| **Other bleed** | 0.398(0.287;0.552)  *p*<0.0001 | 0.582(0.355;0.952)  *p*=0.0312 | 0.542(0.296;0.993)  *p*=0.0473 |
| **Stroke/SE** | 0.668(0.521;0.858)  *p*<0.01 | 0.838(0.569;1.235)  *p*=0.3724 | 0.781(0.517;1.18)  *p*=0.2405 |
| **SE** | 0.836(0.575;1.217)  *p*=0.3510 | 1.442(0.802;2.594)  *p*=0.2215 | 0.614(0.329;1.145)  *p*=0.1248 |
| **Stroke (ischemic or hemorrhagic)** | 0.59(0.426;0.818)  *p*<0.01 | 0.627(0.383;1.024)  *p*=0.0622 | 0.916(0.554;1.515)  *p*=0.7323 |
| **Ischemic stroke** | 0.723(0.489;1.069)  *p*=0.1038 | 0.981(0.551;1.746)  *p*=0.9483 | 0.823(0.499;1.356)  *p*=0.4440 |
| **Hemorrhagic stroke** | 0.362(0.197;0.666)  *p*<0.01 | 0.229(0.08;0.66)  *p*<0.01 | 1.315(0.399;4.331)  *p*=0.6522 |

AFT, accelerated failure time; CI, confidence interval; GIB, gastrointestinal bleeding; ICH, intracranial hemorrhage; PS, propensity score; SE, systemic embolism; VKA, vitamin K antagonist.
